# Updated Guidelines for Best Practice in the Audiological Management of Adults with Severe and Profound Hearing Loss

**DOI:** 10.1055/s-0046-1827039

**Published:** 2026-10-26

**Authors:** Laura Turton, Barbra H. B. Timmer, Neil S. Hockley, Steven J. Aiken, Manohar Bance, Judith Bird, Katie Ekberg, Louise Hickson, Celia Hulme, Anja Kurz, Faheema Mahomed-Asmail, Min Cheol Roh, Nerina Scarinci, Grant D. Searchfield, Pamela Souza, De Wet Swanepoel, Linda M. Thibodeau

**Affiliations:** 1 NHS TaysideUnited Kingdom; 2 Sonova AG, StäfaSwitzerland; 3 School of Health and Rehabilitation Sciences, The University of QueenslandBrisbaneAustralia; 4 School of Communication Sciences and Disorders, Dalhousie UniversityHalifaxNova ScotiaCanada; 5 Department of Clinical NeurosciencesUniversity of CambridgeCambridgeUnited Kingdom; 6 Cambridge University Hospitals TrustCambridgeUnited Kingdom; 7 Audiology and Emmeline Hearing Implants, Cambridge University HospitalsCambridgeUnited Kingdom; 8 College of Nursing and Health Sciences, Flinders UniversityAdelaideAustralia; 9 University of ManchesterManchesterUnited Kingdom; 10 Comprehensive Hearing CenterDepartment of Oto-Rhino-LaryngologyHead and Neck Surgery, University Hospital WuerzburgGermany; 11 Department of Speech-Language Pathology and AudiologyUniversity of PretoriaPretoriaSouth Africa; 12 Sonova Communications AGMurtenSwitzerland; 13 Eisdell Moore Centre, Audiology, The School of Population Health, Faculty of Medical and Health Sciences, The University of AucklandAucklandNew Zealand; 14 TrueSilence Therapeutics Inc.SmyrnaGeorgiaUnited States; 15 Department of Communication Sciences and DisordersNorthwestern UniversityEvanstonIllinoisUnited States; 16 Virtual Hearing Lab, Collaborative initiative between University of Colorado School of Medicine, Aurora, and University of PretoriaPretoriaSouth Africa; 17 Department of Otolaryngology–Head and Neck SurgeryUniversity of Colorado School of MedicineAuroraColoradoUnited States; 18 Behavioral and Brain Sciences, University of Texas at Dallas, Callier Center for Communication DisordersDallasTexasUnited States

## Definitions and Scope

For the purposes of these guidelines, the following World Health Organization definitions apply:

- Severe hearing loss: an average hearing threshold of 61 to 80 dB HL (ISO) in the better ear.
- Profound hearing loss: an average hearing threshold of 81 dB HL (ISO) or greater in the better ear.
- Adult: any individual aged 19 years or older.
- Best practice: a technique or methodology that, through experience and research, has been shown to reliably achieve the desired outcome.

These guidelines focus on adults with severe or profound sensorineural hearing loss in the better ear. Conductive hearing loss is largely excluded. Individuals with precipitous or asymmetrical hearing loss are included when the better ear meets the threshold for severe hearing loss.

Associated characteristics of severe and profound sensorineural hearing loss, such as marked recruitment and tinnitus, are also considered. Each section addresses both acquired and congenital hearing loss and is relevant to adults who use either spoken language or sign language.

## Objective

These guidelines are designed to support hearing care professionals (HCPs) involved in the audiological management of adults with severe and profound hearing loss. While the management of mild-to-moderate hearing loss is well established, these approaches alone are often insufficient to meet the complex needs of adults with more significant hearing loss.

The purpose of this document is to outline the additional considerations, adaptations, and best practices required to optimize outcomes for adults with severe and profound hearing loss and their families and communication partners. Its primary goal is to provide evidence-based recommendations tailored specifically to this population. If hearing health professionals put these recommendations into practice, it will improve outcomes, standardize care, and support HCPs working with a highly diverse and complex population, with recommendations tailored specifically to their needs. This update was required due to new evidence, new technologies, and evolving client needs. This edition reviews evidence published between 2020 (the release of the first guideline) and the first quarter of 2026. In addition to revisiting all original topics, the scope has been expanded to include:

- Sign language users.
- Electrophysiology.
- Hearing aid technology and music.
- Public broadcast protocols (Auracast).
- Considerations for cochlear implants and other implantable devices.
- Remote fittings.
- Genetic hearing loss and emerging gene therapies

Each section is supported by additional tools to assist HCPs in applying the recommendations:

- Appendix 1—Evidence: Summary tables of evidence applicable to each recommendation (from first and second editions)
- Appendix 2—Bibliography: Divided into new evidence (2020–2025, listed alphabetically) and previous evidence (all references from version 1 of the guideline, listed alphabetically).
- Appendix 3—Toolkit tables: Signposting to practical tools relevant to each topic.
- Appendix 4—Clinical checklists: Resources for professionals and services to benchmark current practice against the recommendations and develop action plans for compliance.

## Contents

**Table:** 

**Setting the Scene: Fundamentals**	
I. Introduction	
I.i Prevalence	
I.ii Causes	
I.iii Person-centered management of clients with severe and profound hearing loss	
I.iv Consequences of severe and profound hearing loss	
I.v Auditory characteristics shaping amplification needs	
I.vi Unique amplification and rehabilitation needs	
II. Methods	
II.i How the guidelines were developed	
II.ii Research evidence for adults with bilateral severe and profound hearing loss	
II.iii Evidence	
II.iii.i First edition of the guidance (2020)	
II.iii.ii Second edition of the guidance (2026)	
**Updated Guidelines and Recommendations**	
1. Supporting accessibility and deaf awareness	
1.1 Accessibility	
1.2 Deaf awareness	
2. Support and integration of the audiological management of adults with severe and profound hearing loss who use sign language	
3. Audiological assessment of severe and profound hearing loss	
3.1 Understanding communication needs and the impact of hearing loss	
3.2 Nonauditory needs assessment	
3.3 Diagnostic assessment with behavioral testing	
3.4 Diagnostic assessment with objective testing	
3.5 Developing a comprehensive treatment plan	
4. Technology to support severe and profound hearing loss	
4.1 Prescribing and fitting hearing aids	
4.1.1 Compression	
4.1.2 Device choices and programs	
4.1.3 Frequency lowering for adults with severe and profound hearing loss	
4.1.4 Prescriptions and verification	
4.1.5 Selecting technology for asymmetrical severe and profound loss	
4.1.6 Maximum power output and threshold shift	
4.1.7 Considerations for music	
4.2 Prescribing and fitting remote microphones	
4.2.1 Considerations for recommending and managing ongoing use of remote microphone systems	
4.2.2 Component considerations	
4.2.3 Considerations regarding remote microphone verification	
4.3 Relevant mobile apps	
4.4 Alerting devices and other assistive listening solutions	
4.5 Public broadcast protocols (Auracast)	
4.6 Cochlear implant candidacy and bimodal hearing	
4.6.1 Level of awareness of cochlear implantation	
4.6.2 Clinical effectiveness of cochlear implants	
4.6.3 Cost-benefit of cochlear implants	
4.6.4 Understand the benefits of bimodal fittings	
4.6.5 Understand the limitations of other implantable devices for this population	
4.7 Remote fittings	
4.7.1 Remote initial fittings	
4.7.2 Remote fine-tuning and follow-up	
4.7.3 Client and HCP perspectives	
4.7.4 Infrastructure and future directions	
4.7.5 Safety and risk management	
4.7.6 Special considerations for severe and profound hearing loss	
5. Rehabilitation and other treatment considerations	
5.1 Rehabilitation: Communication and psychosocial concerns	
5.1.1 Addressing the individual psychosocial impact of severe and profound hearing loss	
5.1.2 Incorporating family in rehabilitation for shared psychosocial well-being	
5.1.3 Building psychosocial support in the community and workplace	
5.1.4 Addressing psychosocial impact for Deaf sign language users	
5.2 Tinnitus	
5.2.1 Definitions, prevalence, and generalization of guidelines to this population	
5.2.2 Tinnitus Management	
5.2.2.1 Assessment and referrals	
5.2.2.2 Medical management	
5.2.2.3 Psychological therapies	
5.2.2.4 Hearing assistance	
5.2.2.5 Sound-based therapies	
5.2.2.6 Other therapies	
5.3 Genetic hearing loss and gene therapy for genetic hearing loss	
5.3.1 Candidate identification and diagnostic workup	
5.3.2 Gene therapy guidelines	
5.3.2.1 Predosing	
5.3.2.2 Intraoperative procedures	
5.3.2.3 Postsurgical monitoring	
6. Ongoing care and support	
6.1 Measurement of client-reported outcomes and assessment of treatment goals	
6.2 Assessing the need for onward referral	
6.3 Ensuring appropriate ongoing management	
7. Summary, future research considerations, and conclusions	
7.1 Future directions	
7.1.1 Sign language users	
7.1.2 Assessment	
7.1.2.1 Understanding variability in speech perception	
7.1.2.2 Cochlear implant candidacy and access	
7.1.2.3 Objective identification of cochlear dead regions	
7.1.2.4 Outcome measures	
7.1.3 Prescribing and fitting hearing aids	
7.1.3.1 Hearing aid technology for severe and profound hearing loss	
7.1.3.2 Options for music	
7.1.4 Public broadcast (Auracast) and assistive listening in public spaces	
7.1.5 Remote fitting and teleaudiology	
7.1.6 Tinnitus in severe and profound hearing loss	
7.2 Summary and conclusion	
7.3 Declaration of interests from the authors	
7.4 Date for review of guidelines	
**Appendices**	
Appendix 1—Evidence tables	
Appendix 2—Bibliography	
Appendix 3—Toolkit tables	
Appendix 4—Clinical checklists	
Appendix 5—QuADS criteria	

## I. Introduction

“In many ways patients with severe hearing loss are the most interesting we see, calling upon our skills as clinicians to develop assistive strategies, provide counseling, and think more creatively than the typical hearing aid fitting. As clinicians, we understand that the end result of a hearing aid fitting is limited by the processing capability of the peripheral and central auditory system, and that few patients with severe sensorineural hearing loss will achieve high levels of speech recognition in complex listening situations.” (Souza, 2009, p. 8).

Adults with severe and profound hearing loss represent a highly diverse population whose clinical needs extend well beyond the scope of conventional audiological management (Turton et al, 2020). Communication outcomes are shaped not only by auditory capacity, but also by access to language, including spoken and signed languages, and by the environments in which communication takes place (Hulme et al, 2022). Severe and profound hearing loss is associated with markedly reduced access to the acoustic speech signal, due to complex auditory physiology (Souza, 2002). These factors create unique challenges that require specialized assessment, technology optimization, and extensive rehabilitation requirements.

### I.i Prevalence

Due to the specialist skills required for these clients, it is important to understand the prevalence of this population.

The World Health Organization's World Report on Hearing highlights the growing global burden of hearing loss, noting that it is “a widely prevalent health condition of diverse etiology, affecting individuals across the life course.” Driven by demographic change and persistent risk factors, prevalence continues to rise. By 2050, nearly 700 million people are projected to experience hearing loss of moderate or greater severity in the better ear. In global terms, this equates to almost one in four people having some degree of hearing loss, and approximately one in 14 (at least 7%) requiring hearing care (WHO, 2021, p. 141).

Applying the WHO definition of severe hearing loss (>60 dB HL in the better ear), prevalence is estimated at approximately 0.28 to 2.2% of the general population (Cruickshanks et al, 1998; Löfvenberg et al, 2022; Sindhusake et al, 2001). Using a more conservative threshold (>70 dB HL), prevalence estimates decrease to around 0.7% (Carlsson et al, 2014; Turton & Smith, 2013). Regardless of the definition applied, the number of adults affected increases sharply with age, peaking in the eighth decade of life for both men and women. Within typical hearing aid services, it is anticipated that up to 6.7 to 20% of adults with hearing loss will have a severe or profound degree of impairment (Carlsson et al, 2014; Turton & Smith, 2013).

Audiometric prevalence does not predict communication modality or language use, and individuals within these prevalence estimates may use spoken language, sign language, or a combination of communication modalities.

### I.ii Causes

Factors which can lead to severe and profound sensorineural hearing loss include age, noise exposure, congenital and genetic conditions, ototoxic drugs, and injuries such as head trauma. Diseases include meningitis, viral and autoimmune diseases, advanced otosclerosis, and Meniere's disease. The hearing loss onset can be sudden or progressive (Hughes & Pensak, 2015). Age of onset and progression are clinically relevant factors, as early-onset and long-standing hearing loss may be associated with differing communication experiences, language preferences, and support needs compared to later-onset hearing loss.

### I.iii Person-Centered Management of Clients with Severe and Profound Hearing Loss

Adults presenting to audiological services are often older individuals with age-related hearing loss who primarily use spoken language (NHS, 2016). However, hearing care professionals also support culturally Deaf adults whose first or preferred language is sign language, and whose communication needs and expectations may differ substantially from those of spoken-language users. Person-centered care requires explicit exploration of an individual's listening goals, recognizing that not all hearing aid users seek improved access to speech (BAA, 2025). For some adults, amplification may instead be used to support lipreading, enhance environmental awareness, manage tinnitus, or facilitate engagement with music (Hulme, 2022). These goals should be identified early and integrated into assessment, fitting, and follow-up processes (NHS, 2016). Person-centered and family-centered principles are emphasized. Person-centered care in audiology places the individual's needs, values, communication preferences, and lived experience at the center of assessment and rehabilitation. It prioritizes shared decision-making, respectful communication, and tailoring interventions to each person's goals rather than relying on a purely device-focused model. Family-centered care extends this approach by recognizing that hearing loss is experienced within a social context and often affects communication partners and family members as well as the individual. A family-centered approach involves actively engaging significant others in assessment, goal setting, education, and rehabilitation where appropriate, while respecting the autonomy and preferences of the person with hearing loss. Evidence demonstrates that involving family members can improve communication outcomes, adherence to management strategies, and psychosocial wellbeing for both the individual and their communication partners (Meyer et al, 2019). Person-centered approaches in adult auditory rehabilitation improve satisfaction, engagement, and functional outcomes by fostering active involvement in care planning and rehabilitation processes (Graham et al, 2025). This aligns with the World Health Organization's recommendation that person-centered care should underpin all modern hearing healthcare practice and approaches in adult auditory rehabilitation, to improve satisfaction, engagement, and functional outcomes by fostering active involvement in care planning and rehabilitation processes (Graham et al, 2025). Adults presenting to hearing aid services are often older individuals with age-related hearing loss who primarily use spoken language (NHS, 2016). However, audiometric severity alone does not predict communication preference, linguistic identity, or desired outcomes from intervention. Assessment must therefore explicitly explore how individuals communicate, their listening environments, and their priorities for rehabilitation, alongside hearing thresholds.

HCPs also support adults with severe and profound hearing loss who use sign language as a primary or preferred mode of communication. For these individuals, person-centered care requires recognition that auditory intervention may play a different role, or a more limited role, within a broader communication repertoire.

In working with every client, hearing health professionals should explore personal preferences, communication identities, and cultural contexts to demonstrate cultural competence, particularly when supporting underrepresented groups and cultural minorities who may not be typical users of acoustic hearing aids (Hulme et al, 2023). Throughout these guidelines, both person–centered and family–centered principles are emphasized.

Recent guidance from the British Society of Audiology (2025) reinforces that effective hearing care must acknowledge the broader impact of hearing loss on families and communication partners and provide personalized, holistic support.

Overall, adults with severe and profound hearing loss constitute a highly diverse population, with considerable variation in etiology, language use, communication modality, linguistic identity, and functional impact. This diversity necessitates flexible, individualized, and culturally responsive approaches to audiological management, supported by both medical and social models of care.

### I.iv Consequences of Severe and Profound Hearing Loss

The consequences of this degree of hearing loss change life experience and opportunities, including those for the people closest to those with the hearing loss. Communication partners play a critical role, as the impact of communication breakdowns can be significant for both the individual and their wider social network (Grenness et al, 2014; Scarinci et al, 2012; Singh et al, 2016). Severe and profound hearing loss negatively affects quality of life, regardless of age or rapidity of onset (Carlsson et al, 2014) and negatively impacts activities of daily living (Gopinath et al, 2013; Turton & Smith, 2013). Several studies have identified higher levels of social isolation, anxiety, and depression among adults with severe and profound hearing loss, compared to their better hearing peers (Grimby & Ringdahl, 2016; Hallam et al, 2006). These psychosocial outcomes are strongly mediated by access to effective and preferred communication. Individuals with timely access to sign language and strong Deaf social networks may experience fewer negative consequences than those with unmet communication needs.

Young people with severe and profound hearing loss are less likely to go to university or work full time compared to their hearing peers (Cameron et al, 2008). As these adults age, they may struggle with other attributes of ageing, such as declining vision, mobility, dexterity, cognition and general health. In addition to a social support network of friends and family (Turton & Smith, 2013), to avoid poor health and social isolation, hearing healthcare has an important role in the life of the person with hearing loss, to support them and provide effective tools for communication (Carlsson et al, 2015; Turunen-Taheri et al, 2018).

### I.v Auditory Characteristics Shaping Amplification Needs

Adults with severe and profound hearing loss require high output, low distortion devices capable of delivering substantial gain while maintaining comfort, alongside carefully controlled wide dynamic range compression. Robust feedback management with tightly sealed earmolds is essential to achieve prescribed gain (Dillon, 2012; Souza, 2002). Signal-to-noise ratio (SNR) enhancement is also necessary through directional microphones and remote microphone systems, as this population often requires +10 to +20 dB SNR for functional speech understanding (Killion, 2002). These consequences extend beyond audibility. The etiology of severe and profound hearing loss is complex, with extensive cochlear damage, including outer and inner hair cell loss, auditory neuropathy/synaptopathy, broadened auditory filters, and/or impaired temporal fine structure processing alongside limited residual hearing (Moore, 2007, 2016). Individuals frequently experience reduced frequency resolution, poor frequency selectivity and distortion due to cochlear dead regions, severe recruitment, tinnitus, and increased listening effort, all of which can limit the benefit of amplification and contribute to communication fatigue, although the extent of the impairment varies widely among individuals (Ohlenforst et al, 2017; Messersmith, 2018; Souza, 2002; Souza & Hoover, 2018; Souza et al, 2020).

These auditory characteristics help explain why amplification outcomes are highly variable in this population and why audibility alone does not equate to effective communication. Many adults with severe and profound loss also will experience tinnitus (Carlsson et al, 2014), which negatively impacts quality of life for some individuals and may further complicate hearing aid use.

For some adults, particularly those who are sign language users, auditory listening may require substantially greater cognitive effort. This can influence patterns of hearing aid use, perceived benefit, and decisions about when and how amplification is used (Hulme et al, 2022). Importantly, reduced reliance on amplification for speech does not reflect a lack of need but rather a different balance between auditory and visual communication strategies. These additional complexities within the auditory system mean that adding gain will not eliminate communication problems. HCPs need to consider that the result of a hearing aid fitting is limited by the processing capability of the peripheral and central auditory system and that few clients with severe sensorineural hearing loss will achieve high levels of speech recognition in complex listening situations (Souza, 2020). These physiological changes fundamentally alter how amplified sound is perceived, requiring tailored approaches to hearing aid fitting and verification.

### I.vi Unique Amplification and Rehabilitation Needs

Such clients do not easily fit into our regular routines for hearing care: “clients with severe loss are also the best illustration of the complexities of the auditory system and remind us (yet again) that adding gain is not a simple solution to communication problems” (Souza, 2009, p. 8). Even as technologies improve, “hearing aids may never be fully sufficient for those with severe cochlear damage” (Lesica, 2018, p. 185).

People with severe and profound hearing loss are often long-term, full-time users of ampliﬁcation who, because of their degree of loss, are highly reliant on their devices. Their ampliﬁcation needs are unique in that they require a wide range of input levels to be made audible, comfortable, and safe within a narrow range of residual hearing (Convery & Keidser, 2011; Munro et al, 2016). However, patterns of hearing aid use vary across this population. Sign language users may use amplification in a targeted or situational manner rather than for continuous speech-based communication (Hulme et al, 2021, 2022).

High-powered hearing aid technology has drastically improved over the last two decades; however, hearing aids may never be fully sufficient for people with severe cochlear damage (Lesica, 2018). Conversely, clients with severe conductive hearing loss often have very good speech discrimination and get very good results from amplification.

Therefore, once optimal aiding is established, other treatment options must be considered. Assistive technologies and hearing dogs may be relevant considerations for this population. The benefits of wireless microphone technology have been well established for severe and profound hearing loss (Thibodeau & Schaper, 2014) and can be considered as a standard component of a rehabilitation program (Thibodeau, 2019a). Persistent poor aided speech recognition despite these measures is a critical indicator for cochlear implant evaluation (Gifford, 2013). People with severe and profound hearing loss pose unique hearing, psychosocial, and communication rehabilitation needs. Best practice therefore requires a broad definition of rehabilitation, incorporating hearing technology, assistive devices, and support for effective communication strategies. The authors have sought to address these challenges within the recommendations throughout this Practice Guidance.

## II. Methods

### II.i How the Guidelines Were Developed

These guidelines outline best practice in the audiological management of adults with severe and profound hearing loss. Best practice will be defined using a two-stage approach: (1) use evidence where available, and elsewhere (2) provide consensus advice from an expert panel. Where evidence is available, it will provide the evidence for best evidence-based practice, and where the scientific evidence is insufficient, the guide provides specific recommendations based on expert advice. The authors hope that sharing this information will inform best practice in hearing care and improve outcomes for adults with severe and profound hearing loss.

Specific statements, recommendations and strategies were made by initially reviewing the existing scientific evidence published in peer-reviewed and nonpeer-reviewed journals. When direct evidence was not available, indirect evidence (often evidence from mild-to-moderate hearing loss, pediatrics or cochlear implants) was used, and consensus on practice was considered in making recommendations.

The process of developing recommendations was evidence-based when possible. Where evidence is ambiguous or conflicting, or where scientific data are lacking, the clinical expertise of the authors was used to guide the development of consensus-based recommendations.

The methodology used in developing the guidelines is drawn from the 2018 revision of the 2016 NHMRC Standards for Guidelines, National Health and Medical Research Council of Australia. In addition, useful information was provided by Rosenfeld et al (2013), Clinical Practice Guideline Development Manual, Third Edition: A Quality-Driven Approach for Translating Evidence into Action, Otolaryngology–Head and Neck Surgery 148(1S):S1–S55, and the American Academy of Audiology Clinical Practice Guideline: Pediatric Amplification (June, 2013).

This paper presents best practice guidance by experts in the fields of audiology and otology with specialized knowledge in the management of severe and profound hearing loss. It encompasses the evidence base and consensus on good practice, given the stated methodology and scope of the document and at the time of publication. Internationally, there are many general guidelines for assessment and audiological management for all adults with hearing loss. Rarely, if ever, are people with a severe and profound hearing loss referred to specifically in any of these guidelines.

### II.ii Research Evidence for Adults with Bilateral Severe and Profound Hearing Loss

Although often highlighted in this guidance, it is important to emphasize the limited research that has focused on this population over the last 10 to 15 years. Since the first edition was published, there have been updates in the research as well as the addition to this second edition of new topics in Audiology covering sign language users, electrophysiology, hearing aid technology and music, public broadcast protocols (Auracast), considerations for cochlear implants and other implantable devices, remote fittings and genetic hearing loss and emerging gene therapies.

Within the summary and conclusion is a section where authors have called out specific research gaps, in the hope that these will be addressed prior to a third edition in the future.

### II.iii Evidence

Appendix 1 outlines the evidence from which the recommendations are based. These guidelines are not intended to be a systematic review. Instead, the authors searched the literature to identify the best available evidence to provide support for the development of key recommendations.

#### II.iii.i First Edition of the Guidance (2020)

In searching the literature, the authors first sought to identify studies at the top of the hierarchy of study types (2c Levels of evidence). The authors then graded the evidence using the rating scheme described below (2d Grades of recommendation). In addition, the authors identified whether the data provided effective (EV) or efficacious (EF) information. EV is evidence measured in the real world, while EF is evidence measured under laboratory or ideal conditions (2e EF/EV). Finally, if the authors had to extend their literature search beyond adults with severe and profound hearing loss, this is identified by evidence from elsewhere (2f Supporting evidence). All authors reviewed the recommendations and evidence grading for the Practice Guidance and agreed on the levels of quality assigned. This assessment of the literature is based on the recommendations for evidence-based practice in the provision of amplification (Cox, 2005a) as implemented in the AAA Pediatric Amplification Guidelines (2013). The results of the assessment are collated in an evidence table as follows (see Table 1 ):

**Table 1:** Evidence table format from the original 2020 guideline publication

Rec. (recommendation number)	Evidence	Source (reference)	Level of evidence	Grade	EF/EV	MM/P/CI
	Statement from reference	Reference	1. Systematic reviews and meta-analyses of randomized controlled trials 2. Randomized controlled trials 3. Nonrandomized intervention studies 4. Descriptive studies (cross-sectional surveys, cohort studies, case-control designs) 5. Case studies 6. Expert opinion	A = Consistent level 1 or 2 studies B = Consistent level 3 or 4 studies or extrapolations from level 1 or 2 studies C = Level 5 studies or extrapolations from level 3 and 4 studies D = Level 6 evidence or troubling inconsistencies or inconclusive studies at any level	Efficacy (EF) or Effectiveness (EV). EF is evidence measured under “laboratory or ideal” conditions and EV is evidence measured in the “real” world	Evidence given for mild-to-moderate hearing loss (MM), pediatrics (P) or cochlear implants (CI) was considered for inclusion

#### II.iii.ii Second Edition of the Guidance (2026)

The level of evidence supporting the recommendations in the 2020 Guidelines was not re-assessed for this updated version. However, given the heterogeneity of audiology and hearing science research design and reporting, for this updated version of the Guidelines it was decided to utilize the Quality Assessment with Diverse Studies (QuADS) tool to assess the level of evidence for any research newly incorporated in the Guidelines (Harrison et al, 2021).

QuADS is an appraisal instrument designed to evaluate the methodological and reporting quality of studies in mixed-method or multi-method health services research. The QuADS tool contains 13 criteria. Each criterion was given a score between 0 (lowest) and 3 (highest). The tool therefore has a maximum possible score of 39. The QuADS criteria include the following.

1. Theoretical or conceptual underpinning to the research.
2. Statement of research aim/s.
3. Clear description of research setting and target population.
4. The study design is appropriate to address the stated research aim/s.
5. Appropriate sampling to address the research aim/s.
6. Rationale for choice of data collection tool/s.
7. The format and content of the data collection tool is appropriate to address the stated research aim/s.
8. Description of data collection procedure.
9. Recruitment data provided.
10. Justification for analytic method selected.
11. The method of analysis was appropriate to answer the research aim/s.
12. Evidence that the research stakeholders have been considered in research design or conduct.
13. Strengths and limitations critically discussed.

The strength of the QuADS tool includes its applicability to quantitative, qualitative and mixed-method design, and its reflection of modern research approaches such as lived experience and other stakeholder involvement.

In updating this version of the Guidelines, each contributing author was asked to review the research evidence for their perspective section that had been published after 2020 and/or not incorporated in the 2020 version of this Guideline. Any research provided as evidence for the recommendations contained in this Guideline was then rated using the QuADS tool. The quality of evidence rating for the newly incorporated studies can be found in Appendix 1. In the case of section authors also being authors of the cited evidence, other contributors were asked to rate specific studies to mitigate any conflict of interest or bias in the evidence assessment.

## Updated Guidelines and Recommendations

## 1. Supporting Accessibility and Deaf Awareness

**Table:** 

Effective hearing care professional–client communication is fundamental to safe and equitable healthcare. It has implications for: Meeting legal obligations Reducing health inequalities Supporting a cost-effective health service Improving client satisfaction with care Providing safe client care

Deaf awareness enables providers to meet the communication needs of adults with severe and profound hearing loss. Deaf awareness within healthcare is essential not only for people who have severe and profound hearing loss but also for anyone with diagnosed or undiagnosed hearing loss, whether this is disclosed or recorded. Communication failures contribute to medical errors, compromised safety, and reduced trust across diverse healthcare systems, and persistent gaps in Deaf awareness have been documented (Hulme et al, 2021, 2022, 2023; Jama et al, 2019; Parmar et al, 2022, 2025), where similar barriers to accessible communication and culturally competent care have been reported (Kuenburg et al, 2016; McKee et al, 2015).

### 1.1 Accessibility

Deaf awareness underpins accessible engagement between clients and HCPs. This section focuses on systems, structures, and processes that enable clients to access the clinical environment equitably.

Recommendations (see evidence Appendix 1: Supporting Accessibility and Deaf Awareness, 1; Section 1.1)

Ensure the clinical environment is accessible by:

1. Understanding any legal frameworks in place for your country and area of practice to ensure legal compliance with existing legislation.
2. Determine access needs by requesting information from the referring services and directly from the client. These should be documented in the client record and shared across services, where possible, and should be referenced at every contact.
3. Consider that access to care might require involvement of a registered sign language interpreter, a lipspeaker, a language translator, and use of live speech-to-text applications. When preferred support cannot be provided, this should be discussed with the client, documented, and mitigated wherever possible.
4. Achieve accessibility by ensuring that all staff who have client contact undergo Deaf awareness training no matter what their role every 3 years.
5. Ensure there is a wide range of methods (which staff are trained in) in which the service can be contacted: email/text message/video remote interpreting (VRI)/online booking systems as well as by face-to-face and phone communication.
6. Ask the client whether they prefer remote (telephone/video) or in-person appointments and schedule accordingly. It may be that they cannot hear over the phone or via a remote appointment option, or that you need to provide a remote option with captions and camera on for accessibility.
7. Ensure that the client receives information in an accessible format and any communication support they need, for example, easy-read format appointment letters.
8. Services should consider how accessible communication will be provided during unplanned, urgent, or short notice appointments, where usual access arrangements may not be in place.

### 1.2 Deaf Awareness

For adults with severe and profound hearing loss, ensuring that services are accessible and delivered with appropriate Deaf awareness is the foundation on which all other clinical recommendations depend. If a client cannot access or communicate effectively with their hearing healthcare provider, the value of any subsequent recommendations is compromised. Prioritizing accessibility and Deaf–aware practice are essential to meet legal obligations, reduce health inequalities, support cost–effective service delivery, improve client satisfaction, and ensure safe care. Deaf awareness includes understanding Deaf people, particularly sign language users, as a linguistic and cultural minority, rather than viewing Deafness from a medical perspective. Deaf-aware practice also recognizes the additional effort and communication burden often placed on Deaf clients when navigating healthcare interactions.

Recommendations (see evidence Appendix 1: Supporting Accessibility and Deaf Awareness, 1; Section 1.2)

Ensure the clinical environment is Deaf aware by:

1. Ensuring access to the building is not solely reliant upon hearing (e.g., intercom)
2. Installing amplification systems when transparent barriers are in place and for those using hearing aids, a fully functional loop or public broadcast (Auracast) system which all staff should test regularly and understand how to use.
3. Reception staff having awareness of how to engage with clients with severe and profound hearing loss effectively and respectfully.
4. HCPs offering the use of visual or tactile communication tools in the waiting room to alert the person when it is their appointment. This could include the use of a visual alerting system, a whiteboard, vibrating pagers, etc.
5. Setting up appropriate seating arrangements in the waiting area so clients with severe and profound hearing loss have an optimal view of the reception and main activity areas.
6. Confirming documented communication preferences and asking them what communication method works best for them in the appointment.
7. Ensuring the clinic room has minimal background noise, good lighting, and provides confidentiality if you are raising your voice when communicating.
8. Using communication tactics including checking for understanding, rephrasing, not shouting, and writing things down/typing things in a Word document/using live speech-to-text apps on tablets and phones can be particularly helpful. These strategies should be used proactively rather than only when communication difficulties arise. See Table 2 .
9. Ensuring key clinical decisions and recommendations are written down, included in reports for the client, or provided in an accessible information format to support understanding, recall, and follow-up.

**Table 2:** Communication tactics for positive communication

**Best practice for communicating with clients with severe and profound hearing loss** The HCP's communication style • Ask the client what communication method works best for them. Do not assume. • Ensure that you are within two meters of the client when communicating with them. • Make sure you get the attention of the client before you start talking, and you are facing each other o Do not speak directly to the computer you might be using; face the client whenever you are communicating with them and do not hide your face or lips. • If they have hearing aids (or other devices such as cochlear implants), check whether the client is wearing them and if they are working. Also understand that they may still have residual communication needs when using hearing aids. • Check they can understand you and are following what you are saying. • Speak clearly and audibly. Ensure that your facial expression mirrors what you are saying. • If the client is a sign language user, ensure there is a registered/certified interpreter present. The environment you are in • Reduce the background noise as much as possible—hearing aids will amplify everything, including background noise. • Ensure the lighting is good and that your face is visible to the client. • Ensure that light is on your face rather than behind you. • Consider your location in terms of privacy if you are communicating with a client. If the client is struggling to understand you Other things to consider are: • Be patient if someone is struggling to follow the conversation. • Reduce the distance between you and the client. • Don't shout as this can distort your voice and your lip patterns. • Rephrase what you are saying—repeat what you need them to know in a different way and use plain English wherever possible. o Do not say “it doesn't matter” but repeat or rephrase things so the client can understand you. • Writing things down/typing things in a Word document/using live speech-to-text apps on tablets and phones can be incredibly helpful

(See Appendix 3: Toolkit; Table 1 .)

(See Appendix 4: Clinical Checklist 1.)

## 2. Support and Integration of the Audiological Management of Adults with Severe and Profound Hearing Loss Who Use Sign Language

**Table:** 

These principles operationalize the person-centered and culturally responsive care framework described in Introduction section. Audiometric severity does not predict communication modality or identity. Use of sign language does not preclude benefit from amplification, cochlear implants, or assistive technology, but may influence how, when, and in which contexts these tools are used. Audiological success should be defined in relation to the individual's communication goals, not solely speech-based outcomes

Adults with severe and profound hearing loss may use sign language as a primary or preferred mode of communication. This includes individuals who identify as culturally Deaf and may view their hearing loss not as a disability but as a linguistic and cultural identity.

As outlined earlier, audiometric thresholds alone do not predict communication needs, communication modality, or desired rehabilitation outcomes. This section applies those person-centered and biopsychosocial principles to adults who use sign language.

For foundational accessibility measures and general Deaf awareness strategies (e.g., visual alerts, loop systems, basic communication techniques), see Section 1: Supporting Accessibility and Deaf Awareness.

Audiological management for sign language users must be culturally responsive and tailored to communication preferences that may not prioritize auditory speech perception. This section builds on the severe-to-profound hearing loss guidance, focusing on specialist considerations for sign language users, including cultural competence, interpreter use, and individualized rehabilitation planning.

Best practice requires a shift away from an exclusively biomedical, speech-restoration framework toward a biopsychosocial and cultural model of care, consistent with the rehabilitation framework described in Section 1.

The section complements, rather than duplicates, the core guidance on severe and profound hearing loss. It provides focused recommendations to support HCPs in delivering culturally competent, person-centered audiological care to adults who are sign language users. This includes respecting linguistic choices, ensuring accessible communication, and recognizing that intervention goals may prioritize environmental sound awareness, safety, or situational listening rather than speech perception (Hulme et al, 2022).

Recommendations (see evidence Appendix 1: Supporting Accessibility and Deaf Awareness, 2; Section 2.1)

1. Organizational commitment and policy:
  - Develop and implement a formal organizational policy on cultural competency and accessibility, endorsed by leadership.
  - Allocate a dedicated budget for communication access (e.g., interpreters, sign language resources) and for staff training.
  - Align organizational policies with the broader severe-profound hearing loss guidance to ensure equity across communication modalities.
2. Accessible environment and technology—building on the accessibility standards detailed in Section 1, services should ensure the clinical environment is Deaf-friendly by:
  - Providing visual alert systems (e.g., waiting room alerts)
  - Providing assistive hearing technology where appropriate.
  - Having accessible information in visual and written formats
  - Ensuring that environmental accessibility should be considered a core component of rehabilitation, alongside hearing aids and assistive listening devices.
3. Providing culturally competent client Care
  - Respecting the cultural and linguistic identity of Deaf Sign Language users. Avoid framing hearing loss as a deficit to be corrected.
  - Undertaking shared decision-making through assessing the individual communication preferences and goals, recognizing that these may differ from speech-focused rehabilitation pathways outlined elsewhere in this guidance.
  - Using communication strategies: Use effective communication strategies tailored to sign language users, including direct engagement, visual supports, text-based tools, and plain language.
4. Work with qualified interpreters. Provide qualified interpreters to ensure informed consent, accurate history-taking, and effective counseling. Responsibility for arranging interpreters rests with the service provider, in line with legal and professional obligations referenced in Section 1.
5. Technology discussions: frame discussions of hearing aids, assistive devices, and cochlear implants within the context of the individual's life as a sign language user, considering functional goals, communication preferences, and the broader technological principles outlined in the main guidance.
6. Staff training and development
  - Implement regular, mandatory training on working with sign language users, developed and delivered in collaboration with local Deaf organizations.
  - Move beyond generic Deaf awareness training to targeted skill-based training aligned with person-centered rehabilitation principles described in Introduction section.
7. Collaboration and Support Services
  - Collaborate with deaf communities, clubs, and charities to inform service design and delivery.
  - Provide referrals to culturally appropriate counseling and peer support services where needed.
  - Where appropriate, coordinate audiological care with wider social, educational, and community-based supports.
8. Data collection and equity
  - Systematically collect data on communication modality and preferred language to monitor equity of access and outcomes.
  - Outcome measures should reflect functional benefit and participation, not solely speech perception scores.

(See Appendix 3: Toolkit; Table 1 .)

(See Appendix 4: Clinical Checklist 1.)

## 3. Audiological Assessment of Severe and Profound Hearing Loss

**Table:** 

The hearing assessment is for the purposes of: • Understanding communication needs and the impact of hearing loss • Nonauditory needs assessment • Diagnostic assessment with behavioral testing • Diagnostic assessment with objective testing • Developing a comprehensive treatment plan

### 3.1 Understanding Communication Needs and the Impact of Hearing Loss

It is essential to examine the self-reported communication difficulties experienced by the client and, from these, to develop individualized treatment goals. Assessment should cover the client's hearing-related activity limitations and participation restrictions, their hearing and communication needs at home, at work and/or in education; as well as any psychosocial difficulties related to hearing. In addition, since family support is very important for people with severe and profound hearing loss, it is important to understand the experiences of their significant others. Table 2 (in Appendix 3: Toolkits) contains appropriate measures for both the client and the communication partner.

All this information can be used by the HCP together with the client and their families to structure a treatment plan.

Recommendations (see evidence Appendix 1: Assessment, 3; Section 3.1)

1. Prior to the hearing assessment, enquire if communication support (e.g., interpreters, speech-to-text) or adjustments are required to ensure clinical care is accessible. See Sections 1 and 2.
2. The communications needs analysis could be completed using open-ended questions and one or more self-report instruments. Some instruments measure hearing and communication difficulties, for example, the Revised Hearing Handicap Inventory (Cassarly et al, 2020) and The Speech Spatial and Qualities Questionnaire (Gatehouse & Noble, 2004) at a particular point in time and, in so doing, set a baseline from which outcomes can then be measured as part of ongoing care and support (see Section 5). Other measures include client-specific goal setting procedures such as The Client Oriented Scale of Improvement (Dillon et al, 1997) or The Glasgow Hearing Aid Benefit Profile (Gatehouse, 1999). See Table 2 in Appendix 3: Toolkits.
3. A full social history of the client's circumstances should include living arrangements, employment, social interactions, and hobbies and give the professional an overview of what their life looks like on a daily basis, particularly around levels of social isolation/interactions. This will allow a deep understanding of the broader potential impact of hearing loss.
4. The impact of the hearing loss on close friends and family (third-party disability) should be measured, such as The Hearing Impairment Impact-Significant Other Profile (Preminger & Meeks, 2012) or The Significant Other Scale for Hearing Disability (Scarinci et al, 2009). See Table 2 in Appendix 3: Toolkits.
5. Shared goal settings involving both client and close friends and family are recommended when possible, such as The Family Oriented Communication Assessment and Solutions (Crowhen & Turnbull, 2018) or Goal Sharing for Partners (Preminger & Lind, 2012). See Table 2 in Appendix 3: Toolkits.

(See Appendix 3: Toolkit Table 2 .)

### 3.2 Nonauditory Needs Assessment

Alongside the auditory assessment, it is essential to examine factors (outside of the hearing loss) which also influence the client and the possible treatment options. These nonauditory issues may influence the need for modification in testing, additional counseling, and referrals to other professionals and may change the treatment options to be offered.

Recommendations (see evidence Appendix 1: Assessment, 3; Section 3.2)

1. Information should be gathered on the following comorbidities and other relevant factors: See Table 3 (in Appendix 3: Toolkits)
  - Cognitive ability.
  - Mental health status.
  - Physical status (mobility and craniofacial status).
  - General health.
  - Dexterity.
  - Visual status.
2. Clients presenting significant neurological disorders/cognitive impairment may require an adapted assessment test battery. Tests that are assessed verbally must be administered carefully to avoid confusing hearing and cognitive aspects.
3. HCPs with training may perform additional tests outside the scope of audiology (e.g., tests of dexterity, vision, cognition, and depression) or make recommendations for an onward referral for completion of these tests if required.
4. HCPs should make appropriate referrals for onward management within and/or outside the health system, where significant nonauditory needs are discovered requiring further support.

(See Appendix 3: Toolkit: Table 3 .)

### 3.3 Diagnostic Assessment with Behavioral Testing

Clients with severe and profound hearing loss should receive an individually tailored audiological assessment which should include a comprehensive audiological examination including case history, otoscopy, and behavioral and physiological auditory measures. The key elements of the audiological assessment include, but are not limited to, the following:

- Otoscopic examination.
- Measurement of hearing impairment (type and extent of hearing loss).
- Assessment of the need for additional evaluation and/or medical referral.
- Assessment of candidacy for amplification, referral for implantable hearing devices and for other treatments.

Recommendations (see evidence Appendix 1: Assessment, 3; Section 3.3)

1. It is beneficial to take an extensive, chronological ontological history, taking the client back to the start of their hearing problems to fully understand their journey so far and enable problem solving to take place when developing a treatment plan later in the assessment. Often the client will be returning for a reassessment of their hearing, rather than attending for a first assessment and, in such cases, the medical history should focus on any changes in hearing or tinnitus since their last assessment.
2. In the case of sudden onset of severe and profound hearing loss or acute tinnitus, the HCP must refer the client for a medical consultation with an Otolaryngologist. This should be treated as a medical emergency, and the client should be seen urgently.
3. The measurement of the degree and type of hearing loss should include both threshold and uncomfortable loudness levels to ascertain the dynamic range for both ears.
4. Speech recognition testing is beneficial in considering amplification strategies, setting expectations, and onward referral for cochlear implants. See Section 4.6.
5. The HCP and the client should consider what they want to measure (e.g., evaluating amplification or considering a cochlear implant assessment). Speech testing can be a useful qualitative measure of both communication abilities and hearing aid benefit.
6. Speech testing may be dictated by local/national protocols for cochlear implant referrals, but ideally, it should be flexible enough to assess auditory speech perception, auditory-visual speech perception, and conversational fluency either through one test or through a battery of tests available to the HCP.
7. Speech in noise testing or tests of spectro-temporal modulation (STM) detection can inform if there is a particular need for additional hearing aid features or remote microphones to give some advantage in noisy environments.
8. Cochlear dead region testing might be undertaken to guide expectations, to inform amplification strategy or in assessing candidacy for cochlear implants.

(See Appendix 3: Toolkit: Table 4 .)

### 3.4 Diagnostic Assessment with Objective Testing

People with severe and profound hearing loss should receive individually tailored audiological assessments that include objective measures to identify the pathophysiological bases of their hearing loss. Objective measures also may be needed to estimate hearing thresholds for clients who are unable to participate in behavioral assessments. These measures may include the following:

- Tympanometry.
- Otoacoustic emissions.
- Electrocochleography.
- The auditory steady-state response or auditory brainstem response.
- The cortical N1-P2 response.

Recommendations (see evidence Appendix 1: Assessment, 3; Section 3.4)

1. Tympanometry should be performed with any indication of otitis media with effusion (OME) or other middle ear problems. Undetected OME can lead to complications such as serous labyrinthitis and also may lead to difficulties during CI surgeries.
2. Otoacoustic emissions can be present with severe-to-profound losses arising from auditory neuropathy and thus should be measured when the pathophysiology underlying the hearing loss is unknown. While the presence of OAEs with severe-to-profound loss is consistent with auditory neuropathy, the absence of OAEs does not rule it out.
3. In cases of possible auditory neuropathy, electrocochleographic measures (ECochG) may be conducted to confirm the presence of auditory neuropathy and to help distinguish between pre- and postsynaptic dysfunction. This may impact treatment recommendations and planning.
4. If behavioral thresholds cannot be reliably measured (e.g., due to severe cognitive impairment), thresholds should be estimated using objective methods. The auditory steady-state response (ASSR) can be used to estimate thresholds in the severe-to-profound range and is more robust and accurate than the auditory brainstem response (ABR) at these hearing levels. It is thus recommended that the ASSR be used instead of (or in addition to) the ABR. If the client can sit quietly for testing, more precise threshold estimates can be obtained by measuring tone-evoked N1-P2 responses.

(See Appendix 3: Toolkit: Table 5 .)

### 3.5 Developing a Comprehensive Treatment Plan

The final part of the assessment is to use all the information gathered to counsel the client and their communication partner on the results of the assessment and undertake joint decision-making to complete the treatment plan.

Planning is based on the analysis of:

- The behavioral and/or objective diagnostic testing results, and the impact the hearing loss might have on communication.
- Any relevant audiological and nonaudiological history which may influence any treatment option plus assessment of current communication strategies.
- Priorities from the goals set in the needs analysis.

Options for managing their hearing needs should be discussed, outlining the potential benefits and limitations of each option. All options should promote independence and encourage self-management. This understanding is established through a process of counseling, information sharing, education, and discussion.

- Commonly, this will include the fitting of hearing aids as part of the treatment plan. See Section 4.1.
- Counseling and rehabilitative support is essential. See Section 5.
- Communication tactics, speech reading and advice on making the most of their hearing is vital for people with severe and profound hearing loss and should not be overlooked. It is particularly important to link this information back to their needs analysis. See Section 5.
- Assistive listening devices that work on their own and/or with hearing aids should be routinely discussed, linking back to their needs analysis. Important aspects are consideration of remote microphone systems to improve communication in adverse situations and a practical demonstration of any appropriate device offered. See Section 4.4.
- Support in managing tinnitus should be arranged if required. See Section 5.2.
- Referral for assessment for cochlear implant or other implantable hearing devices if appropriate. See Section 4.6.
- Signposting/referring to other organizations and support groups for people with hearing loss. Referral for medical or surgical treatments, if these might be suitable.
- These options are then recorded in a treatment plan.

Recommendations (see evidence Appendix 1: Assessment, 3; Section 3.5)

1. HCPs should conduct all the procedures outlined in a person-centered manner, which is linguistically and culturally sensitive.
2. HCPs should recognize that the client is an expert about the impact of their hearing loss.
3. Amplification discussions should be transparent about what the client can expect from a change in any amplification. See Section 4.1.
4. Treatment options should always consider options wider than amplification. Technology is always improving but on its own is generally not enough to overcome the issues the person with severe or profound hearing loss is experiencing. See Sections 4.2 to 4.5 and 5.
5. The conversations about cochlear implants need to be considered in terms of an opportunity for the client to explore another intervention which would improve auditory outcomes. See Section 4.6.
6. All treatment options should be agreed and recorded in a personalized care plan, considering the client's preferences, including goals, and giving the client a copy. See Table 6 (in Appendix 3: Toolkit). This plan should be initially based on information gathered at the assessment phase and is determined in conjunction with the client and their communication partner. It should be flexible and updated on an ongoing basis.

(See Appendix 3: Toolkit; Table 6 .)

(See Appendix 4: Clinical Checklist 1.)

## 4. Technology to Support Severe and Profound Hearing Loss

### 4.1 Prescribing and Fitting Hearing Aids

**Table:** 

When selecting technology, the following considerations should be made: • Compression • Programs • Frequency compression and transposition • Prescriptions and verification • Selecting technology for unilateral severe and profound hearing loss • Temporary threshold shift and maximum power output

Severe and profound hearing loss impacts communication in several ways, including: inadequate speech audibility; loudness sensitivity as a result of a small dynamic range; and susceptibility to background noise. In addition to threshold elevation, clients with severe and profound hearing loss are likely to experience poor frequency selectivity and loss of auditory input due to cochlear dead regions, which are characterized by a complete loss of function of inner hair cells in a certain region of the cochlea (Moore, 2001). Accordingly, these clients require hearing aids that achieve the following goals:

- Improve speech audibility while avoiding loudness discomfort.
- Provide acceptable speech quality.
- Preserve or enhance useful acoustic cues.
- Improve signal-to-noise ratio, ease of listening, and listening comfort in background noise.
- Support best possible communication via cell or landline telephones.
- Have convenient and reliable connections to hearing assistive technology.
- Limit maximum output to avoid further hearing damage.

While there is a large body of evidence for clients with mild and moderate hearing loss, there is substantially less evidence that supports clinical decision-making when selecting and fitting hearing aids for clients with severe and profound hearing loss. The sections that follow provide an update to the earlier Guidelines (Turton et al 2020), reviewing clinical evidence from 2020 to 2025. Recommendations for technology, selection and fitting procedures for clients with severe and profound hearing loss are based on current best practice, relevant studies, and industry white papers.

(See Appendix 3: Toolkit; Table 7 .)

#### 4.1.1 Compression

Clients with severe and profound hearing loss should be fitted with compression parameters which result in improved speech audibility and avoid distortion of usable speech cues. Output limiting should be appropriately set to avoid loudness discomfort or auditory damage due to over-amplification.

Recommendations (see evidence Appendix 1: Technology, 4; Sections 4.1: Prescribing and Fitting Hearing Aids and 4.1.1: Compression)

1. Clients with severe and profound hearing loss should be fit using multichannel wide-dynamic range compression (WDRC) rather than linear amplification.
2. Use the lowest compression ratio that provides acceptable speech audibility. To maximize intelligibility of conversational speech while preventing loudness discomfort, low input levels may not be fully audible to some clients. It is not recommended to use high compression ratios to compress the entire speech range into the client's dynamic range.
3. Number of bands should be sufficient to adjust frequency-gain characteristics for the client's audiogram and to provide suitable noise reduction and feedback management. There is limited evidence that a very large number of narrow-band channels coupled with high compression ratios may reduce vowel perception. There is a lack of evidence regarding whether a larger number of bands or channels will affect the benefits of digital noise reduction or feedback reduction for clients with severe and profound loss.
4. Slow- to moderate-rate WDRC or adaptive WDRC results in best outcomes for many clients with severe and profound hearing loss. Accordingly, fast-acting WDRC should be used as a deliberate choice when it is expected that benefits (i.e., audibility of low-intensity speech sounds) will outweigh the drawbacks (i.e., modification of the speech envelope) for that client. Fast-acting WDRC amplification also may be an appropriate choice if other aspects of the aid (such as assistive device compatibility) are necessary.
5. Extra care may be needed when transitioning clients who are accustomed to linear amplification to multichannel WDRC. In addition to counseling regarding expected differences (e.g., lower overall loudness) and benefits, some clients may benefit from a stepped adjustment period in which frequency-gain response is adjusted and trialed before increasing compression strength.
6. Speech enhancement algorithms may improve recognition of low-intensity speech and reduce listening effort (data for Mandarin, no data currently available for nontonal languages).

#### 4.1.2 Device Choices and Programs

Clients with severe and profound hearing loss should be fitted with listening programs that maximize available speech information. Careful attention should be paid to providing noise management, including digital noise reduction, adaptive directionality, and a program that facilitates convenient use of a remote microphone.

Recommendations (see evidence Appendix 1: Technology, 4; Sections 4.1: Prescribing and Fitting Hearing Aids and 4.1.2: Device Choices and Programs)

1. Bilateral fittings are recommended whenever feasible (see below for recommendations in cases of asymmetric hearing loss with little usable hearing in one ear).
2. Automatic/adaptive directionality should be used to improve the signal-to-noise ratio when the signal and noise are spatially distinct.
3. Binaural directionality should be used when possible, although the benefits over monaural directionality may be limited to specific listening environments.
4. Digital noise reduction (denoising) can improve listening comfort and reduce listening effort.
5. To encourage the use of remote microphones in situations where directional benefit is inadequate, hearing aids should offer convenient remote access. This may be in the form of an automatic program that activates when the remote microphone is active, or a manual program in which the listener selects remote microphone input.
6. Feedback should be controlled via active digital feedback management. Passive feedback management (i.e., within-band gain reduction) may limit speech audibility or adversely restrict the amplified speech range for some clients. Active feedback management should be engaged and its function verified.
7. Custom earmolds with appropriate venting should be used rather than open or closed domes, as custom molds allow for maximum gain, maintain a consistent position and direction of sound bore or hearing aid receiver, and minimize potential for acoustic feedback.
8. Binaural (“ear to ear”) wireless streaming via telecoil or Bluetooth input can improve phone communication compared to monaural telephone listening.
9. Prototype aids with enhanced motion sensors show promise for improved directional benefit in active environments.

#### 4.1.3 Frequency Lowering for Adults with Severe and Profound Hearing Loss

Frequency lowering should be used in cases where the resulting improvements in high-frequency sound audibility result in better speech recognition than with traditional frequency-gain processing alone. After parameter adjustment, frequency lowering should be validated objectively and subjectively.

Recommendations (see evidence Appendix 1: Technology, 4; Sections 4.1: Prescribing and Fitting Hearing Aids and 4.1.3 Frequency Lowering for Clients with Severe and Profound Hearing Loss)

1. For clients with severe and profound loss, hearing aids should initially be fit with frequency lowering turned off, then engaged as needed to improve high-frequency speech perception.
2. If frequency lowering is used, there is limited evidence suggesting that frequency compression may result in better outcomes than frequency transposition for clients with severe and profound hearing loss.
3. Frequency lowering should be fit using a validated procedure, with the minimum strength necessary to provide adequate audibility.
4. Current evidence does not show that individual spectral resolution is related to frequency compression benefit.

(See Appendix 3: Toolkit; Table 8 .)

#### 4.1.4 Prescriptions and Verification

Hearing aids for clients with severe and profound hearing loss should be fitted using REM ideally measured with a broadband speech or speech-like signal and a validated prescriptive target as the starting point for adjustments. After adjustment, REM should again be performed to evaluate audibility.

Recommendations (see evidence Appendix 1: Technology, 4; Sections 4.1: Prescribing and Fitting Hearing Aids and 4.1.4: Prescriptions and Verification)

1. A validated prescriptive procedure should be used to guide frequency-gain settings.
2. For clients with moderately severe to severe loss, the DSL v 5.0 prescription is likely to result in greater aided speech audibility for conversational-level speech inputs compared to the NAL-NL2 prescription.
3. Probe-microphone or coupler measurements should be used to assess signal audibility.
4. When hearing aid parameters are adjusted according to subjective judgments, every attempt should be made to maintain a level of speech audibility which supports aided speech recognition. A period of acclimatization or counseling may be useful, especially when transitioning users to higher prescribed gain. Probe-microphone or coupler measurements should be used to confirm adequate signal audibility following adjustments.
5. Input signals for real-ear or coupler measures should represent the signal of interest (usually speech). Static noise or tone signals may result in aid behavior that is atypical for speech amplification.
6. Gain should be increased in cases of mixed hearing loss, usually by inputting bone-conduction thresholds when calculating prescribed aided output.
7. When dead regions are confirmed or suspected, gain may be provided in the frequency range of the dead region unless the client reports poor speech quality or loudness discomfort.

(See Appendix 3: Toolkit; Table 9 .)

#### 4.1.5 Selecting Technology for Asymmetrical Severe and Profound Loss

When hearing loss is in the severe and profound range in only one ear, the level of hearing in the other ear should be taken into account in hearing aid fitting decisions. Unilateral, bilateral, CROS (Contralateral Routing of Offside Signal) or BiCROS hearing aid fittings may be appropriate, depending on hearing thresholds, speech recognition, dynamic range, and the client's communication goals. In this section, we review recent evidence related to use of BiCROS amplification.

Recommendations (see evidence Appendix 1: Technology, 4; Sections 4.1: Prescribing and Fitting Hearing Aids and 4.1.5: Selecting Technology for Asymmetrical Severe and Profound Loss)

1. BiCROS amplification should be considered in cases of asymmetric hearing loss where one ear is unlikely to receive sufficient aided audibility. Because improved speech in noise with BiCROS versus monaural amplification will be affected by the specific environment, it is recommended that each client be encouraged to trial hearing aids with and without the transmitter in their everyday communication settings.
2. Real ear response should be measured with and without the BiCROS transmitter and the transmitter response adjusted so that the intended frequency-gain response is maintained on the better ear. Selection of a BiCROS transmitter with adequate technology level and range of adjustments will facilitate adjustment of the transmitter response.
3. To maximize recognition of speech in noise and perceived sound quality, both the receiver and transmitter components should be configured to have adaptive directionality. A convenient means of disabling the transmitter microphone, such as a volume control or on-off switch, may be useful in cases where the primary signal of interest is on the receiver side and the primary noise source on the transmitter side. When such features are included, the client should also be trained on their use.
4. An experimental BiCROS system with transcranial routing from an in-the-canal aid demonstrated similar objective and subjective speech recognition to a conventional (wireless) BiCROS.

#### 4.1.6 Maximum Power Output and Threshold Shift

Hearing aid gain and maximum output should be constrained to prevent damaging sound levels. Due to the high sound levels produced by their hearing aids, clients with severe and profound hearing loss are at risk for temporary and permanent threshold shift.

Recommendations (see evidence Appendix 1: Technology, 4; Sections 4.1: Prescribing and Fitting Hearing Aids and 4.1.6: Maximum Power Output and Threshold Shift)

1. Maximum output levels should be measured as real-ear sensation levels using narrow-band signals.
2. Compression limiting (rather than peak clipping) should be used to avoid distortion while limiting signal output.
3. Use aids with frequency-specific adjustments for maximum output. The ability to adjust maximum output in specific bands will allow for a careful balance of output control without unnecessary headroom reduction.
4. Disabling the ability to increase manual volume controls above desired levels should be considered when the client is at risk of permanent threshold shift due to sound levels and unable to conservatively adjust volume (e.g., has poor dexterity or cognitive limitations).

(See Appendix 3: Toolkit; Table 10 .)

#### 4.1.7 Considerations for Music

**Table:** 

Music may be an important part of an individual's life, and so the topic of music should ideally be discussed with each individual with severe and profound hearing loss. • Limited research in this area specifically directed toward individuals with severe and profound hearing loss. • Hearing aids in general improve the enjoyment of music for individuals with hearing loss; however, when looking at specific characteristics of music, the results vary greatly. • The current consensus about hearing aid technology is to do the least amount of processing to the signal as possible, as these features were designed for comfort and speech understanding in quiet and in noise, not music enjoyment. • Hearing aids can be adjusted to accommodate music, but this may involve a certain amount of trial and error with adjustments to the hearing aids, as HCPs often report that they have limited experience with music. • A/B comparisons with familiar sound samples or with musical excerpts played with the individual's instrument in the clinic (if possible). This is important as the acoustic properties of the music cannot be generalized simply by using labels for style or genre. • Modifying the hearing aid systematically with minimal changes per appointment and potentially with the person's musical instrument and/or the use of music files can create settings that are suitable for the individual's goals. • A modified listening program can be made, and potentially more programs can be used in the case of multi-memory hearing aids to suit specific goals such as a single program for performing live music and another program for listening in the audience or recordings at home.

Perceiving and understanding speech in different environments may not be the only priority for the individual client; they may expect more from their devices. Depending upon the individual, he/she may have other goals or auditory needs that may be discovered using a needs assessment such as that obtained using a COSI (Dillon et al, 1997) or other measurement tool, or perhaps from a case history or even a conversation with a HCP. These goals may include the ability to play or listen to music at live events or via recordings. Listening to and playing music has many benefits that extend into general health and well-being. Music can be used therapeutically for a variety of health conditions, including somatic (e.g., pain management), psychiatric conditions (e.g., insomnia), and general well-being (e.g., stress reduction; Jespersen et al, 2022). Given these and other potential benefits, naturally including pure enjoyment, music should ideally be discussed with each individual with severe and profound hearing loss regardless of how they relate to it—as casual listeners or even professional musicians. However, the topic of music is not usually covered by clinicians, as pointed out by Greasley et al (2020), who found that in a survey of HCPs and hearing aid wearers, music was not discussed in more than half of the cases that they analyzed.

When offering guidance for music, this greatly depends on the relationship that the individual has with music and what goals he/she has when listening to or playing music. These goals may change depending upon the situation. For instance, is the person playing an instrument, or is he/she in the audience listening (or both, in the case of performing in an ensemble), is the music being listened to in the foreground or is it purely in the background, or something in between, and is the individual participating in a conversation such as in a live jazz club, where their attention may alternate between music and speech? Furthermore, does the individual need to wear a hearing aid to hear a conductor or teacher so as not to exclude them from participating in a musical ensemble (Vaisberg et al, 2018)? In addition, each individual might have different requirements or preferences for whatever he or she is listening to. Hearing aid technology varies between manufacturers, and when individual taste, individual pathology, and potential distortion within the auditory system are all factored in, making strong recommendations within this guideline document is difficult. This does not in any way diminish the importance of discussing music with the individual with a hearing loss and potentially modifying the hearing aid to suit their needs as a musician or music enthusiast.

At the time of writing, there are a limited number of studies for individuals with a severe and profound hearing loss that focus on the perception of music via hearing aids. Most of the studies on music focus on lesser degrees of hearing loss, and so it is difficult to make general recommendations regarding the fitting of hearing aids to individuals with severe and profound hearing loss who want to hear music better. Hearing aids in general improve the enjoyment of music by individuals with hearing loss (Chern et al, 2022); however, when looking at specific characteristics of music, the results vary greatly (Greasley et al, 2020, 2026; Madsen & Moore, 2014; Swann et al, 2024; Vaisberg et al, 2018). Poorer ratings of music appreciation are given by those individuals with a severe or greater hearing loss when compared to individuals with a mild to moderate hearing loss (Looi et al, 2018). The current consensus about hearing aid technology available at the time of writing is to do the least amount of processing to the signal as possible, especially because there may already be some distortion within the auditory system caused by the hearing loss itself (Moore, 2016, 2022). This results in a need to turn off or judiciously use automatic features like frequency lowering, feedback management (if possible), noise reduction, and adaptive beamforming, as these systems have been designed specifically to address issues of comfort and speech understanding in quiet and noise and not music (Moore, 2016; Uys et al, 2016; Chasin, 2022; Hake et al, 2025). The amplification can be adjusted to the individual's preference while still maintaining the compression characteristics that are necessary to compensate for the hearing loss and provide audibility for speech (Vaisberg et al, 2021; Chasin, 2022; Hake et al, 2025). A good example of a fine-tuning option is to increase the low-frequency gain as suggested by Madsen and Moore (2014) and Vaisberg et al (2021).

Trying to translate what the individual says about music into parameter adjustments for the hearing aid may involve a certain amount of trial and error, as HCPs often report that they have limited experience with music (Greasley et al, 2020; Swann et al, 2024). This can potentially be made easier by using A/B comparisons with familiar sound samples or with musical excerpts played with the individual's instrument in the clinic (if possible) which is important as the acoustic properties of the music cannot be generalized simply by using labels for style or genre (Lesimple et al, 2024). The need for the HCP to understand and translate the user's needs into parameter changes is paramount. Modifying the hearing aid systematically with minimal changes per appointment and potentially with the person's musical instrument and/or the use of familiar music files can create settings that are suitable for the individual's goals. A modified program can be made, and potentially more programs can be generated in the case of multi-memory hearing aids to suit specific goals such as a single program for performing live music and another potential program for listening in the audience or to recordings at home (Chasin, 2022).

Recommendations (see evidence Appendix 1: Technology, 4; Sections 4.1: Prescribing and Fitting Hearing Aids and 4.1.7: Music Options)

1. Add a dedicated listening program (or programs) for music in multimemory hearing aids to address the listening goals of the client.
2. Compression should be set similarly to what is used in the speech program(s) to address the hearing loss and be adjusted to the individual's preference; however, the gain and MPO should be verified by REM to ensure that any amplification changes do not cause discomfort.
3. The frequency response should be adjusted to the client's preference, but a first step could involve an increase in the low frequency gain.
4. Noise reduction, including wind noise or transient noise management, should be turned off or reduced if possible.
5. Ensure that adaptive directionality (beamforming) is off or reduced if possible. Use a fixed (nonadapting) microphone setting which is either directional or omnidirectional.
6. Adaptive feedback management should be off if possible, and if needed, the gain should be reduced to reduce feedback. However, given the gain requirements that are needed for the client with severe and profound hearing loss, this may not be possible.
7. Frequency lowering should be off or reduced in strength if needed for audibility. This is dependent upon the implementation by the hearing aid manufacturer.

The discussion and recommendations regarding options for music in this section have applied so far to hearing aids only; however, there are other options for individuals with severe and profound hearing loss that could be pursued together with the HCP depending on the decisions that have been made regarding the use of hearing aids and/or cochlear implants, or involve no technology at all. Remote microphone technology, which is discussed in Section 4.2, may be the first option to be considered. Three additional options are as follows.

First, sound as we know it is a physical phenomenon that, depending on the spectral content, can be perceived through the sense of touch. This method of perception can be further enhanced using vibrotactile devices to transmit or even transpose music to vibrations that can be felt rather than heard. These devices can be used by themselves or in conjunction with hearing aids and/or cochlear implants (CI). These devices are not widely used clinically at the time of writing; however, research has shown promise in this area (Fletcher, 2021; Hopkins et al, 2016; Hopkins et al, 2023),

Second, a bimodal fitting, where a CI is fitted on one ear, and a hearing aid is in the other, is an option to enhance electrical hearing with acoustic hearing and vice versa. Extending the amplified bandwidth for the individual by using a hearing aid in conjunction with a cochlear implant improves the perceived sound quality for music over using the CI alone (D'Onofrio & Gifford, 2021). Bimodal fittings are discussed in Section 4.6.4 in this guideline, and the authors also suggest that the reader consult specific practice guidelines on bimodal fittings (Holder et al, 2022).

Third, while this may be considered by some to be an unorthodox approach in a guideline document for HCPs, it is possible to compose music or even perform music that accommodates an individual's hearing loss; in other words, adapt the music to the individual. This has been demonstrated by composers (e.g., Chapman, 2023; Hugill, 2023) who have composed and performed/organized concerts with special considerations for individuals with varying degrees of sensorineural hearing loss, Meniere's disease, and other auditory disorders. The goal of this new music and concert performance is for the listener to enjoy and appreciate music in his/her own way so that the inability to hear or perceive certain sounds in a typical way is not a barrier. Recordings can also be modified using sound editing software to bring the music into the frequency and dynamic range that is audible and comfortable for the individual with a severe or profound hearing loss. The reader who is interested in these and other topics can consult research and experts in the field of musicology.

### 4.2 Prescribing and Fitting Remote Microphones

**Table:** 

When selecting remote microphone systems, the following should be considered: • The communication demands for the person with hearing loss • Connectivity with other devices of interest • Minimizing the number of components in the system • Implications of charging options and battery life for the user • Ability to interface with wireless technology in the community or workplace • Compatibility with emerging universal wireless technologies such as Auracast When verifying remote microphone systems, the following should be considered: • Equivalent Output to that produced by the personal technology when testing with equivalent input • Minimal additional circuit noise compared to that of personal technology • No additional distortion compared to that of personal technology • When tested in background noise, behavioral performance increases with use of remote microphone systems compared to that obtained with personal technology • Comfortable listening is maintained

#### 4.2.1 Considerations for Recommending and Managing Ongoing Use of Remote Microphone Systems

Hearing aids and/or cochlear implants are the most commonly fitted technologies for adults with severe and profound hearing loss. However, such devices do not meet all the communication needs of this population, and remote microphone technology can be used to improve performance, for example, when having conversations in noisier environments or when listening to a speaker at a distance.

Most of the research on such systems has been undertaken with children in classroom situations. The objective of this section is to summarize the evidence about recommending and managing the ongoing use of remote microphone systems for adults with severe and profound hearing loss.

Recommendations (see evidence Appendix 1: Technology, 4; Sections 4.2: Prescribing and Fitting Remote Microphones and 4.2.1: Considerations for Recommending and Managing Ongoing Use of Remote Microphone Systems)

1. Adults with severe and profound hearing loss can benefit from remote microphone systems in a range of situations and should be fully informed about them by HCPs. This should be reviewed proactively on an ongoing basis.
2. Communication partners of adults with severe and profound hearing loss experience Third Party Disability. This can be reduced when their partner makes use of remote microphone systems, and they should be fully informed about them by HCPs.
3. Having the opportunity to trial a remote microphone system is an essential part of decision-making for clients and communication partners.
4. HCPs need to set goals with clients and communication partners for the use of remote microphone systems.
5. Comprehensive instructions in a range of formats and ongoing education and support about remote microphone systems are needed for clients, communication partners and HCPs.
6. The complexity of remote microphone systems should be reduced for the benefit of clients, communication partners and HCPs.
7. Communication partners influence success with remote microphone systems and should be part of decision-making, fitting and ongoing management.
8. There is a need to increase community awareness of remote microphone systems.

(See Appendix 3: Toolkit; Table 12 .)

#### 4.2.2 Component Considerations

The various features and components of remote microphone systems are described in the American Academy of Audiology (AAA) Clinical Practice Guidelines: Remote Microphone Hearing Assistance Technologies (AAA, 2011). The AAA guidelines focus on hearing-assistive technology for individuals from birth to 21 years. Many of the available features and rationale for selection are included and applicable to the young adult population with severe and profound hearing loss. Use of remote microphone systems, and therefore the component choice, for adults depends heavily on the communication demands experienced by the client.

Given the complexity of options available across manufacturers, the selection of remote microphone systems is ideally considered at the same time as the selection of the personal device, hearing aids and/or cochlear implants (Thibodeau, 2020). The benefit from such systems is most likely to increase with the simplicity of the arrangement. The HCP should be aware that this can be impacted by the number of components that attach to a personal ear-level device, ranging from two components such as an audio shoe or receiver adapter for a cochlear implant and a wireless receiver to zero additional components such as a hearing aid/cochlear implant with wireless connectivity to a transmitter, smartphone, or an induction loop system. Another factor for simplicity that must be considered is the battery life and charging options. Some lifestyles that involve frequent travel make it difficult to work with multiple charging cords for transmitters/receivers.

Finally, the client's communication interactions at work, school, and the community must be considered to have optimal compatibility and maximum use of the features/components selected (Peters and Thibodeau, 2024). Ideally, if a user enjoys theatre, their personal remote microphone system could connect to the assistive technology provided at the performance hall. Similarly, a personal remote microphone system would connect to the conference microphone provided at work for group meetings if applicable for persons in employment settings. Some remote microphone systems allow for stereo audio, and may also have a feature for online video calls, allowing the microphone to pick up the talker's audio and send it to the other side. It is very likely that remote microphone systems provided in higher education could be the same as the client's personal system such that some components could be shared while enrolled in that program.

Recommendations (see evidence Appendix 1: Technology, 4; Sections 4.2: Prescribing and Fitting Remote Microphones and 4.2.2: Component Considerations)

When used correctly, the remote microphone system will provide benefit beyond that obtained with the local microphone system on the hearing aid and/or cochlear implant, depending on how challenging the daily listening demands are for everyone.

1. The system should provide wireless connectivity to components of interest to the client; examples include the client's smartphone, television, computer, tablet, vehicle audio signals, inductive loop microphones, etc.
2. The system should be comprised of the minimal number of components to facilitate troubleshooting and minimize repairs in bilateral/bimodal arrangements with consideration of the financial constraints for the client.
3. The charging options and battery life should meet the communication needs and lifestyle of the client.
4. The system should efficiently interface with other assistive technology that may be provided based on requirements in employment and/or higher education settings.
5. The system should be fit bilaterally unless contraindicated by atresia or severity of the hearing loss.

#### 4.2.3 Considerations Regarding Remote Microphone Verification

The use of remote microphone systems provides significant benefit for adults who use amplification and/or cochlear implants to compensate for all degrees of hearing loss. After selecting and fitting the remote microphone systems, the particular device should be verified with both electroacoustic and behavioral measures. The remote microphone system that delivers the signal via the personal hearing aid, such as an installed digital modulation (DM) receiver, can be evaluated using existing electroacoustic test equipment and couplers.

Three documents that relate to the verification of remote microphone systems include the ANSI S3.47 standard for “Specification of Hearing Assistance Devices/Systems” (ANSI, R2023), the American Academy of Audiology (AAA) Clinical Practice Guidelines: Remote Microphone Hearing Assistance Technologies (AAA, 2011), and the EUHA Wireless remote microphone systems—configuration, verification and measurement of individual benefit (EUHA, 2017).

The ANSI S3.47 standard includes recommended electroacoustic measurements that are like those recommended in the ANSI S3.22 standard for hearing aids, with specific requirements for placement of the transmitting microphone and the receiver. These procedures allow comparison across remote microphone systems because prescribed input levels and equipment arrangements are used (Thibodeau and Wallace, 2014; Qi and Thibodeau, 2023).

The Clinical Practice Guidelines on Remote Microphone Hearing Assistance Technologies (AAA, 2011) focus on hearing-assistive technology for individuals from birth to 21 years. There are specific protocols in these guidelines for the electroacoustic and behavioral evaluation of ear-level remote microphone systems when used with clients who wear hearing aids, cochlear implants, or who have hearing within normal limits. When fitting remote microphone systems, it is important that such electroacoustic verification be performed to ensure that the wireless signal is received by the listener at a level above that of the environmental signals processed through the hearing aid, resulting in a favorable signal-to-noise ratio (SNR). Research with these protocols suggests that variations exist in electroacoustic performance across remote microphone systems even when tested with the same personal hearing aid device and highlights the importance of electroacoustic verification (Salehi et al, 2018). In addition to electroacoustic verification, behavioral testing may also be performed. Typically, this is not necessary for adults with hearing aids because there is an abundance of research supporting the benefit that can be achieved. However, for remote microphone systems for use by clients with cochlear implants, behavioral evaluation is necessary. Protocols suggested in both the AAA (2011) and EUHA (2017) guidelines include comparison of speech recognition in noise performance when listening with the personal device alone (CI and/or HA) to that obtained when listening with the personal device connected with the remote microphone system. Benefits achieved with remote microphone technology over use of the personal device alone may be as great as 61% (Thibodeau, 2019a).

Following verification, the client and their communication partner will need instruction on the care and use of the chosen technology to realize the benefits of remote microphone systems in their real-world environments. In addition to the electroacoustic and behavioral verification in the clinical setting, the validation of the benefit depends on outcome measures following use of the remote microphone systems in everyday communication settings. A comprehensive tool to verify benefit across multiple communication activities is called the TELEGRAM (Thibodeau, 2004), which allows the rating of difficulty with and without the remote microphone systems for communication on the telephone, employment, entertainment, groups, recreation, and alarms. Ratings also are determined for the client's knowledge of Legislation relating to Assistive Technology and their Members of their family with whom they may frequently communicate.

Recommendations (see evidence Appendix 1: Technology, 4; Sections 4.2: Prescribing and Fitting Remote Microphones and 4.2.3: Considerations Regarding Remote Microphone Verification)

1. If a hearing aid is part of the remote microphone system, it should first be evaluated to ensure adequate function as described in this issue “Hearing aids: Prescriptions and verification.”
2. The output of the remote microphone system should not exceed that of the hearing aid.
3. The remote microphone system should not add significant additional circuit noise.
4. The remote microphone system should not cause an increase in distortion, as described in ANSI S3.47.
5. Electroacoustic verification should indicate that the frequency response of the personal hearing aid alone matches the frequency response when the hearing aid is coupled with a remote microphone within ± 5 dB.
6. As observed in the clinical setting, the client's behavioral performance with the remote microphone system should be significantly better than without it, as measured by the AAA, 2011 method (see the description in Appendix 3: Recommendation 6).

(See Appendix 3: Toolkit; Table 13 .)

(See Appendix 4: Clinical Checklist 3.)

### 4.3 Relevant Mobile Apps

Hearing-related mobile health applications, or apps, can be broadly divided into four categories: (1) screening and assessment, (2) intervention and rehabilitation, (3) education and information, and (4) assistive tools (Zitelli & Mormer, 2020). Some mobile apps contain functionality in multiple categories. For example, brand-specific apps may help with hearing aid control and fine-tuning, as well as have assistive functionality such as finding lost or misplaced devices.

Screening and assessment apps predominantly aim to provide an accurate insight into an individual's hearing ability, using either self-report questions or auditory signals such as tones or digits-in-noise. Some apps provide a qualitative rating of an individual's hearing and are designed predominantly as a hearing screening tool (Irace et al, 2021). Others provide a hearing threshold measurement or audiogram, and there is some evidence that a number of these apps can provide an accurate result when compared to standard audiometry (Oremule et al, 2024)

However, many studies validated hearing threshold measurements with individuals with no, mild, or moderate hearing thresholds or did not specify the participants' measured hearing thresholds. Therefore, it is unknown how accurate mobile apps are in assessing hearing for clients with severe and profound hearing loss.

Intervention and rehabilitation apps are predominantly those that either allow the use of a smartphone as a personal amplifier, support remote fitting and fine-tuning of hearing devices, or provide tinnitus relief. Apps that provide amplification are useful predominantly for individuals with mild degrees of hearing loss (Kang et al, 2025), and there is no evidence of their suitability for severe and profound hearing loss. Section 4.7 of this Guideline provides more information on the use of apps that support remote fitting and fine-tuning.

For clients with severe and profound hearing loss, the category of assistive apps may be of most relevance. This category includes apps with functionality such as real-time speech-to-text transcription, captioning for telephone calls, environmental sound alerts, and learning and using sign language.

Assistive apps also may be useful to help find accessible venues, for example, venues that are equipped with hearing loops. Other apps, such as Google Maps, can include information about the availability of hearing loops and assistive listening devices in venues. However, this information may be limited and not universally available as it is dependent on users to edit this information in the app. Google Maps also may be useful in finding times that specific venues are less busy, and therefore potentially more conducive to communication. Other apps are available that allow users to find acoustically friendly venues, such as restaurants and cafes with typically low levels of background noise.

Apps to support hearing and hearing loss are constantly launched and evolving, although their quality and relevance to a client's needs vary widely. A 2019 study (Romero et al, 2019), for example, found that of 21 apps which were either sign language translators, amplification/alerting assistants or speech-to-text apps, many were limited in their effectiveness. Some apps, particularly the sign language translator apps, were unusable or had been abandoned by the developer. Further research is required to assess the reliability and accuracy of mobile apps and to validate function with individuals with severe and profound hearing loss, before clear recommendations for their use can be given.

Recommendations (see evidence Appendix 1: Technology, 4; Section 4.3: Relevant Mobile Apps)

The HCP should:

1. Investigate the mobile apps which they feel could be suitable to recommend for their clients, taking into account the limited research evidence and validation information available for such apps.
2. Explore their client's digital literacy, and specifically their interest in, and ability to manage, mobile apps.
3. If suitable, provide the client with a list of relevant apps that could support their unique hearing, psychosocial and communication rehabilitation needs.
4. Remain up to date with mobile apps that can support their clients with severe and profound hearing loss.

Recommendations (see evidence Appendix 1: Technology, 4; Section 4.3: Relevant Mobile Apps)

(See Appendix 3: Toolkit; Table 14 .)

(See Appendix 4: Clinical Checklist 4.)

### 4.4 Alerting Devices and Other Assistive Listening Solutions

Hearing aids and cochlear implants have limitations for all listening situations, and other devices can be useful either through device streaming or as stand-alone products. The HCP needs to understand the client's most common or important communication situations (both near and far-field) to recommend appropriate systems to complement the hearing device, then ensure that the client has an opportunity to trial these systems and practice with those that suit their needs best.

Recommendations (see evidence Appendix 1: Technology, 4; Section 4.4: Alerting Devices and Other Assistive Listening Solutions)

The HCP should:

5. Explore the situations that matter most for their clients. This should include both current activities and places the client used to enjoy but stopped attending when their hearing deteriorated (e.g., the theatre, public meetings, social gatherings).
6. Maintain an up-to-date knowledge of the types of assistive listening solutions appropriate for each type of environment. This includes inductive loops, alerting devices, Bluetooth and Wi-Fi for acoustic information as well as text-based communication support systems (including captions, subtitles and surtitles for live and recorded performances as well as personal communication systems), especially recent developments using cell/mobile phone technology at minimal or no cost.
7. Unless contraindicated, the t-coil should be activated where fitted so the client can experience a good working inductive loop, as this remains the most widespread and effective way to hear well in public spaces.
8. Provide the client with the opportunity to try any potentially helpful assistive listening devices, ideally on location (e.g., their own home, a social club, etc.).
9. If the clinic cannot provide regularly updated assistive listening equipment and/or advice, build relationships with other local providers who can fulfil this requirement.
10. Remain up to date with any local authority provision or other sources of financial assistance in purchasing devices.
11. Where available, give information on assistance animals and encouragement to explore their eligibility if interested.

(See Appendix 3: Toolkit; Table 15 .)

(See Appendix 4: Clinical Checklist 5.)

### 4.5 Public Broadcast Protocols (Auracast)

Accessibility of sound in public spaces remains difficult for people with severe and profound hearing loss, even with a typical public address (PA) system. Current solutions exist to circumvent this problem, such as the use of a telecoil-based loop system, which is existing technology that continues to work well, with installation being mandated in most public spaces today across multiple countries. However, the installation of loop systems can typically be costly, and may need to be planned prior to the actual build, so access is often limited to preinstalled sites.

Wireless microphone technology can work very well in these situations, being able to digitally transmit the speaker's voice to the hearing devices or directly stream the output of the Public Address (PA) system. However, the use of this system requires either the additional step to connect to the PA system or the main speaker, which can be cumbersome and raise concerns around stigma.

Another alternative is to look to newer ways of broadcasting—including the growing awareness of Auracast technology. This is a broadcasting technology developed by the Bluetooth Special Interest Group (SIG), developed to work on the Bluetooth Low Energy Audio codec introduced in 2021. The authors acknowledge the existence of other wireless broadcasting protocols (e.g., audio over Wi-Fi, other proprietary protocols on 2.4 GHz); however, these have not been included in this section due to either the adoption of technology into hearing devices at the current time seeming unlikely, or the topic has already been dealt with in prior sections.

From a technical standpoint, Auracast has many benefits to offer over and above telecoil loop systems. Compared to telecoil systems, digitally modulated signals can provide superior audio quality in stereo, allowing people to have a greater appreciation for the signal being delivered. The ease of installation makes Auracast a very viable option for the future, only having to install a transmitter at the back of the PA system, although for the near future sites should ideally support a dual broadcasting option (both telecoil and Auracast).

However, the wider use of Auracast systems relies heavily on the availability and consequent adoption of transmitters and receivers by the market. Currently, there are only a handful of hearing devices that are compatible with Auracast, of which very few are targeted toward the severe and profound population. Bluetooth SIG estimates that in 2030 there will be 3 billion devices and 2.5 million sites supporting Auracast broadcasting. While Auracast has promises of accessibility for the future, the technology is not widely adopted in the market today, and so the use of currently available technologies is still required.

At the time of writing this guideline, there is no peer-reviewed literature looking into the use of Auracast solutions for the severe and profound population. The IEC is working toward standardizing the use of such broadcasting technologies for hearing aids, with standards estimated to be completed in late 2027. Until then, there is wide variability in how each manufacturer of transmitters and receivers implements their broadcast signal and risks to consistency across Auracast-compatible sites.

Recommendations (see evidence Appendix 1: Technology, 4; Section 4.5: Auracast)

1. For the foreseeable future, there will be a transition period where both telecoil and Auracast will continue to co-exist in future public space installations.
2. HCPs should actively consider whether their client's needs for the near future necessitate the use of an Auracast-enabled hearing device, or whether their needs can be met with current technologies such as telecoil or other wireless technology.
3. HCPs should continue to opt for technologies within hearing aids that provide clients with the best accessibility in public spaces, such as the more widely available telecoil loop systems and/or wireless microphone technologies.

(See Appendix 3: Toolkit; Table 16 .)

(See Appendix 4: Clinical Checklist 6.)

### 4.6 Cochlear Implant Candidacy and Bimodal Hearing

**Table:** 

Globally, the criteria for cochlear implants (CI) vary, and uptake for adults can be low for a variety of reasons. HCPs should: • Have a level of awareness of cochlear implantation • Explain the clinical effectiveness • Understand the associated costs with CIs • From this information, the HCP should feel comfortable starting the conversation about CI with candidates. Equally, hearing health professionals should ▪ Understand the impact of bimodal options ▪ Understand the limitations of other implantable devices for this population Research indicates that suitable candidates can expect significant, life-changing benefits following implantation. These benefits cannot be achieved using hearing aid technology alone. This chapter aims to provide education and counseling to our clients on the range of available hearing technologies. This helps them to understand that hearing aids do not have to be the final step in their hearing journey. Conventionally, aided acoustic hearing alone may not achieve high levels of speech understanding. Remote fittings for CI recipients are possible with all CI manufacturers (with and without a facilitator). Several mobile applications offer supplementary features beyond the conventional options of fine-tuning parameters such as volume and sensitivity. These include the functionality for live face-to-face fittings or asynchronous upload of new maps. Moreover, these applications offer audiological assessments, encompassing the measurement of hearing thresholds, the evaluation of speech perception in both quiet and noisy environments, and the provision of auditory training options. The extent to which these functions may supplant conventional clinic visits remains a subject to be explored in forthcoming research endeavors. In addition, a comprehensive evaluation is imperative to ascertain the evidence supporting these functions, their practical benefits for clients and HCPs, and the extent of their acceptance within the relevant communities

#### 4.6.1 Level of Awareness of Cochlear Implantation

In all nations, the proportion of adults with severe or profound hearing loss who have access to cochlear implantation is minimal. On a global scale, it is estimated that only one in twenty individuals who are eligible for cochlear implantation possesses a CI. The WHO World Report on Hearing 2021 ( www.who.int/publications/i/item/world-report-on-hearing ) has provided an extensive overview and recommendations about the global hearing and care policies across the lifespan. As a key insight, the report states that severe and profound hearing loss can have a significant negative impact on both children and adults. It is further stated that CIs are now widely accepted as a viable intervention for severe and profound hearing loss in both children and adults and have been proven to be both effective and cost-effective. As a key component, it is vital that clients receive rehabilitation to ensure they receive the maximum benefit from their CI.

There are a number of professional or client-driven initiatives that promote internationally recognized standards of care for cochlear implantation. These standards inform clients and healthcare practitioners of when specialist referrals and treatment options should be considered. An example of international standards of care can be found at https://www.hearring.com/hearring-quality-standards/ , CIICA-Consensus-Statement.pdf or Living Guidelines | Adult Hearing.

The provision of CIs is subject to variation according to the regulatory framework and financial coverage policies that apply in each country. It is therefore important to familiarize oneself with the national regulations that apply prior to initiating a conversation about CIs to ensure the right referral pathways (see Table 2 ).

**Table 2:** Example of country specific/national guidelines

Country	Organization	Reference
Overview of many countries	CI task force	https://adulthearing.com/resources/#regional
France	French ENT society	RCP_SFORL_Indications_implant_cochleaire_adulte_enfant_2018.pdf
Germany/Austria	German ENT society	ci-weissbuch-20-inkl-anlagen-datenblocke-und-zeitpunkte-datenerhebung-mit-logo-05-05-21.pdf
		017-071l_S2k_Cochlea-Implantat-Versorgung-zentral-auditorische-Implantate_2020-12.pdf
Italy	Italian Society of Otorhinolaryngology	Cochlear implant procedure. Italian Clinical Practice Guidelines of the Italian Society of Otorhinolaryngology (SIOeChCF) and Italian Society of Audiology and Phoniatrics (SIAF). Part 1: cochlear implants in adults—PMC
Spain	Acta Otorrinolaringológica Española	Guideline on Cochlear Implants | Acta Otorrinolaringológica Española
Nordic countries	Community of CI client associations from the five Nordic countries, including representation from EURO-CIU and CIICA	NORDIC-DECLARATION-FInal.pdf
Poland	Polish ORL societies	Nowa Audiofonologia—Numer 1/2018 vol. 7
South Africa	South African Cochlear Implant Group (SACIG)	https://www.sacig.org.za/
United Kingdom	British Cochlear Implant Group	Overview | Cochlear implants for children and adults with severe to profound deafness | Guidance | NICE
United States	American Academy of Audiology	Clinical Practice Guideline: Cochlear Implants—American Academy of Audiology
Netherlands	Veldnorm Cochleaire Implantatie	Benelux Resources | Adult Hearing
Australia/New Zealand	Government of Western Australia Department of Health	Australian Resources | Adult Hearing

#### 4.6.2 Clinical Effectiveness of Cochlear Implants

CI recipients demonstrate significant improvements in speech reception in both quiet and moderate noise environments. This benefit has been observed in adults experiencing severe, profound, or moderate to profound bilateral sensorineural hearing loss (SNHL). CIs are beneficial, regardless of the cause or age of hearing loss. They can be an effective treatment for hearing loss in adults of all ages, with limited speech perception. In terms of efficacy in speech perception, a review by Boisvert (2020) states that, on a group level, word perception scores with conventional hearing aids improved from 8.2 to 53.9% postimplant for the implanted side. A significant majority of adults (82%) with postlingual hearing loss experienced an individual speech perception improvement of 15 percentage points or more in the implanted ear. This proportion was smaller for adults with prelingual hearing loss (53.4%). It is reasonable to assume that these gains in speech recognition will remain stable over time. Self-reported functional benefits have been well demonstrated across the adult CI population. For example, a modest 12 percentage point improvement has been shown with the quality-of-life measure HUI3.

Notwithstanding the favorable impact, a multitude of factors exert an influence on outcomes following cochlear implantation. Such factors may include etiology, duration of hearing loss, and age at implantation.

Engagement in postoperative rehabilitation is imperative for achieving optimal hearing outcomes with a cochlear implant. As stated in the consensus statement following cochlear implant activation after surgery, the recipient should receive implant programming and rehabilitation sessions to optimize performance.

Although CIs restore sound access, they do not guarantee real-world speech communication abilities. To optimize communication outcomes, clients have access to numerous auditory training (AT) approaches, including client-directed and HCP-guided interventions. Evidence suggests that auditory training (AT) can improve auditory outcomes for postlingually deafened adults who use CIs, when compared to those who do not receive any training. The primary objective of auditory training is to enhance auditory abilities. However, recent findings indicate the potential for cognitive improvements as well. Mosnier et al (2018) were the first to conduct research on CIs in older adults, demonstrating a substantial enhancement in clients' global cognition, episodic memory, processing speed, and executive function 12 months following implantation, particularly in those with preoperative cognitive impairment. The contemporary conception of CI provision indicates that auditory rehabilitation through CIs has an association with cognitive abilities.

#### 4.6.3 Cost-Benefit of Cochlear Implants

Unilateral and sequential bilateral cochlear implantation appear to be cost-effective when compared with bilateral hearing aids or nontechnological support. When indicated appropriately, simultaneous bilateral cochlear implants are cost-effective when compared with no interventions, as well as with unilateral cochlear implantation, through the use of differential discounting or variations from the base cases. This cost-effectiveness is especially evident in cases where life expectancy is 5 to 10 years or more. According to the 2021 World Health Organization (WHO) report, the implementation of cochlear implants results in significant cost savings within health and social care systems. These savings are attributed to the prevention of disability and the subsequent reduction in societal costs associated with disability management.

The Global Burden of Disease (GBD) study on hearing loss, which informed the WHO report, also advanced the following argument: It is evident that early rehabilitation, in conjunction with the utilization of hearing devices such as cochlear implants, is a cost-effective approach. This assertion is made despite the substantial costs associated with the initial technology investments and the potential risks of infectious complications that may arise following implantation.

Recommendations (see evidence Appendix 1: Technology, 4; Sections 4.6: Cochlear Implants and 4.6.3: Cost Benefit of CI)

1. Clients' hearing aid fitting must be optimal. HCPs should ensure additional technologies such as remote microphones and other assistive listening devices are prescribed where appropriate.
2. It is recommended that consideration be given to referral for a cochlear implant at a stage long before the point of failure with hearing aids. Hearing aids should not be considered as the final stage in the process of managing one's hearing loss.
3. A referral by an HCP is essentially a recommendation that their client should seek further information about cochlear implants. The decision regarding candidacy will be made by a multidisciplinary team.
4. It is imperative to ensure that the referral pathway remains uncomplicated and readily comprehensible. It is incumbent upon HCPs to establish connections with local cochlear implant centers, with a view to encouraging queries and acquiring an understanding of the local pathway. See Table 2 .
5. It is important for the HCP to feel confident about returning to this conversation at regular points in the client pathway. This is because it is often a process rather than a one-off occurrence.

(See Appendix 3: Toolkit; Table 17 .)

#### 4.6.4 Understand the Benefits of Bimodal Fittings

The continuum of care, which begins with the use of hearing aids and ends with the fitting of cochlear implants, achieves the best possible hearing by using both ears. A bimodal fitting is defined as a hearing aid that is fitted to one ear and a cochlear implant to the other. In isolation, aided acoustic hearing does not necessarily guarantee a high level of speech understanding. However, research has demonstrated that bimodal listeners exhibit a considerably higher level of speech comprehension and auditory acuity when utilizing a cochlear implant together with a hearing aid as opposed to relying solely on a cochlear implant or a hearing aid alone (Firszt et al, 2023; Mertens et al, 2024; Casarella et al, 2024).

Recommendations (see evidence Appendix 1: Technology, 4; Sections 4.6: Cochlear Implants and 4.6.4: Understand the Benefits of Bimodal Fittings)

1. HCPs should keep in mind that cochlear implant candidates will continue to use and require regular care for their hearing aids after implantation. Candidates should therefore be motivated to continue to use their hearing aids.
2. HCPs are advised to emphasize the need for fine-tuning the hearing aids, to monitor them, and to adapt them according to the client's hearing with CI.

(See Appendix 3: Toolkit; Table 18 .)

#### 4.6.5 Understand the Limitations of Other Implantable Devices for this Population

In recent years, there has been a notable increase in the availability of auditory implants, thus providing a wider range of treatment options. This has engendered a degree of uncertainty among HCPs who do not specialize in auditory implants when determining the optimal choices for their clients.

Middle ear implants function by utilizing mechanical energy to stimulate the inner ear. This objective is realized through the implementation of an implanted vibrational transducer, which is connected to the ossicles, the oval window, or the round window membrane. Bone-conduction hearing implants, also referred to as bone-anchored implants, employ bone conduction from an external sound processor to stimulate the auditory system. This is achieved through percutaneous coupling with an osseointegrated titanium implant, transcutaneous magnetic coupling with a titanium implant, or transcutaneous delivery through traditional oscillatory bone conduction with the sound processor on a soft or hard band. This procedure bears a resemblance to the technique of bone conduction audiometry.

Middle ear implants require a fully functional middle ear system, and both middle ear and bone-conducting implants rely on adequately functioning inner hair cells for cochlear stimulation, as 95% of afferent auditory nerve fibers are connected to inner hair cells.

The purpose of auditory brainstem implants is to address total hearing loss in both ears, which can result from damage to the eighth nerve caused by tumors or surgical procedures. These devices are intended for clients whose hearing cannot be adequately addressed using hearing aids or cochlear implants. The procedure is indicated for a select group of clients with complete hearing loss for whom no effective alternative treatment exists. The number of these procedures remains low and is performed in a limited number of hospitals.

Recommendations (see evidence Appendix 1: Technology, 4; Sections 4.6: Cochlear Implants and 4.6.5: Understand the Limitations of Other Implantable Devices for this Population)

1. For individuals with bilateral severe and profound sensorineural hearing loss, typically neither middle ear implants nor bone-anchored implants are viable treatment options.
2. HCPs should seek information and support from relevant medical professionals about Auditory Brainstem Implants for specific clients.

(See Appendix 3: Toolkit; Table 19 .)

(See Appendix 4: Clinical Checklist 6.)

### 4.7 Remote Fittings

**Table:** 

Remote fitting encompasses two distinct processes: initial fittings and fine-tuning/follow-up. Initial fittings refer to the first programming and verification of hearing aids. For adults with S/PHL, these are best conducted in person using probe microphone measures, due to the need for precision, safety, and assessment of CI candidacy. Fine-tuning and follow-up involve subsequent adjustments, verification, and counseling, and these have stronger evidence for remote delivery. Both approaches increasingly use tele-audiology platforms, in situ audiometry, and community-facilitated models to expand access to care (Dillard et al, 2024; Frisby et al, 2022). Looking ahead, remote fittings represent a significant opportunity for the cochlear implant industry. While remote programming solutions are already technologically feasible and available, adoption has progressed more slowly than in the hearing aid market. As reimbursement policies continue to evolve, remote services have the potential to enhance accessibility, convenience, and patient care, further advancing the delivery of cochlear implant services worldwide

#### 4.7.1 Remote Initial Fittings

Current evidence for Severe and Profound Hearing Loss (S/PHL) is limited. While strong correlations have been shown between remote and in-person measures of real-ear insertion gain and audiograms (Venail et al, 2019), individuals with S/PHL face challenges including narrow dynamic ranges, earmold fit, and safety concerns in setting MPOs (Benson et al, 2018). Remote initial fittings may yield poorer outcomes for speech-in-noise compared to in-person fittings, so that the best practice emerging from systematic reviews and international guidance (ASHA, 2021; Audiology Australia, 2022; BAA/BSA, 2021; Swanepoel, in press) emphasizes blended care: an in-person initial fitting with REM, followed by remote adjustments.

Recommendations (see evidence Appendix 1: Technology, 4; Sections 4.7: Remote Fittings and 4.7.1: Remote Initial Fittings)

1. An in-person initial fitting with probe microphone verification remains the gold standard for adults with severe-to-profound hearing loss. Remote initial fittings should only be undertaken when in-person access is not possible and with robust safeguards in place (e.g., recent audiogram, measured RECD, technical support).
2. When remote initial fittings are conducted, conservative MPO settings should be applied with formal loudness comfort assessments, and candidacy screening for cochlear implantation must be included.
3. Coupler-based verification with measured RECD may be used as a conditional alternative when probe microphone measures are unavailable. Use of average RECD values alone is not recommended due to the high risk of under- or over-amplification.

#### 4.7.2 Remote Fine-Tuning and Follow-Up

By contrast, evidence for remote fine-tuning and follow-up is considerably stronger. Systematic reviews (Tao et al, 2018) and randomized controlled trials (Tao et al, 2021; Venail et al, 2019; Malmberg & Hagberg, 2024, 2025) demonstrate that remote follow-up provides outcomes comparable to in-person care across domains such as communication benefit, client satisfaction, and hearing-specific quality of life. Validated client-reported outcome measures (PROMs) such as the IOI-HA (Cox & Alexander, 2002), APHAB (Cox & Alexander, 1995), SSQ-12 (Noble et al, 2012), SADL (Cox ¬Alexander, 1999), and COSI (Dillon et al, 1997) have been successfully used in remote contexts (Kokkonen et al, 2023), providing reliable means of outcome monitoring. Remote hearing aid replacement pathways where new hearing aids were programmed according to the audiometric data on file are feasible, with minimal differences in outcomes compared to conventional refitting (Kokkonen et al, 2023).

Recommendations (see evidence Appendix 1: Technology, 4; Sections 4.7: Remote fittings and 4.7.2: Remote Fine-Tuning and Follow-Up)

1. Remote fine-tuning and follow-up after an in-person initial fitting can be routinely offered synchronously or asynchronously. Randomized controlled trials and agreement studies demonstrate comparable outcomes to in-clinic visits.
2. Remote follow-up should include verification using in situ speech mapping where available, supplemented by validated client-reported outcome measures (PROMs) such as the Client-Oriented Scale of Improvement (COSI; Dillon, James & Ginis, 1999), Speech, Spatial and Qualities of Hearing Scale (SSQ-12; Noble et al, 2013), Satisfaction with Amplification in Daily Life (SADL; Cox et al, 1999), International Outcome Inventory for Hearing Aids (IOI-HA; Cox et al, 2002), Hearing Aid User's Questionnaire (HAUQ; Dillon, Birtles & Lovegrove, 1999), and Abbreviated Profile of Hearing Aid Benefit.
3. Remote microphones and wireless accessories should be optimized during follow-up sessions, as they are essential for speech understanding in noise.
4. Structured follow-up intervals (e.g., 2 weeks, 4–6 weeks, and quarterly in the first year) should be maintained, whether in-person or remote, to support acclimatization and identify CI candidacy where appropriate. RCTs confirm that both short- and long-term outcomes are comparable when remote fine-tuning is used.
5. Community-based facilitators, including trained health workers or supervised assistants, may support remote follow-up in underserved contexts, provided that HCPs remain responsible for programming and verification. Evidence highlights the essential role of trained facilitators in enabling safe and effective consultations.
6. HCPs and clients should receive appropriate training and educational resources to ensure effective participation in remote fine-tuning and troubleshooting. Professional guidelines emphasize the importance of preparation and training for safe and effective remote care. Client and clinician perspectives also highlight the value of shared decision-making.
7. Remote renewal of hearing aids using preexisting audiograms may be considered for stable, experienced users, with in-person follow-up reserved for those presenting new symptoms or insufficient benefit.

#### 4.7.3 Client and HCP Perspectives

Research on perspectives indicates that clients value the convenience of remote services but often prefer in-person fittings for initial appointments, while HCPs highlight challenges with rapport, verification, and troubleshooting but recognize that remote models improve access and efficiency (Blyth & Saunders, 2024).

#### 4.7.4 Infrastructure and Future Directions

Infrastructure and training requirements remain central to successful delivery, including stable connectivity, client digital literacy, and facilitator support (DiFabio et al, 2022). Looking ahead, remote fitting is increasingly positioned as part of connected hearing healthcare, with AI-driven personalization and integrated digital ecosystems expected to further enhance outcomes (Ferguson, 2023).

#### 4.7.5 Safety and Risk Management

Remote initial fittings carry higher risks and require safeguards: conservative MPO limits, CI candidacy screening, identity verification, informed consent, secure platforms, and privacy protection. International guidelines emphasize maintaining equivalent safety standards across remote and in-person care (BAA/BSA, 2021; Audiology Australia, 2022). Technical reliability and facilitator training are critical to safety and satisfaction (DiFabio et al, 2022).

#### 4.7.6 Special Considerations for Severe and Profound Hearing Loss

For adults with S/PHL, precision and individualized management are essential. Frequency-lowering should be considered case by case, with parameter optimization and verification of benefit (speech cues, sound quality); discontinue if detrimental (Glista et al, 2009; Alexander, 2013). Remote microphones and wireless accessories are standard adjuncts. Client selection is key: experienced users with recent RECD and digital literacy are well suited to remote models, while new users or those with complex earmold needs are best managed in person.

(See Appendix 3: Toolkit; Table 20 .)

(See Appendix 4: Clinical Checklist 8.)

## 5. Rehabilitation and Other Treatment Considerations

### 5.1 Rehabilitation: Communication and Psychosocial Concerns

**Table:** 

Rehabilitation for clients with severe and profound hearing loss extends beyond technology. It involves enabling clients to make optimal use of auditory devices while supporting their psychosocial adjustment, communication effectiveness, and social participation. Effective rehabilitation should therefore address: • How an individual adjusts to life with severe and profound hearing loss, supported collaboratively by an HCP. • Psychosocial support for families and caregivers to strengthen resilience, communication, and shared well-being. • Training to develop adaptive and effective communication strategies, including guidance to understand how they can modify the communication behavior of communication partners in a supportive way. • Building psychosocial support networks within the wider society, through peer connection, social coaching, and community participation to enhance inclusion and reduce isolation

#### 5.1.1 Addressing the Individual Psychosocial Impact of Severe and Profound Hearing Loss

Many of the challenges that people with severe and profound hearing loss face in their everyday lives are caused by psychosocial factors, that is, emotional, psychological, and environmental factors that influence a person's physical, mental, and functional wellness. Psychosocial impacts of hearing loss include stigma, emotional distress (including depression and anxiety), social isolation, and loneliness (Ekberg & Hickson, 2025; Bigelow et al, 2020; Shukla et al, 2020). Technology plays an important role in treatment options for severe and profound hearing loss, but addressing the psychosocial impact of hearing loss through a holistic approach to rehabilitation is equally important. The HCP must therefore take steps to offer psychosocial support where needed to help clients manage their hearing in everyday life, beyond providing hearing technologies.

Recommendations (see evidence Appendix 1: Rehabilitation and Other Treatment Considerations, 5; Sections 5.1: Rehabilitation: Communication and Psychosocial Concerns and 5.1.1: Addressing the Individual Psychosocial Impact of Severe and Profound Hearing Loss)

The HCP should:

1. Ensure accessible and inclusive communication in the clinic—Confirm that clients can follow discussions using their preferred communication mode, spoken, signed, written, or captioned. The HCP should be familiar with the local communication support options or have other strategies for communication, such as typed notes, interpreters, or communication apps.
2. Use clear, individualized collaborative communication—use appropriate language and terminology individualized to the client in communicating any information and advice. Inadequate verification of the client's understanding is the single most common reason for limited compliance with recommendations and for HCPs not providing appropriate interventions.
3. Explore each individual's adjustment and strengths—discuss severe communication challenges they face. These factors vary with personality, the impact of hearing loss stigma, family and other circumstances, changes in identity due to hearing loss, sources of support, additional health issues, and a person's hearing history. This information should inform individualized person-centered counseling to support personal adjustment.
4. Assess psychosocial impact of the hearing loss. A range of approaches can be used including direct questioning, probing, and self-report screening surveys such as The Patient Health Questionniare-4 (PHQ4; Kroenke et al, 2009), Action Questionnaire—Adult Hearing Loss (AAQ-AHL; San Miguel et al, 2022), Social Isolation Measure (SIM; Heffernan et al, 2019), Hearing Handicap Inventory for the Elderly (HHIE-S; Ventry & Weinstein, 1983), Revised Hearing Handicap Inventory (RHHI; Casserly et al, 2020) and My Hearing PREM-16, and My Hearing PREM-9; Smith et al, 2025). See Appendix 3; Table 21 . One question that HCPs can ask clients to reveal whether they experience stigma related to their hearing loss is: “Who do you tell about your hearing loss?” (Ekberg et al, 2025).
5. Incorporate socio-emotional needs and goals in an individualized management plan. This plan can be an interactive document and include: (1) agreed needs; (2) planned actions; (3) completed actions; and (4) outcomes. Tools such as the Client Oriented Scale of Improvement (COSI; Dillon et al, 1997) can help to develop and structure these individualized plans.
6. Integrate psychosocial and hearing goals—relate the identified hearing and socio-emotional needs and goals to recommendations, including hearing devices, and auditory and communication training. It is important for the HCP to offer options to clients. Options should be delivered in a holistic, person-centered approach with the HCP partnering with the client, empowering them, promoting client responsibility, providing emotional support, and supporting them to adhere to the treatment interventions they have considered. See Section 1.4.
7. Promote Motivation and self-management skills—this should go far beyond giving instruction/information to bring about the behavior change necessary for clients with severe and profound hearing loss to achieve maximum amplification satisfaction and outcomes. Motivational engagement (strategies to support clients to improve their readiness and self-efficacy for hearing rehabilitation) should be used, and the client should be offered the opportunity to develop effective self-management techniques.
8. Collaboratively set and review realistic goals—explore the client's beliefs about their outcomes with all the chosen interventions outlined in their individual management plan and help manage expectations at regular parts of their pathway. See Sections 1.3 and 1.4.
9. Identify mental health needs and support well-being—early consideration and onward referral where appropriate are essential to ensure the client can derive maximum benefit from hearing devices and rehabilitation. The incidence of clinical depression and anxiety in clients with severe and profound hearing loss is high. Programs such as the Ask, Inform, Manage, Encourage, Refer (AIMER) can be used to help (Bennett et al, 2024) HCPs provide mental wellbeing support to clients and know when and how to refer to other mental health specialists. See Section 1.2.
10. Encourage ongoing growth and connection—help educate the client with self-management strategies, where appropriate, such as conversation repair strategies, lip reading, and adapting their environment.

(See Appendix 3: Toolkit; Table 21 .)

#### 5.1.2 Incorporating Family in Rehabilitation for Shared Psychosocial Well-Being

A severe to profound degree of hearing loss impacts not only the individual but can have a significant impact on their communication partners, such as family, friends, and caregivers. Sometimes termed “third-party disability,” hearing loss may result in activity limitations, participation restrictions, and psychosocial impacts experienced by the adult's communication partners. To maximize hearing loss management outcomes, HCPs should adopt a family-centered approach. Family, as used here, is understood in its broadest sense, encompassing not only partners and relatives but also anyone who holds an important place in the individual's life.

A family-centered approach to care seeks to integrate family members throughout every stage of hearing care, beginning with appointment booking, continuing through assessment, rehabilitation, and extending to follow-up services. When recommending management options, HCPs should assess and consider the requirements of the individual's family members as well. Including family in goal setting and decision making and providing them with education and support can result in improved outcomes and shared psychosocial well-being.

HCPs also should address the role of stigma and whether family members may be experiencing affiliate stigma. Although family members of adults with hearing loss may report minimal affiliate stigma, they do experience emotions of sadness and powerlessness, and connect hearing loss and hearing devices with unfavorable stereotypes.

Recommendations (see evidence Appendix 1: Rehabilitation and Other Treatment Considerations, 5; Sections 5.1: Rehabilitation: Communication and Psychosocial Concerns and 5.1.2: Incorporating Family in Rehabilitation for Shared Psychosocial Well-Being)

The following issues are important to consider.

1. The impact of the client's hearing loss on their close friends and family (third-party disability) should be considered as part of the needs assessment to develop effective intervention strategies.
2. Invite family members to attend and be active participants during audiology appointments.
3. Assess if family members are experiencing third-party disability. This can be done through direct questioning or the use of self-report questionnaires such as the Hearing Impairment Impact—Significant Other Profile (HII-SOP; Preminger & Meeks, 2012) or the Significant Other Scale for Hearing Disability (SOS-HEAR; Scarinci et al, 2009; see Appendix 3; Table 14 ). As family members may experience affiliate stigma, the use of a questionnaire such as the Affiliate Stigma Scale (Mak & Cheung, 2008; Chang et al, 2015), adopted for family members of people with hearing loss, is also recommended.
4. Incorporate information and support for the client's family in the hearing loss management plans. This should include information on how family members may support the use and management of hearing devices such as hearing aids, cochlear implants and other hearing technologies.
5. Engage family members in shared goal setting and decision-making to ensure hearing management outcomes are optimized for the adult with hearing loss and their significant communication partners. Support for family members can also be through involvement in auditory, communication, and social support programs.
6. Measure rehabilitation outcomes not only for the individual with severe and profound hearing loss but also for their family members. Useful self-report questionnaires to help with this include the Significant Other Assessment of Communication (SOAC; Schow & Nerbonne, 1982) and the International Outcome Inventory for Hearing Aids for the Significant Other (SO; IOI-HA-SO; Noble, 2002).

(See Appendix 3: Toolkit; Table 22 .)

#### 5.1.3 Building Psychosocial Support in the Community and Workplace

Severe and profound degrees of hearing loss can reshape how individuals connect with the world around them. Communication barriers often arise from environmental and social inaccessibility rather than individual deficits. When unaddressed, these psychological consequences may lead to withdrawal from social contact, adverse mental health outcomes, accelerated cognitive decline, and lower self-esteem.

To promote social connection and wellbeing, HCPs should integrate social and emotional rehabilitation. This involves assessing the client's social and emotional needs, collaboratively setting meaningful goals, engaging family and communication partners to support progress, implementing tailored interventions such as social coaching, activity scheduling, and peer support, and regularly monitoring and reflecting on outcomes to adapt strategies and enhance social participation, coping, and overall psychosocial well-being.

Recommendations (see evidence Appendix 1: Rehabilitation and Other Treatment Considerations, 5; Sections 5.1: Rehabilitation: Communication and Psychosocial concerns and 5.1.3: Building Psychosocial Support in the Community and Workplace)

The hearing healthcare provider should provide support for communication and social skill engagements, workplace and community barriers, peer support, and social participation. The following tools and approaches can be used.

1. Use structured tools such as the Communication Rings, Social Participation Restrictions Questionnaire (SPaRQ; Montano & Makadma, 2012; Heffernan et al, 2019) to assess social support needs.
2. Offer communication and social skills training, including lipreading, auditory training, advocacy, assertiveness, and conflict resolution.
3. Integrate social coaching into rehabilitation—structured guidance aimed at building social confidence, developing adaptive interaction strategies, and re-engaging in social settings. Evidence-based group programs such as the Active Communication Education (ACE; Hickson et al, 2007) can support this.
4. Monitor for psychological distress, withdrawal, or loneliness, and provide referrals to mental health professionals where appropriate.
5. Discuss workplace accommodations and communication strategies. Recommend the implementation of buddy systems or workplace communication champions to provide immediate communication support and advocacy.
6. Offer advocacy coaching to help clients confidently request reasonable accommodations and educate employers or colleagues about their hearing needs.
7. Encourage clients to meet others who share a similar hearing history and degree of hearing loss, but most importantly, share an understanding of the problems they are facing. Peer support reduces stigma, fosters acceptance, and offers insights that extend beyond professional perspectives.
8. Develop and maintain a network of peer mentors, including adults with severe and profound hearing loss who are well-adjusted and trained to provide support. Utilizing this network offers small-group rehabilitation programs (structured or self-directed) where clients can share strategies, challenges, and coping mechanisms.
9. Utilize the Expanded Chronic Care Patient-Professional Partnership Model (E2C3PM) of co-production (Pomey et al, 2024), where clients, families, HCPs, and communities collaborate as equal partners to shape interventions and support systems.
10. Support clients in developing new social roles and a renewed sense of identity through meaningful engagement. This could include linking clients with community-based activities (e.g., exercise groups, art classes, hobby clubs) that foster social connection and well-being.

#### 5.1.4 Addressing Psychosocial Impact for Deaf Sign Language Users

For adults who are Deaf and use sign language, psychosocial well-being depends on communication access, identity, and inclusion within Deaf and hearing communities. The impact stems from social and environmental barriers rather than hearing loss. Many identify culturally as Deaf rather than someone with a hearing loss, and interventions should focus on:

- Communication and access.
- Cultural respect.
- Culturally appropriate psychosocial assessments.
- Collaborative goal setting.
- Peer and community connection.

Clinical implication: There are few psychosocial tools for Deaf sign language users. Professionals should think creatively and develop cultural competency toolkits.

(See Appendix 4: Clinical Checklist 9.)

### 5.2 Tinnitus

**Table:** 

Priority should be management of any distress associated with the tinnitus and best practice management of the severe and profound hearing loss. A multidisciplinary approach that includes medical, psychological, and audiological disciplines is strongly recommended. The resulting best practice recommendations are based on established tinnitus guidelines (Lewis et al, 2020; Sherlock et al, 2025), when they are generalized to severe and profound hearing loss, and a state-of-the-art review (2019–2025) of tinnitus accompanying severe and profound hearing loss

#### 5.2.1 Definitions, Prevalence, and Generalization of Guidelines to this Population

According to De Ridder et al (2021), tinnitus can be defined as: “the conscious awareness of a tonal or composite noise for which there is no identifiable corresponding external acoustic source.” Tinnitus is highly heterogeneous, and the degree of distress it causes varies widely between people. Problem tinnitus has been defined as Tinnitus Disorder: “tinnitus is associated with emotional distress, cognitive dysfunction, and/or autonomic arousal, leading to behavioral changes and functional disability” (De Ridder et al, 2021). Tinnitus can be either objective or subjective. Objective tinnitus (also called somatosound) refers to the perception of sound that also can be heard by the examiner and is usually due to blood flow or muscle movement. Most commonly, tinnitus is subjective (true tinnitus); the sound is heard only by the person experiencing it, and no source of the sound is identified. Acute tinnitus may spontaneously recover within minutes to weeks. Chronic tinnitus is unlikely to resolve spontaneously when experienced for 3 months or more.

#### 5.2.2 Tinnitus Management

Tinnitus management practices recommended in the literature are largely independent of the degree of hearing loss, and participants in most studies have normal to moderate hearing loss. Tinnitus in the presence of severe and profound hearing loss is seldom considered in existing tinnitus research and guidelines. However, most tinnitus therapy guidelines will be broadly applicable to individuals with severe and profound hearing loss if the hearing loss is accounted for. Adaptations may be necessary when hearing loss significantly limits auditory input (e.g., sound-based therapies) or makes communication difficult (e.g., talk therapies). Our recommendations assume, in the absence of evidence to the contrary, that general guidelines are appropriate once the challenges posed by severe and profound hearing loss are addressed.

##### 5.2.2.1 Assessment and Referrals

Recommendations (see evidence Appendix 1: Rehabilitation and Other Treatment Considerations, 5; Sections 5.2: Audiological Management of Tinnitus in Severe and Profound Hearing Loss, 5.2.2: Tinnitus Management, and 5.2.2.1: Assessment and Referrals)

1. The HCP should conduct a careful and thorough tinnitus history as part of the diagnostic assessment. An assessment of tinnitus impact on the person using an instrument such as The Tinnitus Functional Index (TFI; Meikle et al, 2012) also should be undertaken.
2. The HCP should make an immediate referral to a crisis mental health management team for people with distressing tinnitus associated with the risk of suicide.
3. The HCP should undertake a thorough audiological assessment to identify the degree of hearing loss and any ear pathology.
4. Psychoacoustic measures (e.g., minimum masking level, loudness matching) are not recommended for tinnitus assessment in this population.
5. The HCP should make an immediate medical referral for sudden hearing loss or neurological symptoms (e.g., facial weakness).
6. The HCP should make a referral to Otology to exclude underlying medical abnormalities and obtain an opinion as to medical treatment to relieve the tinnitus.
7. Discussion of the reasons for assessment, referrals, and management needs to be clear and cognizant of any communication difficulties.
8. The HCP should undertake a needs assessment with the client that considers their preferences for therapy.

##### 5.2.2.2 Medical Management

Recommendations (see evidence Appendix 1: Rehabilitation and Other Treatment Considerations, 5; Sections 5.2: Audiological Management of Tinnitus in Severe and Profound Hearing Loss, 5.2.2: Tinnitus Management, and 5.2.2.1: Assessment and Referrals)

1. There is insufficient evidence to support pharmacotherapy for tinnitus management in this population.

##### 5.2.2.3 Psychological Therapies

Recommendations (see evidence Appendix 1: Rehabilitation and Other Treatment Considerations, 5; Sections 5.2: Audiological Management of Tinnitus in Severe and Profound Hearing Loss, 5.2.2: Tinnitus Management, and 5.2.2.2: Medical Management)

1. Talk-based therapies need to accommodate the hearing abilities of the client.
2. Written material needs to accommodate the literacy of the client.
3. Educational counseling should be provided to reduce the functional impact of tinnitus.
4. The content should be tailored to the individual needs of the person and significant others.
5. For disturbing tinnitus: one-to-one, group, or digital tinnitus-related cognitive behavioral therapy (CBT) provided by a psychologist should be offered. The intention of CBT is to modify dysfunctional behaviors and beliefs of the clients to reduce the tinnitus distress and to increase the quality of functioning in daily life (e.g., improved sleep).

##### 5.2.2.4 Hearing Assistance

Recommendations (see evidence Appendix 1: Rehabilitation and Other Treatment Considerations, 5; Sections 5.2: Audiological Management of Tinnitus in Severe and Profound Hearing Loss, 5.2.2: Tinnitus Management, and 5.2.2.4: Hearing Assistance)

1. Counsel the client that treating the hearing loss is likely to bring some relief from tinnitus.
2. Addressing the hearing loss is a priority. If the hearing loss is aidable, the hearing aid fitting should ensure the maximum audibility possible for environmental sounds and speech.
3. If the hearing loss is unaidable, consider referral for cochlear implant assessment to address the hearing loss.

##### 5.2.2.5 Sound-Based Therapies

Recommendations (see evidence Appendix 1: Rehabilitation and Other Treatment Considerations, 5; Sections 5.2: Audiological Management of Tinnitus in Severe and Profound Hearing Loss, 5.2.2: Tinnitus Management, and 5.2.2.5: Sound-Based Therapies)

1. Sound enrichment through amplified, streamed, or hearing aid-programmed sound may be used periodically to provide relief when accompanied by psychoeducation or CBT.
2. Treatment using tinnitus noise generators in hearing aids should be used with extreme care when severe and profound hearing loss is present to avoid sound-induced hearing loss.
3. The user should be informed that the added sound may interfere with hearing speech or warning sounds in their environment.

##### 5.2.2.6 Other Therapies

Recommendations (see evidence Appendix 1: Rehabilitation and Other Treatment Considerations, 5; Sections 5.2: Audiological Management of Tinnitus in Severe and Profound Hearing Loss, 5.2.2: Tinnitus Management, and 5.2.2.6: Other Therapies)

1. There is insufficient evidence to recommend for or against the use of web- or app-based self-management. Sound-based methods may not be audible in this population.
2. There is insufficient evidence to recommend for or against the use of perceptual or attention training programs. Auditory training sounds may not be audible in this population.
3. There is insufficient evidence to recommend for or against neurostimulation/neuromodulation (e.g., Transcranial Direct Current Stimulation (TDCS), Transcranial Magnetic Stimulation (TMS)) in this population.
4. There is insufficient evidence to recommend for or against acupuncture.
5. The HCP should not recommend the use of dietary supplements or herbs for tinnitus management.

(See Appendix 3: Toolkit; Table 23 .)

(See Appendix 4: Clinical Checklist 10.)

### 5.3 Genetic Hearing Loss and Gene Therapy for Hearing Loss

This section of the Guidelines focuses on hereditary factors associated with severe and profound hearing loss and potential options including gene therapy. While this professional area is out of scope for most HCPs in clinical practice, the guidance in this document is aimed at multidisciplinary teams that include specialists in genetic counseling and otology. It is beyond the scope of this document to provide a tutorial in genetics. Terminology used within this section is very specialized, and the reader is advised to consult with colleagues who are professionally involved with this subject area. This section could be a useful resource as an overview if information is requested by the individual with severe and profound hearing loss (or family members) during a clinical consultation with an HCP.

**Table:** 

Genetic factors account for approximately 50–70% of congenital and early-onset hearing loss, with more than 150 hearing loss genes implicated. • Approximately 70% of genetic (hereditary) hearing loss is nonsyndromic (hearing loss that occurs in isolation, without other medical conditions or physical features). Within nonsyndromic hearing loss, 75–80% is inherited in an autosomal recessive pattern (a child must inherit a nonworking copy of the gene from both parents to develop the condition; parents who each carry one nonworking copy are typically unaffected). Figures vary by cohort and ancestry. • Most genetic hearing loss is monogenic (caused by a change in a single gene). • GJB2 is the single most common cause of autosomal recessive nonsyndromic hearing loss, accounting for up to approximately 50% of autosomal recessive nonsyndromic hearing loss in some populations; the proportion varies substantially by ancestry and cohort. • Autosomal recessive loss-of-function (conditions where both copies of a gene are nonfunctional, meaning the body cannot produce a working version of the associated protein) conditions are the most tractable early targets for gene supplementation (a therapeutic approach that delivers a working copy of the faulty gene into cells). • Genomic testing is increasingly available for diagnosing genetic variants as a cause of hearing loss. • Traditional interventions—hearing aids and cochlear implants provide functional compensation by amplifying sound or electrically stimulating the auditory nerve directly, yet they fail to address the underlying molecular pathology or repair hearing mechanisms. • AAV vectors (Adeno-Associated Viruses—generally harmless, modified viruses used as delivery vehicles to carry therapeutic genes into cells) are the leading platform for cochlear delivery; payload limits (the maximum amount of genetic material an AAV can carry) drive dual-AAV strategies (using two AAV vectors together to deliver a gene too large to fit in one) for large genes (e.g., otoferlin (OTOF)). • First-in-human OTOF/DFNB9 trials show rapid, clinically meaningful hearing gains with mostly mild-to-moderate adverse events. • Trials for replacement of other genes are likely to arrive in the near future. • Therapeutic window matters: cochlear hair cells do not regenerate, so earlier intervention is generally more effective. • Long-term safety and durability monitoring are essential (auditory, vestibular, immunologic, and systemic surveillance)

#### 5.3.1 Candidate Identification and Diagnostic Workup

To define eligibility criteria for inner-ear gene therapy trials, specify the type of genetic testing required, outline limitations, and describe the minimum diagnostic workup needed to match genotype to hearing loss or cochlear phenotype.

Recommendations (see evidence Appendix 1: Rehabilitation and Other Treatment Considerations, 5: Sections 5.3: Genetic Hearing Loss and Gene Therapy for Genetic Hearing Loss and 5.3.1: Candidate Identification and Diagnostic Workup)

Consider offering genetic testing (with genetics/audiovestibular counseling) to:
  - All infants/children with confirmed permanent bilateral SNHL or ANSD (including those detected via newborn screening) with no other explanatory cause.
  - Adults with early-onset hearing loss, progressive loss, a positive family history, consanguinity, or features suggesting syndromic disease.
  - Unilateral hearing loss if there are syndromic features or a family history consistent with a monogenic cause.
Obtain appropriate consent following established guidelines, covering:
  - Purpose and scope of testing (targeted just variant gene versus gene panel versus WES/WGS—(whole exome sequencing/whole genome sequencing)).
  - Possible outcomes (pathogenic/likely pathogenic, negative, VUS (variant of unknown significance)) and what each does—and does not—mean.
  - Technical limitations and blind spots (areas not well covered (including the possibility of reanalysis/updated interpretation over time).
  - Policy on secondary/incidental findings (especially for WES/WGS).
  - Implications for relatives (cascade testing, reproductive risk, and how familial information may be handled within confidentiality).
  - DNA/data storage and data sharing for interpretation/quality assurance.
  - How results will be returned (who, when, what follow-up) and the service's approach to recontacting.
  - Special considerations for children (assent, best interests) and adults lacking capacity.
  - Documentation requirements (a contemporaneous record of the discussion/consent per local pathway).
  - UK-specific practicalities such as the Government/ABI Code on genetic testing and insurance.
Ensure access to pre- and posttest genetic counseling. Results often have familial and reproductive implications and can uncover uncertain or unexpected findings that require careful interpretation.
Document the phenotype to interpret the genetics. A clear, structured phenotype is essential to judge whether a reported variant, especially a variant of unknown significance (VUS), fits the expected clinical picture and to identify situations where a “negative” result may reflect an undetected second allele (e.g., CNV, low coverage region) rather than absence of a genetic cause. Careful genotype–phenotype correlation also strengthens variant classification and helps prioritize reanalysis. Phenotyping may include:
  - Pure tone audiogram.
  - Auditory brainstem response (ABR).
  - Otoacoustic emissions.
  - Vestibular testing.
  - Imaging.
  - Whole-body examination of other organs or body parts, or laboratory testing.
Use a comprehensive hearing-loss panel (next-generation sequencing (NGS) with integrated copy number variant (CNV) calling as the first-line genetic test. For clients with suspected hereditary hearing loss, this curated multigene panel approach, covering both common nonsyndromic and syndromic hearing-loss genes alongside deletion/duplication detection, achieves diagnostic yields of approximately 30 to 40% and represents the most efficient balance of clinical utility, cost, and interpretability for most clients. The 2022 ACMG clinical practice resource recommends that, for individuals lacking features of a specific syndrome, comprehensive hearing-loss gene panel testing should be initiated as a tiered diagnostic approach. Laboratories and HCPs should select panels curated using robust gene–disease validity frameworks (such as those applying ClinGen criteria or maintained in resources like Genomics England Panel App) to minimize interpretive noise from genes with weak or insufficiently validated disease associations. As sequencing costs continue to decline and bioinformatic tools improve, whole-genome sequencing (WGS) may become the preferred first-line approach in the future, offering superior detection of noncoding variants, structural variants, and complex rearrangements that current panel-based methods can miss.
  Consider proceeding to exome sequencing (WES) or genome sequencing (WGS; or request “hearing loss WES/large panel” if available) when:
  - Panel testing is negative, and suspicion remains high (early-onset bilateral SNHL/ANSD, multiplex families (where more than one member of family is affected), consanguinity—e.g., first cousin partners).
  - There are additional anomalies/developmental issues suggesting a broader syndromic diagnosis.
  - A structural variant is suspected but not resolved.
  - Re-analysis is indicated (new genes/variant reinterpretation).
  If the phenotype strongly suggests a genetic diagnosis but NGS is negative/uncertain, actively consider test blind spots and order orthogonal methods. NGS may be unreliable/incomplete for:
  - High-homology regions/pseudogene interference (classic examples include the gene STRC; also, other highly repetitive/homologous loci, that is regions where the DNA sequence repeats or closely resembles other parts of the genome, where simple NGS can struggle to show variants).
  - CNVs (single-exon to multi-exon deletions/duplications; gene-conversion events). CNVs are changes in the amount of DNA, where a chunk ranging from a single coding segment (exon) to several exons is either deleted or duplicated. A related problem is gene conversion, where one gene is effectively overwritten by the sequence of a near-identical copy elsewhere in the genome.
  - Poorly captured/GC-rich exons, low coverage regions, or platform-specific artefacts. Some exons are simply harder to read accurately, either because their DNA is Guanine-Cysteine rich (which sequencing handles poorly), because too few sequencing reads land there to give confident results, or because of errors specific to the particular sequencing platform being used.
  - Mitochondrial testing is not covered in some panels. Low-level mitochondrial heteroplasmy, where a variant is present in only a small proportion of mitochondrial copies, may fall below reliable detection thresholds depending on the assay.
  - Deep intronic/splice regulatory variants not covered by panel design. Some disease-causing variants sit deep within introns (the noncoding stretches between a gene's coding segments) or in regions that control how a gene is spliced together. Standard panels usually only read the coding parts of genes, so these variants fall outside what the test looks at and get missed.
  If available, ask the lab to confirm that the gene of interest was well covered. Where a deletion or duplication (CNV) is suspected, especially in STRC, consider a dedicated method such as MLPA, qPCR, or ddPCR, which also helps avoid confusion with near-identical pseudogenes. If suspicion remains high, specialist labs can offer long-read sequencing or other targeted assays. Finally, consider re-testing or re-analyzing the data if the original test predated CNV analysis or used an older panel.
Interpret variants using the standard ACMG/AMP classification framework, applying the hearing-loss–specific rules from the ClinGen Hearing Loss Expert Panel so that gene- and phenotype-relevant evidence is weighed consistently. Record how the patient's genotype fits their clinical picture and use this explicitly during interpretation: it helps judge whether a variant is biologically plausible, refine uncertain classifications (particularly variants of uncertain significance), and recognize when an apparently incomplete result (e.g., only one variant found in a recessive condition) may reflect a technical limitation such as a missed CNV or low coverage rather than a true absence of a second variant. For high-stakes decisions, including eligibility for gene therapy trials, consider confirming clinically significant variants with an independent (orthogonal) method and reviewing them within an experienced clinical genetics service, ensuring that any pathogenic or likely pathogenic calls are consistent with the expected pattern of inheritance.
Confirm segregation and offer cascade testing. For recessive conditions, parental testing may be needed to confirm segregation; that is, that the two variants were inherited separately (one from each parent) rather than both residing on the same chromosome, which would leave the diagnosis incomplete. Once the genetic cause is established, cascade testing (sequential genetic testing of at-risk relatives to identify who else carries the variant) should be offered to family members. Families should also be counselled regarding reproductive options, including prenatal or preimplantation genetic diagnosis where appropriate.

#### 5.3.2 Gene Therapy Guidelines

This section focuses on gene replacement therapies, as dominant gene therapies are not yet clinically available for hearing loss, although the principles are similar. The goal is to establish safety protocols for gene therapy, including accurate diagnosis, pre-, intra-, and postoperative monitoring, and reporting.

Recommendations (see evidence Appendix 1: Rehabilitation and Other Treatment Considerations, 5; Sections 5.3: Genetic Hearing Loss and Gene Therapy for Genetic Hearing Loss and 5.3.2: Gene Therapy Guidelines)

##### 5.3.2.1 Predosing

1. Ensure clients and/or parents fully understand the nature of therapy, limitations, risks, testing schedule, and need for long-term surveillance, and have copies of the protocols and consent forms. Patients and/or parents should be aware of alternative therapies such as cochlear implants or hearing aids, and their relative benefits.
2. Ensure regulatory and ethical compliance. Gene therapy, if part of a trial, should comply with national/international regulatory agencies and laws, undergo rigorous ethical review, and be implemented according to good clinical practices. Manufacturers must ensure Good Manufacturing Practices (GMP) with predefined release specifications (identity, purity, potency, sterility/endotoxin) and stability plans. The administering institution must have processes in place to deal with regulatory requirements around advanced therapies. Consider simultaneous binaural gene therapy only if there is proven safety and efficacy of monaural gene therapy.
3. Maintain up-to-date protocols, a transparent and auditable document management system, and contact with ethics authorities, regulatory bodies, sponsors, and CROs for reporting of any protocol deviations or adverse events. Regular audits are advised. Client confidentiality must be respected.
4. Confirm the molecular diagnosis using comprehensive genetic testing (panel or exome) and classify variants using recognized standards, with consensus on the causative gene variant being replaced in gene therapy. Two independent genetic testing/interpretations are ideally advised.
5. Confirm phenotypic concordance with the genotype (e.g., for OTOF, a DFNB9/ANSD pattern with present OAEs/cochlear microphonic and absent/abnormal ABR).
6. Assess cochlear anatomy and patency (CT/MRI as appropriate) and document baseline auditory and vestibular function before intervention. Severe malformations or other concurrent pathological processes in the ear should be contraindications to therapy.
7. Screen for anti-AAV neutralizing antibodies if required by protocol and document prior AAV exposure where relevant, if AAV is the vector used.
8. Perform predosing baseline audiovestibular testing, laboratory testing, and physical examination as required by protocol.

##### 5.3.2.2 Intraoperative Procedures

9. Ensure surgeons are experienced in the procedure and have undergone training and validation specifically in the administration of the particular product being administered. Most often this will involve a round window approach to the inner ear, with or without a fenestration in the labyrinthine portion of the inner ear or the stapes footplate.
10. Follow exact handling specifications for the gene therapy product from the manufacturer, including storage and dispensing from pharmacy, transport to the operating room, and disposal and sterilization afterwards of any equipment in contact with products.
11. Use standard protocols for anesthesia relevant to the institution. Antibiotics are recommended, and intraoperative steroids are advised.

##### 5.3.2.3 Postsurgical Monitoring

12. Administer postsurgical steroids for a period of several days to minimize inflammatory reactions to viral and gene therapy products.
13. Monitor postoperative auditory and vestibular function using age-appropriate testing, strictly following protocol guidelines.
14. Conduct minimum safety evaluations, including at least:
  - Regular physical examination.
  - Tests for anti-viral antibodies.
  - Viral vector shedding assessment.
  - Routine blood analysis for hematology, biochemistry, and liver function tests.
  - Urinalysis.
15. Report adverse events and serious adverse events fully and expeditiously to the sponsors, ethics committees, and the institution, with full documentation on severity, causality, expectedness, and if it is a serious dose-limiting toxicity or drug reaction, as well as full documentation of management of the patient.
16. Ensure long-term follow-up. A minimum of 5 years of follow-up is recommended.

(See Appendix 3: Toolkit; Table 24 .)

(See Appendix 4: Clinical Checklist 11.)

## 6. Ongoing Care and Support

**Table:** 

After assessment and interventions for the management of hearing loss, follow-up sessions are essential for: • Measuring outcomes and re-assessing treatment goals. • Exploring alternative interventions and screening for onward referral for cochlear implants or to other health professionals. • Ensuring appropriate ongoing care. Adults with severe and profound hearing loss require ongoing care and support throughout their lives, as their needs will change over time, as will the available treatment options to assist them.

### 6.1 Measurement of Client-Reported Outcomes and Assessment of Treatment Goals

The assessment of outcomes is a key part of evidence-based clinical practice: to assess the effectiveness of interventions, to enhance and monitor individual care, and to evaluate services. Currently, there is general agreement on the importance of measuring outcomes, but poor consensus about the most appropriate tools to use. There are few questionnaires developed specifically for those with severe and profound hearing loss. There are, however, several validated questionnaires that have been used with this population and shown to be sensitive to interventions. Since the initial publication of these guidelines in which evidence was extrapolated from research on adults with mild to moderate hearing loss, the level of available evidence to support recommendations for those with severe and profound hearing loss has improved markedly. There have been six high-quality reviews published since 2019.

Recommendations (see evidence Appendix 1: Ongoing Care and Support, 6; Section 6.1: Measurement of Client-Reported Outcomes and Assessment of Treatment Goals)

1. Client-reported outcome measures (PROMs) represent the most effective way of capturing comprehensive information about benefits of an intervention. Speech perception tests are also commonly used to monitor device benefit; however, these do not provide information about the real-world functioning of adults with severe and profound hearing loss.
2. An outcome questionnaire should be used to assess functional performance, to identify need for amplification review, to help assess if goals have been met, and to identify needs for further rehabilitation.
3. Outcome questionnaires should include measures in the hearing domain (i.e., hearing activities and participation) and measures of device benefit. In addition, measures in broader domains may also be appropriate depending on an individual's situation (e.g., measures of quality of life, loneliness, fatigue, work satisfaction, third-party disability). In addition, in the United States in 2025, the National Academies of Sciences, Engineering and Medicine (NASEM) Committee on Meaningful Outcome Measures in Adult Hearing Health Care published guidelines on outcomes for interventions for adults (see https://www.ncbi.nlm.nih.gov/books/NBK616586/ ). Their recommendations are not specific to severe and profound hearing loss and include support for measuring intervention outcomes in terms of hearing-related psychosocial health.
4. The chosen questionnaire should have proven reliability, validity, and sensitivity.

(See Appendix 3: Toolkit; Table 2 .)

### 6.2. Assessing the Need for Onward Referral

HCPs should ensure appropriate onward referrals are made to deliver best hearing outcomes.

Recommendations (see evidence Appendix 1: Ongoing Care and Support, 6; Section 6.2. Assessing the Need for Onward Referral)

1. HCPs should ensure they are aware of criteria for candidacy for cochlear implants and seek advice from their local cochlear implant service.
2. Aided speech performance should be regularly tested to monitor device benefit and can be important for assessing candidacy for cochlear implant referral. However, it should not be the only criterion for candidacy.
3. Onward referral to other agencies should be made at any stage of the rehabilitative journey to ensure wider support for those with severe and profound hearing loss.

(See Appendix 3; Toolkit: Table 4 .)

### 6.3 Ensuring Appropriate Ongoing Management

The management of severe and profound hearing loss is an ongoing process of continued hearing device optimization and maintenance, promotion of self-management strategies, provision of advice and support, and onward referrals where appropriate. HCPs need to continually develop their skills to optimally manage this complex group.

Recommendations (see evidence Appendix 1: Ongoing Care and Support, 6; Section 6.3: Ensuring Appropriate On-Going Management)

1. Clients with severe and profound hearing loss should have follow-up after an intervention to support them to optimally use their devices and manage listening environments. In general, this should be face-to-face, as phone use may be a challenge without lipreading cues. Visual online follow-up may be appropriate.
2. Clients with severe and profound hearing loss should have easy access to ongoing care and maintenance to ensure hearing aids are in good working order with well-fitting earmolds and frequent tubing changes.
3. Clients with severe and profound hearing loss should be directed to other sources of support and rehabilitative interventions.
4. Clients with severe and profound hearing loss should be seen for regular review at least every 3 years, or more frequently if hearing changes, to check hearing and optimize amplification.
5. Professionals involved in the care of clients with severe and profound hearing loss should continue to develop their skills and knowledge in the audiological management of this population.

(See Appendix 3: Toolkit; Tables 2 and 4 .)

(See Appendix 4: Clinical Checklist 12.)

## 7. Summary, Future Research Considerations, and Conclusions

### 7.1 Future Directions

Despite advances in technology, assessment methods, and models of care, substantial gaps remain in the evidence base for adults with severe and profound hearing loss. Addressing these gaps is essential for improving diagnostic accuracy, optimizing treatment pathways, and ensuring equitable access to care. Priority areas for future research span assessment, technology, rehabilitation, and service delivery, reflecting the breadth of unanswered questions that continue to limit best practice. Strengthening the evidence base will be critical for improving diagnostic precision, enhancing treatment outcomes, reducing inequities in access, and ensuring that future guidelines are grounded in robust, population–specific data. Progress in this field will depend on sustained collaboration between individuals with severe and profound hearing loss, researchers, HCPs, Deaf communities, industry partners, and policymakers, working collectively to advance knowledge and improve care for this diverse population.

#### 7.1.1 Sign Language Users

There is a significant lack of research addressing the needs of Deaf sign language users within audiology. Priority areas include:

- Development of outcome measures that are valid for sign language users.
- Evaluation of interventions delivered in sign language, including counseling, rehabilitation, and technology–related education.
- Investigation of how cultural and linguistic identity influences device uptake, patterns of use, and long–term adjustment.
- Assessment of the effectiveness of audiologist training in Deaf awareness, sign language, and cultural competence at organizational, structural, and interpersonal levels.

Research in these areas is essential to ensure that audiological services are culturally responsive and accessible to Deaf communities.

#### 7.1.2 Assessment

##### 7.1.2.1 Understanding Variability in Speech Perception

Although existing evidence demonstrates that speech understanding and speech–in–noise performance are highly variable among adults with severe and profound hearing loss, the underlying drivers of this variability remain poorly understood. Factors such as auditory neuropathy, temporal processing deficits, cognitive load, and linguistic experience may contribute, yet these mechanisms are not easily assessed in routine clinical practice. Research is needed to develop clinically feasible tools that can better characterize the sources of individual variability and guide personalized intervention strategies.

##### 7.1.2.2 Cochlear Implant Candidacy and Access

Cochlear implants are well–established and effective treatments for severe and profound hearing loss. However, candidacy guidance varies across regions and services, and recent evidence highlights disparities in access linked to socioeconomic status and other demographic factors. Further research is required to understand the barriers faced by underserved groups, including structural, cultural, financial, and informational barriers. This work is essential to support equitable access and to inform policy and service redesign.

##### 7.1.2.3 Objective Identification of Cochlear Dead Regions

A major research gap concerns the development of reliable, objective measures for identifying cochlear dead regions. Several promising approaches—such as the auditory steady–state response (Markessis et al, 2009) and the acoustic change complex (Kang et al, 2018; Schelenz et al, 2025)—have been proposed, but none have been sufficiently validated in populations with hearing loss. No standardized clinical protocols or normative datasets currently exist. Future research should include:

- Studies involving adults with confirmed dead regions.
- Development of frequency–specific normative data.
- Evaluation of test–retest reliability and clinical feasibility.
- Creation of standardized protocols suitable for routine practice.

##### 7.1.2.4 Outcome Measures

Establishing robust objective measures would significantly enhance diagnostic accuracy and inform more precise amplification strategies.

#### 7.1.3 Prescribing and Fitting Hearing Aids

##### 7.1.3.1 Hearing Aid Technology for Severe and Profound Hearing Loss

At the time of writing, it was observed that there is a lack of research findings that directly address the use of hearing aids with advanced signal processing with individuals with severe or profound hearing loss. There have been many developments in hearing aids with features designed to reduce the negative effects of background noise on speech and in acoustic environment detection, to name but two. Future studies could not only focus on speech discrimination and understanding but also on the reduction of listening effort and fatigue along with other assessments of quality of life. It is hoped that further developments in hearing aid technology will be directly tested with individuals with severe and profound hearing loss.

##### 7.1.3.2 Options for Music

Even though there is a lack of literature on the options to improve the perception of music for musicians and music enthusiasts with severe and profound loss, music should still be discussed by HCPs with their clients to discover if music is an important part of their lives. Music has many benefits, so a severe or profound hearing loss should not be a barrier to enjoying or making music. Principles can be used from research performed with milder losses to fit hearing aids, and from others, including specialists in the area of cochlear implants and musicology, to develop clinically appropriate options for individuals with severe and profound hearing loss. It is hoped that more work will be done to directly address the needs of individuals with severe and profound hearing loss for them to enjoy music in whichever way is best for them.

#### 7.1.4 Public Broadcast (Auracast) and Assistive Listening in Public Spaces

The introduction of Auracast and next–generation broadcast audio systems represents a major shift in public–accessibility technology. However, several research questions remain unanswered:

- Market penetration: Little is known about the real–world availability of Auracast transmitters in public venues or the proportion of hearing devices capable of receiving Auracast signals. Reliable market data are needed to guide clinical recommendations and inform public–health planning.
- Quality–of–life outcomes: Research should evaluate the psychosocial, communicative, and participation–related benefits of Auracast for adults with severe and profound hearing loss.
- Loudness, comfort, and fatigue: For individuals using high–gain amplification or implantable devices, studies are needed to examine loudness growth, compression effects, and the potential for auditory fatigue during prolonged use of broadcast audio systems.

These findings will be critical for ensuring safe, effective, and equitable implementation of Auracast technologies.

#### 7.1.5 Remote Fitting and Teleaudiology

Remote care is increasingly integrated into hearing healthcare, yet evidence specific to adults with severe and profound hearing loss remains limited. Research priorities include:

- High–quality randomized controlled trials evaluating remote initial fittings.
- Development of standardized verification protocols suitable for remote delivery.
- Inclusion of speech–in–noise outcomes as primary endpoints.
- Evaluation of infrastructure, training, and workflow requirements for safe and effective implementation.
- Exploration of AI–driven personalization and its potential to support complex fittings.

These studies will help determine when remote care is appropriate, safe, and effective for this population.

#### 7.1.6 Tinnitus in Severe and Profound Hearing Loss

Although research on tinnitus management has expanded, there remains a paucity of studies specifically addressing tinnitus in adults with severe and profound hearing loss. Many current recommendations assume that tinnitus mechanisms and treatment responses are similar across degrees of hearing loss once communication barriers are accounted for. However, this assumption has not been empirically tested. Future research should focus on:

- Characterizing tinnitus profiles unique to this population.
- Evaluating the effectiveness of tinnitus interventions (including psychological therapies, sound–based therapies, and cochlear implantation) specifically in severe/profound loss.
- Understanding the interaction between tinnitus, communication challenges, and psychosocial outcomes.

Such work is essential to refine clinical guidance and ensure that tinnitus care is appropriately tailored.

### 7.2 Summary and Conclusion

Adults with severe and profound hearing loss represent a highly heterogeneous population whose needs extend far beyond the scope of conventional audiological practice. These updated guidelines outline the additional considerations, adaptations, and specialist competencies required to deliver equitable, person–centered, and evidence–based care for this group. Across assessment, technology selection, rehabilitation, and long–term management, the overarching message is clear: optimal outcomes depend on a holistic, individualized, and culturally responsive approach that recognizes the complexity of this population's communication, psychosocial, and medical needs.

A comprehensive assessment, encompassing communication goals, psychosocial impact, nonauditory factors, and detailed behavioral and objective audiological testing.forms the foundation of effective care. Treatment planning must be collaborative, transparent, and grounded in shared decision–making. Hearing aids remain a central intervention, but they must be fitted with precision, verified rigorously, and supported by adjunctive technologies such as remote microphones, assistive listening devices, and environmental adaptations. For many adults, hearing aids alone will not provide sufficient benefit; therefore, HCPs must be confident in initiating timely discussions about cochlear implants and other implantable technologies, ensuring that clients understand their full range of options.

Rehabilitation for this population extends well beyond technology. Psychosocial support, communication training, family involvement, and community–based interventions are essential components of best practice. The guidelines emphasize the importance of addressing third–party disability, supporting communication partners, and recognizing the cultural and linguistic identities of Deaf sign language users. For these individuals, culturally competent care, accessible communication, and respect for Deaf culture are fundamental to effective audiological practice.

Emerging developments (including remote care models, Auracast and other wireless technologies, and advances in genetic testing and gene therapy) are reshaping the landscape of hearing healthcare. These innovations offer new opportunities but also require careful evaluation, robust safety frameworks, and ongoing professional development to ensure safe and effective implementation.

Finally, high–quality care requires ongoing professional training, reflective practice, and service–level commitment. HCPs should have access to continuing education, shared learning networks, and resources that support the management of this complex population (particularly in services where caseloads are small). As these guidelines are implemented, client feedback should be actively sought and used to refine practice, ensuring that services remain responsive, accessible, and person–centered.

In conclusion, these guidelines provide a comprehensive framework for best practice in the audiological management of adults with severe and profound hearing loss. By integrating evidence–based recommendations with expert consensus, they aim to standardize care, improve outcomes, and support HCPs in delivering high–quality, culturally competent, and future–focused hearing healthcare. Continued research, innovation, and professional development will be essential to advance the field and meet the evolving needs of this diverse population.

### 7.3 Declaration of Interests from the Authors

**Table:** 

Member (role)	Position and institute(s)/aﬃliations	Clinical ﬁeld/research area of interest	Memberships and conﬂicts of interest
Laura Turton, MSc (lead author and editor)	Clinical Scientist (Audiology) and Head of Audiology, NHS Tayside, United Kingdom	Clinical lead for an NHS audiology service which also includes a specialist service for management of adults with a severe and profound hearing loss. Special interests include person-centered care, tinnitus and hyperacusis management, and management of clients with a severe and profound hearing loss who do not proceed with cochlear implantation	Memberships—Principal Fellow of the British Society of Audiology (BSA) Board Director of the British Academy of Audiology Chair of the Clinical Reference Group for the Scottish Cochlear Implant Group Conflicts—No competing interests
Barbra H. B. Timmer, PhD (author and co-editor)	School of Health and Rehabilitation Sciences, The University of Queensland, Brisbane, Australia; Sonova AG, Stäfa, Switzerland	Research interests include the real-world challenges and audiological outcomes for adults with hearing impairment and improving rehabilitative audiology clinical practice	Memberships—Board Director and Member of Audiology Australia Member of the International Society of Audiology Executive Board Member of the International Society of Audiology Member of the Phonak Expert Circle on Family-Centered Hearing Care Conflicts—Employed by Sonova AG
Neil S. Hockley, MSc (author and co-editor)	Manager of Audiology, Phonak, Sonova AG, Stäfa, Switzerland	Maintaining and promoting scientific and clinical knowledge base for individuals with severe and profound hearing loss within the hearing aid industry. Develop and evaluate technology with clear benefits for individuals with severe and profound hearing loss. Initiate external studies with institutions worldwide	Memberships—Member of Speech-Language and Audiology Canada Member of the British Society of Audiology Member of the Canadian Academy of Audiology Conflicts—Employed by Sonova AG in Stäfa, Switzerland
Steven J. Aiken, PhD (author)	Associate Professor, Dalhousie University, Halifay, Nova Scotia, Canada	Research focused on assessing auditory function at the level of the brainstem and cortex using evoked responses, particularly in relation to complex (but functionally important) sounds such as speech and music. Core interest in how these objective electrophysiological measures relate to speech sound encoding and speech intelligibility, and how both are affected by hearing aid processing	Memberships Canadian Academy of Audiology Acoustical Society of America Association for Research in Otolaryngology Conflicts—No competing interests
Manohar Bance, M.B., F.R.C.S.(C) (author)	Professor of Otology and Skull Base Surgery, University of Cambridge	Surgeon-Scientist working in hearing and balance disorders and diseases of the ear. Runs an active clinical practice at a very large tertiary care otology centered, as well as a lab dedicated to these disorders, focusing on several areas currently, including optimizing the machine-neural interface for cochlear implants using in vivo recordings and bioprinted and instrumented cochlear models, site of lesion tests for hearing loss for intelligent inner ear guided regeneration therapies, cochlea-on-a-chip models, and novel high technology rehabilitation strategies for balance disorders	Memberships Fellow of the Royal College of Surgeons Fellow of the Royal College of Surgeons of Canada American Academy of Otolaryngology Head and Neck Surgery (Board Certified) Subspeciality Boards in Neurotology in American Academy of Otolaryngology Head and Neck Surgery Royal Society of Medicine British Society of Otology American Otology Society (by election and thesis) American Neurotology Society (by election) Triological Society (by election and thesis) Polizter International Ear Surgery Society (Board Member) European Academy of Otology and Neurotology Editorial Board: Otology and Neurotology Conflicts—Advisor: Camgene
Judith Bird, MSc (author)	Consultant Clinical Scientist and Head of Audiology and Emmeline Centre for Hearing Implants, Cambridge University Hospital NHS Foundation Trust, United Kingdom	Clinical lead of a tertiary audiology service that includes a long-standing specialist service for adults with severe and profound hearing loss. Special interests include adult hearing loss, optimizing hearing rehabilitation, and smoothing access to cochlear implants	Memberships—Senior Fellow of the British Society of Audiology Member of the British Academy of Audiology Member of the British Cochlear Implant Group Conflicts—No competing interests
Katie Ekberg, PhD (author)	College of Nursing and Health Sciences, Flinders University, Australia	Research on psychosocial issues related to hearing loss, client and family-centered hearing care, and real-world social interactions of people with hearing loss and their communication partners	Memberships—No memberships Conflicts—No competing interests
Louise HicksonPhD (author)	Emeritus Professor, School of Health and Rehabilitation Sciences, The University of Queensland, Australia	Professor of Audiology with broad research interests and expertise on audiological rehabilitation and the effects of hearing loss on people's everyday lives and the development of strategies and interventions that improve the uptake and outcomes of hearing rehabilitation. Extensive experience in the use of outcomes measures in audiology, and that includes measuring outcomes for families as well as children and adults with hearing loss	Memberships—Life Member of Audiology Australia Executive Board Member of the International Society of Audiology Chair of the Phonak Expert Circle on Family-Centered Hearing Care Conflicts—No competing interests
Celia Hulme, PhD (author)	University of Manchester	Research focuses on health services and social care for Deaf communities, with particular emphasis on cultural competence, sign language access, and equity in healthcare. Research on sign language users' experiences of audiology services and the feasibility of making hearing aid clinics more culturally appropriate through cultural competence training and system-level change	Memberships—Nil Conflicts—No competing interests
Anja Kurz, PhD (author)	Head of Audiology and Technical Director at the Comprehensive Hearing Center, University Hospital Wuerzburg, Germany	Applied clinical care research in hearing-impaired children and adults fitted with hearing implants	Memberships—German Society of Audiology, HEARRING member, Member of International Society of Audiology Conflicts—Audiology Advisory Board member: Advanced Bionics, MED-EL
Faheema Mahomed-Asmail, PhD (author)	Department of Speech-Language Pathology and Audiology, University of Pretoria, South Africa	Research driven by a transdisciplinary approach, focusing on strengthening systems and skills to enhance the quality and accessibility of hearing healthcare, particularly in low- and middle-income settings. This work centers on three key areas: improving the quality of hearing health through person-centered care, fostering interprofessional collaboration to optimize service delivery, and leveraging innovative solutions to expand access to hearing services	Memberships—Full Member, International Society of Audiology Council Member: South African Speech-Language-Hearing Association Contributing Member: Coalition for Global Hearing Health hearing screening working group Member of AfriPen Conflicts—No competing interests
Min Cheol Roh, MAud (author)	Sonova Communications AG, Murten, Switzerland	Clinical research into audiological performance of remote microphone technology and wireless communication	Memberships—Member MNZAS (New Zealand Audiological Society) Conflicts—Employed by Sonova Communications AG
Nerina Scarinci, PhD (author)	Associate Professor, Head of Speech Pathology, School of Health and Rehabilitation Sciences, The University of Queensland, Australia	Research on re/habilitation of children and adults with hearing impairment and their family members, client and family-centered care, best practice service delivery	Memberships—Member of Speech Pathology Australia Associate Editor, International Journal of Audiology Conflicts—Received a grant from Sonova to investigate perspectives of adults with hearing impairment, their family members and HCPs about remote microphone technology
Grant D. Searchfield, PhD (author)	Eisdell Moore Centre, Audiology, The School of Population Health, Faculty of Medical and Health Sciences, The University of Auckland, New Zealand TrueSilence Therapeutics Inc., United States	Research centers on auditory neuroscience and clinical audiology, with a particular focus on tinnitus pathophysiology and evidence-based therapeutic strategies. It aims to advance understanding of neural mechanisms underlying tinnitus and develop innovative interventions to optimize clinical outcomes	Memberships—MNZAS Member of the New Zealand Audiological Society Conflicts—Chief Scientific Officer, True Silence Therapeutics, A tinnitus therapy company.
Pamela Souza, PhD (author)	Communication Sciences and Disorders and Knowles Hearing Center, Northwestern University, United States	Research and clinical teaching in severe and profound hearing loss and variability of outcomes, hearing aid features, and processing	Memberships—American Academy of Audiology American Speech-Language-Hearing Association Acoustical Society of America American Auditory Society Hearing Loss Association of America Conflicts—Received a grant from Sonova to investigate client factors that affect benefit with a digital remote microphone
De Wet Swanepoel, PhD (author)	Department of Speech-Language Pathology and Audiology, University of Pretoria, South Africa Virtual Hearing Lab, Collaborative initiative between University of Colorado School of Medicine, Aurora, and University of Pretoria, South Africa Department of Otolaryngology–Head and Neck Surgery, University of Colorado School of Medicine, Aurora	Research focused on innovative digital solutions and service-delivery models for more accessible quality hearing care	Memberships—Member of International Society of Audiology Member of International Collegium of Rehabilitative Audiology (ICRA) Editor-in-chief, International Journal of Audiology Conflicts—Equity and consulting for LXE Hearing (previously hearX Group)
Linda M. Thibodeau, PhD (author)	Professor in Doctorate of Audiology Program, Behavioral and Brain Sciences, University of Texas at Dallas, Callier Center for Communication Disorders	Research and clinical teaching regarding services for those with severe and profound hearing loss across the lifespan, group auditory rehabilitation, and benefits of remote microphone technology measured in real-world and laboratory settings	Memberships—Member of the American Academy of Audiology Member of the American Speech, Language, and Hearing Member of the Educational Audiology Association Member of the Academy of Rehabilitative Audiology Member of the American Cochlear Implant Alliance Member of the American Auditory Society Conflicts—Consultant for Phonak

### 7.4 Date for Review of Guidelines

These guidelines will be reviewed 5 years from publication in 2031. Revision is planned for 2030.

**Appendix 1:** Evidence: summary tables of evidence applicable to each recommendation (from first and second editions)

First edition evidence—key						
Recommendation number	Evidence	Source (reference)	Level of evidence	Grade	EF/EV	MM/P/CI
	Statement from reference	Reference	1. Systematic reviews and meta-analyses of randomized controlled trials 2. Randomized controlled trials 3. Nonrandomized intervention studies 4. Descriptive studies (cross-sectional surveys, cohort studies, case-control designs) 5. Case studies 6. Expert opinion	A = Consistent level 1 or 2 studies B = Consistent level 3 or 4 studies or extrapolations from level 1 or 2 studies C = Level 5 studies or extrapolations from level 3 and 4 studies D = Level 6 evidence or troubling inconsistencies or inconclusive studies at any level	Efficacy (EF) is evidence measured under “laboratory or ideal” conditions Effectiveness (EV) is evidence measured in the “real” world	Evidence given for Mild-to-moderate hearing loss = (MM) Pediatrics = (P) Cochlear implants = (CI)
**Second edition evidence—key** The QuADS tool contains 13 criteria; each criterion was given a score between 0 (lowest) and 3 (highest). The tool therefore has a maximum possible score of 39. The QuADS criteria are: 1. Theoretical or conceptual underpinning to the research 2. Statement of research aim/s 3. Clear description of research setting and target population 4. The study design is appropriate to address the stated research aim/s 5. Appropriate sampling to address the research aim/s 6. Rationale for choice of data collection tool/s 7. The format and content of the data collection tool is appropriate to address the stated research aim/s 8. Description of data collection procedure 9. Recruitment data provided 10. Justification for analytic method selected 11. The method of analysis was appropriate to answer the research aim/s 12. Evidence that the research stakeholders have been considered in research design or conduct 13. Strengths and limitations critically discussed						

Supporting Accessibility and Deaf Awareness 1.0

### 1.1 Accessibility

**Table:** Second Edition Evidence

Recommendations	Source (reference)	QUADS criteria													Total
		1	2	3	4	5	6	7	8	9	10	11	12	13	
1–8	BSA Practice Guidance (2026)	2	3	NA	NA	NA	2	1	1	2	3	1	3	3	21
2, 4, 7, 8	Green & Pope (2001)	2	3	3	2	3	3	2	3	3	0	1	3	3	31
1–8	Hulme et al (2021)	3	3	3	3	3	3	3	3	3	3	3	3	3	39
1–8	Hulme et al (2023)	3	3	3	2	3	3	3	3	3	3	2	3	3	37
1–8	Hulme et al (2024)	2	3	3	2	3	3	2	2	3	3	2	3	3	34
1, 4, 7, 8	Jama et al (2020)	1	3	2	2	2	2	1	2	1	0	0	2	2	20
2, 3, 7	McKee et al (2011)	2	3	3	2	3	3	1	3	3	3	2	3	3	34
1–5, 8	McKee et al (2022)	3	3	NA	NA	NA	NA	1	1	3	3	1	3	3	21
1, 4, 7	Mormer et al (2017)	3	3	NA	NA	NA	NA	1	1	3	3	1	3	3	21
1–8	Parmar et al (2025)	2	3	3	2	3	3	3	2	3	3	2	3	3	35
2, 3, 5, 7	Stevens et al (2019)	3	2	1	3	2	2	3	2	0	0	3	1	3	25

### 1.2 Deaf Awareness

**Table:** Second Edition Evidence

Recommendations	Source (reference)	QUADS criteria													Total
		1	2	3	4	5	6	7	8	9	10	11	12	13	
1–9	BSA Practice Guidance (2026)	2	3	NA	NA	NA	2	1	1	2	3	1	3	3	21
3, 6, 8, 9	Green & Pope (2001)	2	3	3	2	3	3	2	3	3	0	1	3	3	31
1, 3–6, 8, 9	Hulme et al (2021)	3	3	3	3	3	3	3	3	3	3	3	3	3	39
1, 3, 4, 6, 8, 9	Hulme et al (2023)	3	3	3	2	3	3	3	3	3	3	2	3	3	37
1, 3–6, 8, 9	Hulme et al (2024)	2	3	3	2	3	3	2	2	3	3	2	3	3	34
2–4, 9	Jama et al (2020)	1	3	2	2	2	2	1	2	1	0	0	2	2	20
6, 8, 9	McKee et al (2011)	2	3	3	2	3	3	1	3	3	3	2	3	3	34
6–9	McKee et al (2022)	3	3	NA	NA	NA	NA	1	1	3	3	1	3	3	21
3, 8, 9	Mormer et al (2017)	3	3	NA	NA	NA	NA	1	1	3	3	1	3	3	21
1–9	Parmar et al (2025)	2	3	3	2	3	3	3	2	3	3	2	3	3	35
4, 5, 7–9	Stevens et al (2019)	3	2	1	3	2	2	3	2	0	0	3	1	3	25

Sign Language 2.0

### 2.1 Support and Integration of the Audiological Management of Adults with Severe and Profound Hearing Loss Who Use Sign Language

**Table:** Second Edition Evidence

Recommendation	Source (reference)	QUADS criteria													Total
		1	2	3	4	5	6	7	8	9	10	11	12	13	
2, 3, 5	Hulme et al, 2021	3	2	NA	2	3	2	3	3	3	3	3	3	3	33
1, 2, 6, 7	Hulme et al, 2022	3	3	NA	2	3	2	3	3	3	3	3	3	3	34
1–8	Hulme et al, 2023	3	3	NA	3	3	2	3	3	3	3	3	3	3	35
1–8	Hulme et al, 2026 (in press)	3	3	3	3	3	2	3	3	3	3	3	3	3	38
1–7	Rogers et al, 2024	3	3	3	3	3	3	3	3	3	3	3	3	3	39
3, 4, 6	McKee et al, 2011	3	3	NA	2	2	2	3	2	2	2	3	2	2	28
4	Kuenberg et al, 2016	3	1	1	1	2	1	2	3	2	2	3	3	2	26
3, 7	Parmar et al, 2025	3	3	NA	3	3	2	3	3	2	3	3	3	3	34
2, 5	Graham et al, 2025	3	3	3	3	3	1	3	3	2	2	3	2	3	34
2, 7	Gilmore et al, 2019	3	2	NA	2	3	2	3	3	3	3	3	2	3	32
1, 2, 8	WHO, 2021	3	3	3	0	3	3	3	2	3	3	3	2	1	32
3, 6	Wilson & Atcherson, 2017	2	0	0	0	0	1	1	3	2	3	3	3	2	20
3	Panning et al, 2021	3	2	NA	2	3	2	2	3	2	3	2	2	2	28
6	Cottrell et al, 2021	3	2	NA	2	3	1	2	3	2	3	2	2	2	27
1, 2, 8	BSA, 2025	3	3	NA	3	3	1	3	3	2	3	3	2	2	31

Assessment 3.0

### 3.1 Understanding Communication Needs and the Impact of Hearing Loss

**Table:** First Edition Evidence

Rec.	Evidence	Source (reference)	Level	Grade	EF/EV	MM/P/CI
1	Scoping review of self-report outcomes studies from 2000 to 2018 for pre- and postlingually deafened adults fitted with a unilateral CI	Boisvert et al (2020)	1	B	EF and EV	CI
1, 2	Review of patient-centered approaches to develop and maintain self-management of hearing loss and stresses the need to select outcome measures that assess broad domains	Ferguson et al (2019)	4	C	EF and EV	MM
2	Systematic review (65 studies) and meta-analyses (35 studies) of social outcomes for adults from hearing interventions—included severe to profound	Hori et al (2025)	1	B	EF	Included CI but not limited to that
2	Scoping review of autonomy, participation and work outcomes for postlingually deafened adults fitted with a unilateral CI	Nijmeijer et al (2020)	1	B	EF	CI
3	Third-party disability can be experienced by a family member, so they should be included in the assessment and rehabilitation of their family member	Meyer et al (2015) Preminger and Meeks (2012) Scarinci et al (2012)	3 3 3	B B B	EV EV EV	MM MM MM
3, 4	Shared goal setting and intervention can help to align communication and coping strategies for the person with severe and profound hearing loss and their communication partners	Preminger and Meeks (2012) Scarinci et al (2012) Ekberg (2015)	3 3 4	B B B	EV EV EF, EV	MM MM MM
4	Involving families in hearing rehabilitation can improve the client experience and their satisfaction	Ekberg et al (2023)	3	B	EF	MM

**Table:** Second Edition Evidence

Rec.	Source (reference)	QUADS criteria													Total
		1	2	3	4	5	6	7	8	9	10	11	12	13	
1	Boisvert et al (2020)	2	3	2	3	3	3	3	3	0	2	3	0	2	29
2	Hori et al (2025)	1	2	2	3	3	3	3	3	1	3	3	0	3	30
2	Nijmeijer et al (2020)	2	3	2	3	3	3	3	3	0	3	3	0	3	31
4	Ekberg et al (2023)	2	3	3	2	2	2	3	3	2	2	3	2	3	32

### 3.2 Nonauditory Needs Assessment

**Table:** First Edition Evidence

Rec.	Evidence	Source	Level	Grade	EF/EV	MM/P/CI
1A	Hearing loss is associated with cognitive decline, so should be proactively managed	Davies, 2014 Lin et al, 2013 Livingston et al, 2017, 2020	6 4 1 1	C B A A	– EF EF EF	
1B	There are greater levels of anxiety and depression among people with severe and profound hearing loss compared to the general population	Carlsson, 2015	4	B	EF	MM
1C	Physical	Davies, 2014 Davis, 2011	6 4	C C	– EF	
1E	Good dexterity and haptic sensitivity are associated with successful hearing aid use	Singh et al, 2013	3	C	EV	MM
1F	Dual sensory impairment has a significant impact on communication and well-being and can cause social isolation, depression, reduced independence, mortality and cognitive decline	Schneider, 2011	4	C	EF	CI

**Table:** Second Edition Evidence

Rec.	Source (reference)	QUADS criteria													Total
		1	2	3	4	5	6	7	8	9	10	11	12	13	
1A	Livingston et al, 2024	NA													NA
1A/4	Maidment et al, 2024	NA													NA
1A	Strutt et al, 2022	3	2	2	3	3	1	2	1	1	1	2	0	3	24
1B	Tan et al, 2024	2	2	2	3	2	1	2	1	2	2	3	0	3	25
1B/1D	Huang et al, 2024	3	3	2	3	1	2	3	1	2	2	2	0	2	26
1B	Doge et al, 2025	1	2	2	3	1	1	1	1	1	1	1	0	2	17
1B	Arayici et al, 2025	1	1	2	3	2	2	2	1	2	2	3	0	2	23
1D	Tonelli et al, 2024	1	3	2	3	3	3	3	3	1	2	3	0	3	30
1E	Kumar et al, 2000	1	2	1	1	1	2	1	1	1	1	1	0	2	15
1F	Klauke, 2023	1	3	0	0	0	0	0	0	0	0	0	0	2	6
2	Bott et al, 2019	3	3	3	3	3	2	2	2	2	2	3	0	1	29
2	Littlejohn et al, 2021	3	3	0	2	0	3	0	2	0	0	2	3	3	21
2	Dawes et al, 2022	NA													NA
3	Dawes et al, 2023	2	3	3	3	3	3	3	2	2	2	3	0	2	31

### 3.3 Diagnostic Assessment with Behavioral Testing

**Table:** First Edition Evidence

Rec.	Evidence	Source	Level	Grade	EF/EV	MM/P/CI
1, 2	Sudden hearing loss requires prompt investigation. The prevalence of tinnitus with profound hearing loss is between 67 and 100% in cochlear implant candidates. People with severe and profound hearing loss demonstrate significant tinnitus handicap with negative impact on quality of life and are candidates for specific tinnitus therapy	Chandrasekhar et al, 2019 Olze et al, 2011 Andersson et al, 2009 Kompis et al, 2012 Olze et al, 2011 Carlsson et al, 2015	1 3 4 3 3 4	A B B BBB	EV EV EVEVEV	CI CI CI CI CI
3	People with severe and profound hearing loss have a variety of loudness growth patterns that need to be managed for greater satisfaction with amplification	Gottermeier and De Filippo, 2018	3	C	EV	–
4a	Speech recognition (in quiet and in noise) is highly variable in individuals with severe hearing loss, and results can guide counseling about expectations	Souza and Hoover, 2018	4	B	EV	
4b	Speech testing is an indicator in cochlear implant candidacy	Holder, 2018	3	C	EV	
6	Prevalence of cochlear dead regions varies from 21 to 76% in studies. Most studies indicate that ordinary people with dead regions still benefit from high frequency amplification, but benefit may be limited where there are extensive dead regions	Aazh & Moore, 2007 Souza & Hoover, 2018 Cox et al, 2011 Cox et al, 2012 Kluk & Moore, 2005 Moore et al, 2000	3 4 3 3 3 3	B B B B B B	EV EV EV EF, EV EV EV	– – – – –

**Table:** Second Edition Evidence

Rec.	Source (reference)	QUADS criteria													Total
		1	2	3	4	5	6	7	8	9	10	11	12	13	
4a	Smith et al, 2024	2	2	1	3	2	2	2	1	2	1	1	0	3	22
4a	Salvago et al, 2024	3	1	2	1	1	1	1	1	2	0	3	0	3	19
4b	Hoppe et al, 2021	1	3	1	3	2	2	2	1	2	1	1	0	3	22
5	Fitzgerald et al, 2024	3	1	1	3	1	2	3	3	1	1	1	0	1	21
5	Zaar et 2024	3	2	1	3	0	2	3	3	2	0	1	0	0	20
6	Pepler et al, 2016	3	3	3	2	3	2	2	3	2	2	2	0	3	30
6	Shrivasta et al, 2020	2	1	3	2	0	2	3	3	3	1	1	0	3	24

### 3.4 Diagnostic Assessment with Objective Testing

**Table:** Second Edition Evidence

Rec.	Source (reference)	QUADS criteria													Total
		1	2	3	4	5	6	7	8	9	10	11	12	13	
1	Sogebi et al, 2022	3	3	3	3	3	3	3	3	3	1	3	1	3	35
1	Kaya et al (2016)	3	3	3	3	3	3	3	3	3	1	3	1	1	33
1	Habsi et al (2022)	3	3	3	3	2	3	3	3	3	1	3	1	3	34
2	Berlin et al (2003)	3	3	2	3	2	3	3	3	1	0	3	2	2	30
3	Starr et al (2001)	3	3	3	3	2	3	3	3	3	3	3	1	3	36
3	Wu et al (2022)	3	3	3	3	2	3	3	2	3	3	3	1	2	34
3	Chaudhry et al (2020)	3	3	3	3	3	3	3	3	3	3	3	0	3	36
3	Santarelli et al (2015a)	3	3	3	3	2	3	3	3	3	3	3	1	2	35
3	Santarelli et al (2015b)	3	3	3	3	2	3	3	3	3	3	3	1	2	35
4	Luiz et al (2024)	3	3	3	3	2	3	3	3	2	2	3	1	2	33
4	Swanepoel et al (2004)	3	3	3	3	2	3	3	3	3	2	3	1	3	35
4	de Bressieux et al (2025)	3	3	3	3	2	3	3	3	3	3	3	0	3	35
4	Eder et al (2020)	3	3	3	3	2	3	3	3	3	3	3	1	3	36
4	Dabbous et al (2020)	3	3	3	3	3	3	3	3	3	3	3	2	2	37

### 3.5 Developing a Comprehensive Treatment Plan

**Table:** First Edition Evidence

Rec.	Evidence	Source	Level	Grade	EF/EV	MM/P/CI
1/2	Care should be tailored to meet the specific needs of the individual, recognising they are the expert in their own hearing.	Manchaia et al, 2011	4	C	EV	MM
3	There should be frequent communication and personal contact with the hearing care professional, and discussion of expectations should aid adaptation to new amplification.	Gottermeier & De Filippo 2018 (145)	3	C	EV	–

**Table:** Second Edition Evidence

Rec.	Source (reference)	QUADS criteria													Total
		1	2	3	4	5	6	7	8	9	10	11	12	13	
1/2/6	BSA Principles of Person-Centered Care in Adult Hearing Rehabilitation, 2024	NA													NA
4	Maidment et al, 2023	NA													NA
5	Swords et al, 2024	1	3	3	3	3	3	3	3	3	3	3	0	3	34

Technology 4.0

### 4.1 Prescribing and Fitting Hearing Aids

#### 4.1.1 Compression

**Table:** First Edition Evidence

Rec.	Evidence	Source (reference)	Level	Grade	EF/EV	MM/P/CI
1	Most clients with severe and profound loss reported better loudness comfort and overall satisfaction with WDRC compared to linear amplification with compression limiting	Barker et al (2001)	3	B	EV	–
		Kuk et al (2003)	3	B	EV	–
1	WDRC results in better speech intelligibility across a range of speech input levels, and particularly for soft speech, compared to linear amplification	Souza & Bishop (1999)	3	B	EF	
		Ringdahl et al (2000)	3	B	EV	
		Villchur (1987)	3	B	EF	
1	If the client was previously fit with linear amplification, a period of acclimatization may be necessary before realizing the maximum benefits of WDRC	Keidser et al (2007)	3	B	EV	MM
2	People with severe loss prefer lower compression ratios over higher compression ratios. In at least one study, this was attributed to better preservation of low-frequency prosodic cues when lower compression ratios were used	Barker et al (2001)	3	B	EV	–
		Keidser et al (2007)	3	B	EV	–
2	Increasing speech audibility via use of high compression ratios did not improve speech intelligibility in people with severe and profound loss. This was attributed to the negative effect of distorting speech amplitude variations, or to the lesser contribution of information in speech “valleys” to speech intelligibility	DeGennaro et al (1986)	5	C	EF	–
		Drullman & Smoorenburg (1997)	3	C	EF	–
3	For most people with severe loss fit with slow WDRC and low compression ratios, using more than nine compression channels is not expected to improve target match or predicted speech audibility. More than nine channels may be necessary to achieve best fit to target and audibility for cookie bite audiograms	Woods et al (2006)	4	C	EF	–
3	A large number of compression channels may smooth vowel spectra and affect vowel identification. This is more likely to occur when a large number of channels is combined with high compression ratios	Souza et al (2012)	3	C	EF	–
		Shen et al (2018)	4	C	EF	
4	A majority of people with severe and profound loss performed worse with fast-acting WDRC than linear amplification	Boothroyd (1990)	3	B	EF	–
		Souza et al (2005)	3	B	EF	–
4	People with severe loss made more consonant manner confusions when using fast-acting WDRC than with slow-acting WDRC	Boothroyd et al (1988)	3	B	EF	–
4	The negative effect of fast-acting WDRC was greatest for people with more hearing loss and/or with poor spectral resolution. This was attributed to greater dependence on amplitude envelope cues, which were distorted by fast-acting WDRC	Davies-Venn & Souza (2014)	3	B	EF	–
		Davies-Venn et al (2009)	3	B	EF	–
4	People with severe loss performed better with compression designed to preserve amplitude envelope cues than with fast-acting WDRC	Weile et al (2011)	3	C	EF	–
5	Listeners who were long-time users of linear amplification reacted negatively to WDRC. Complaints included insufficient loudness and more noticeable background noise. Helpful strategies included adjusting acclimatizing to frequency-gain response prior to acclimatizing to compression and having the opportunity to compare different amounts of compression stored as different hearing aid memories	Convery et al (2008)	3	B	EV	–

**Table:** Second Edition Evidence

Rec.	Source (reference)	QUADS criteria													Total
		1	2	3	4	5	6	7	8	9	10	11	12	13	
6	Huang et al, 2025	3	3	3	3	2	3	3	3	1	2	3	0	2	31
4	Chen et al, 2021	3	3	3	3	2	3	3	3	2	2	3	0	2	32
4	Serman et al, 2021	1	0	2	1	0	1	2	2	1	0	1	0	1	12

#### 4.1.2 Device Choices and Programs

**Table:** First Edition Evidence

Rec.	Evidence	Source (reference)	Level	Grade	EF/EV	MM/P/CI
1	Greater benefit was obtained with two than with one hearing aid, and those performance improvements were greatest for listeners with severe and profound hearing loss. Specifically, two hearing aids resulted in better sentence and word recognition, improved gross localization, and higher subjective ratings of spatialization	Ricketts et al (2019)	3	C	EF	–
1	Over a 10-y period, the decline in speech recognition scores in the unaided ear was about 10% for a group of listeners with severe and profound loss who were fit unilaterally. The decline in performance in the unaided ear was significantly greater than the decline in speech recognition scores in the aided ear	Lee et al (2019)	4	C	EV	–
2	Clients with severe and profound loss reported greater acceptance of noise level with fixed directionality compared to omnidirectional processing	Aghsoleimani et al (2018)	3	B	EF	–
2	Clients with severe and profound loss demonstrated improved signal-to-noise recognition with directional processing, especially at unfavorable input SNRs and when visual cues were provided	Ricketts & Hornsby (2006)	3	B	EF	–
2	Clients with severe and profound loss had average improved SNRs of 13 dB and reported improved listening comfort and higher satisfaction with fixed directional processing, compared to omnidirectional processing	Kuhnel et al (2001)	3	B	EV	–
2	Clients with severe and profound loss had improved speech recognition in noise with multiband adaptive directionality, compared to omnidirectional processing	Weile et al (2011)	3	C	EF	–
3	For listeners with a range of audiograms up to moderately severe high-frequency hearing loss, binaural beamforming directional processing resulted in better sentence recognition in noisy and reverberant environments compared to adaptive directionality	Picou et al (2014)	3	B	EF	–
		Picou & Ricketts (2019)	3	C	EF	–
5	The hearing care professional should consider client abilities in setting manual or automatic activation of the remote microphone system	Wolfe (2018)	3	B	EV	–
6	Passive feedback systems which reduce maximum available gain may restrict speech audibility, compared to active feedback systems	Chung (2004)	6	D	EF	–
7	Custom earmolds with appropriate venting can maintain hearing aid gain, minimize feedback, and relieve pressure	Killion (2003)	6	D	EF	–
8	Clients with severe loss had better speech recognition for telephone signals transmitted wirelessly to both ears, compared to telephone signals transmitted wirelessly or via telecoil to one ear	Picou & Ricketts (2013)	3	C	EF	–

**Table:** Second Edition Evidence

Rec.	Source (reference)	QUADS criteria													Total
		1	2	3	4	5	6	7	8	9	10	11	12	13	
2–4	Dong et al, 2024	3	3	2	3	3	3	3	2	1	2	3	0	3	31
1	Ziemska-Gorczyca et al, 2024	2	2	1	2	2	2	2	1	1	2	2	0	2	21
2, 3, 9	Voss et al, 2022	3	3	2	3	3	3	3	3	2	3	3	0	3	34
1	Ghiselli et al, 2024	3	3	2	2	1	2	2	2	2	2	3	0	2	26
1	Yadav et al, 2025	1	3	2	2	1	2	1	2	2	1	1	0	1	19
4	Diehl et al, 2023	2	3	1	3	2	2	3	2	1	2	3	0	2	26
2, 3	Yaz & Kara, 2025	3	3	2	3	3	3	3	3	2	2	3	0	3	33

#### 4.1.3 Frequency Lowering for Adults with Severe and Profound Hearing Loss

**Table:** First Edition Evidence

Rec.	Evidence	Source (reference)	Level	Grade	EF/EV	MM/P/CI
1	Clients with severe and profound loss had similar consonant recognition, better spondee-in-noise scores, and better vowel scores without frequency compression compared to with frequency compression	Perreau et al (2013)	3	C	EV	CI
1	Five of 11 clients with severe and profound loss preferred (broadband) frequency compression to no frequency compression. Note, however, that the frequency compression paradigm was quite different from those in current use	Sakamoto et al (2000)	3	D	EV	–
2	Among 10 clients with severe and profound loss who compared their own hearing aids without frequency lowering to frequency compression and to frequency transposition, there were smaller improvements in recognition and more clients experienced degraded recognition when using frequency transposition	Hotton & Bergeron (2017)	3	C	EV	–
2	An acoustic analysis conducted with example hearing aids fit to match NAL targets for a single severe loss audiogram indicated that frequency compression preserved vowel and consonant spectra better than frequency transposition. However, the same acoustic analysis suggested that frequency transposition—with its greater capability to move speech components to a low-frequency range—might be more suitable than frequency compression for clients with no usable hearing above 1–2 kHz	McDermott (2011)	4	C	EF	–
3	To ensure audibility of high-frequency phonemes avoid unnecessary distortion; experts recommend that frequency lowering be fit using real-ear verification and validated with appropriate test materials	Glista & Scollie (2018)	6	D	EF	–

**Table:** Second Edition Evidence

Rec.	Source (reference)	QUADS criteria													
		1	2	3	4	5	6	7	8	9	10	11	12	13	Total
2	Lee & Lee, 2024	2	3	2	3	2	2	2	2	2	2	3	0	2	27
4	Brennan & McCreery, 2021	3	3	3	3	2	3	3	3	3	3	3	0	3	35
1	Xu et al (2020)	3	3	3	3	2	3	3	3	1	3	3	0	3	33

#### 4.1.4 Prescriptions and Verification

**Table:** First Edition Evidence

Rec.	Evidence	Source (reference)	Level	Grade	EF/EV	MM/P/CI
1	Clients with mild and moderate hearing loss who were fit with a validated prescriptive procedure (NAL-NL1) reported greater hearing aid benefit compared to clients fit with “first fit” settings	Abrams et al (2012)	3	B	EV	MM
2	Hearing aids fit to NAL-NL targets and DSL (i/o) targets are likely to result in similar weighted audibility (SII values) for conversational and higher input levels	Ching et al (2015)	3	B	EF	MM
2	Clients with a range of hearing loss severity whose hearing aid fit was verified using real ear measures reported higher perceived benefit and greater handicap reduction compared to clients fit without real ear verification	Kochkin et al (2010)	4	C	EV	–
3	Clients fit with NAL-NL prescribed gain following amplification with nonprescribed gain (e.g., more low- and less high-frequency amplification than prescribed) were able to adjust to prescribed gain without significant changes in loudness comfort or sound quality	Convery & Keidser (2011)	3	B	EV	–
		Convery et al (2008)	3	B	EV	–
4	Frequency-gain and compression response should be verified using broadband signals. Use of pure-tone signals to verify compression response may result in gain adjustments that are different from those that would occur with speech inputs	Stelmachowicz (1991)	6	D	EF	–
5	Clients with conductive loss prefer significantly more gain than those with similar levels of sensorineural hearing loss	Berger (1980)	4	C	EV	–
		Johnson (2013)	6	D	EV	–
6	In clinical fits, most clients with identified dead regions showed either a small advantage or no effect of amplification in the frequency region of the dead region	Mackersie et al (2004)	3	B	EF	–
		Cox et al (2012)	3	B	EF, EV	
6	In rare cases of clients with extensive dead regions, provision of gain in the frequency region of the dead region resulted in degraded speech recognition	Vickers et al (2001)	3	B	EF	–

**Table:** Second Edition Evidence

Rec.	Source (reference)	QUADS criteria													Total
		1	2	3	4	5	6	7	8	9	10	11	12	13	
1, 2, 4	Engler et al, 2024	3	3	3	3	2	3	3	3	3	3	3	0	3	35
1, 3	Narayanan & Manjula, 2021	2	2	2	3	3	3	3	2	2	2	2	0	2	28

#### 4.1.5 Selecting Technology for Asymmetrical Severe and Profound Loss

**Table:** First Edition Evidence

Rec.	Evidence	Source (reference)	Level	Grade	EF/EV	MM/P/CI
1	73% of 82 clients with asymmetrical severe and profound loss who completed a trial with BiCROS devices chose to keep the BiCROS. However, the study lacked a control (monaural) condition, and no hearing aid history was reported	Hill et al (2006)	3	D	EV	–
1	14 clients with asymmetrical loss (moderate-to-severe in one ear and profound loss in the other ear) demonstrated significantly better speech in noise with BiCROS aids than with monaural amplification in the better ear. Specifically, SRTs improved by 3–4 dB and sentence recognition improved by about 10% when using the BiCROS	Del Dot et al (1992)	3	D	EF	–
1	There was no statistically significant improvement in speech in noise when using a BiCROS system, compared to monaural amplification in the better ear	Williams et al (2012)	3	C	EV	–
1	For 21 listeners with asymmetrical loss (mild to moderately severe in one ear and severe and profound loss in the other ear), there was no statistically significant improvement in speech in noise (HINT) threshold, regardless of level of digital noise reduction (none, mild, strong) when using a BiCROS system compared to no amplification. For the same listeners, subjective outcomes (APHAB) were improved when using the BiCROS system (compared to no amplification) over a 4-wk trial. During the trial, listeners were able to switch between different levels of digital noise reduction. The authors note that most did not switch and used the level of noise reduction (none, mild, strong) that had been randomly assigned to program 1	Oeding & Valente (2015)	3	D	EF	MM
1	Six listeners with asymmetrical loss (mild to moderately severe in one ear and severe and profound in the other ear) demonstrated better speech in noise with BiCROS aids than with monaural amplification in the better ear. Specifically, consonant recognition improved by about 10% when using the BiCROS	Kuk et al (2014)	3	D	EF	–
1	Nine participants with asymmetrical loss (severe and profound in one ear and mild or moderate loss in the other ear) who were fit with a BiCROS aid reported improved speech quality compared to use of a monaural hearing aid in the better ear	Kuk et al (2015)	3	B	EV	–
2	Listeners reported better speech recognition in noise, better sound quality, and greater ability to localize sound when using a BiCROS transmitter with a higher processing and adjustment capability (e.g., a higher number of compression channels) compared to older BiCROS devices with more limited adjustment capability. However, the study was not blinded, and participants were aware that they were comparing their current BiCROS to a newer BiCROS option	Williams et al (2012)	3	C	EV	–
2	BiCROS gain and frequency response should be adjusted to maintain the head-related transfer function and compensate for head shadow. In other words, adding a transmitter on the poorer ear should be acoustically transparent such that the desired frequency-gain response for the hearing aid fit to the better ear is maintained	Hayes et al (2005)	6	D	EF	–
2	The strength of the signal from the BiCROS transmitter—and thus the level of the received signal—depends on head size and on the physical position of each device on the ear	Hayes et al (2005)	6	D	EF	–
2	Nine participants with asymmetrical loss (severe and profound in one ear and mild or moderate loss in the other ear) who were fit with a BiCROS aid reported better speech recognition in noise when given the ability to turn off the transmitter microphone in cases of greater noise to the transmitter side	Kuk et al (2015)	3	B	EV	–
2	In diffuse noise, best speech in noise performance was obtained when both transmitter and receiver devices were configured with adaptive directionality. Specifically, sentence SRTs improved by 3 dB compared to both devices configured to be omnidirectional and by 5 dB compared to adaptive directionality on only the better ear. On average, configuring only a single device (either transmitter or receiver) with adaptive directionality minimally improved sentence SRT (≤1 dB)	Kuk et al (2014)	3	D	EF	–
2	Nineteen participants with asymmetrical loss (severe and profound in one ear and mild to moderate loss in the other ear) who were fit with a BiCROS aid demonstrated better speech recognition when both receiver and transmitter were configured with adaptive directionality, compared to adaptive directionality on only the better ear. Specifically, SRTs for HINT sentences improved by 2.6 dB with receiver adaptive directionality	Valente & Oeding (2015)	3	B	EF	–
New 3	The primary benefit expected for patients fitted with CROS/BiCROS technology is increased awareness of sound arising from their unaided side (i.e., reduction of the head shadow effect). Some clients may find it hard to understand how aiding their better ear could possibly address their listening difficulties. In this regard, probe-microphone systems can serve as a valuable educational tool by allowing the clinician to demonstrate the advantage to be expected from a CROS/BiCROS system for a patient with an unaided ear	Pumford (2005)	6	D	–	MM

**Table:** Second Edition Evidence

Rec.	Source (reference)	QUADS criteria													Total
		1	2	3	4	5	6	7	8	9	10	11	12	13	
1–3	Saleh et al, 2025	3	3	3	3	3	3	3	3	2	3	3	0	3	35
1	Takaki et al, 2024	3	3	2	3	2	2	3	3	3	2	3	0	2	31
1	de Matos et al, 2020	2	3	2	3	1	2	2	2	1	2	3	0	2	25
4	Lefeuvre et al, 2024	2	3	2	3	2	3	3	3	1	2	3	0	3	30

#### 4.1.6 Maximum Power Output and Threshold Shift

**Table:** First Edition Evidence

Rec.	Evidence	Source (reference)	Level	Grade	EF/EV	MM/P/CI
1	Calculated exposure levels indicate that clients with hearing loss exceeding 70 dB HL may be at risk of threshold elevation from high levels of amplified sound. The relative risk may be influenced by environmental sound levels, ear canal volume, manual volume control setting, and prescriptive procedure. The highest risk is likely to occur for clients fit with higher-gain prescriptions, small ear canal volume, and who are more frequently exposed to high levels of environmental sound	Ching et al (2013)	4	D	EF	–
		Humes & Bess (1981)	6	D	EV	–
		Johnson (2017)	6	D	EF	–
2	Verification of real-ear aided response for a 90 dB input using a pure tone can more accurately represent output levels for any narrow-band signals that the user experiences	Stelmachowicz (1991)	6	D	EF	–
3	Clients with moderate to profound loss preferred compression limiting over peak clipping, when using aids in their everyday environments	Savage et al (2006)	3	B	EV	–
4	Loudness discomfort is more likely to be associated with high-frequency output limits than with low-frequency output limits	Preminger et al (2001)	3	C	EF	–
4	It is expected that single-channel output limiting, where one control manages the entire range of frequencies, will result in limits to signal amplification across frequency. Multichannel output limiting will allow the fitter to customize MPO parameters to LDLs which vary across frequency	Taylor (2008)	6	D	EF	–
5	In children who adjusted their own volume control, approximately 15 dB of permanent threshold shift was attributed to adjustment of volume control above reserve gain. No such data exist for adult hearing aid wearers	Macrae (1991)	5	C	EV	–
		Macrae (1995)	5	C	EV	–

## Second Edition Evidence

No new evidence

## Considerations for Music

**Table:** Second Edition Evidence

Rec.	Source (reference)	QUADS criteria													Total
		1	2	3	4	5	6	7	8	9	10	11	12	13	
1–7	Chasin, 2022	NA													NA
2	Hake et al, 2025.	3	3	3	3	1	3	3	3	1	3	3	0	3	32
2	Amlani et al, 2014	2	3	0	2	1	2	2	3	0	3	3	0	1	22
3	Vaisberg et al, 2021.	3	3	3	3	1	3	3	3	1	3	3	2	3	34
4–7	Moore, 2016	NA													NA
7	Uys et al, 2016	2	3	3	3	1	2	3	3	1	2	3	1	2	29

### 5.1 Prescribing and Fitting Remote Microphones

#### 5.1.1 Considerations for Recommending and Managing Ongoing Use of Remote Microphone Systems

**Table:** First Edition Evidence

Rec.	Evidence	Source (reference)	Level	Grade	EF/EV	MM/P/CI
1–8	23 adults with hearing impairment, 7 key significant others of those adults, and 13 hearing care professionals were interviewed about their experiences with remote microphone systems. Five themes were identified: (1) With experience and clear expectations, clients, significant others and hearing care professionals believe in remote microphone systems and how they can make a difference, (2) the trial and decision-make process, (3) what happens when clients use remote microphone systems, (4) issues with the systems and technology, and (5) clients, significant others and hearing care professionals require ongoing support to use remote microphone systems.	Scarinci et al (2022)	4	B	EV	–
1, 5	12 adults wearing hearing aids (some with severe and profound hearing loss) demonstrated benefits of remote microphone systems in the laboratory. Participants identified challenges with using the systems in the real world, and authors advocated for the need for detailed instructions to achieve optimal outcomes	Boothroyd (2004)	3	B	EF/EV	–
1, 3	Real world evaluation of the use of remote microphone systems in 36 adult participants with severe and profound hearing loss. Positive improvements were evident for hearing conversation in noise, on the telephone, and hearing a speaker at a distance. Participants used remote microphone systems for a 6 wk trial period that included counseling and coaching, and all decided to continue use at the end of the trial	Chisolm et al (2007)	4	B	EV	–
1	12 adults with severe-to-profound hearing loss and wearing cochlear implants had improved speech perception in noise in a laboratory setting	De Ceulaer et al (2016)	4	B	EF	CI
1	15 adult cochlear implant users demonstrated improved listening to television in a laboratory setting	Fitzpatrick et al (2009)	4	B	EF	CI
1, 5	Evaluated real-world experiences of remote microphone systems in 14 adult cochlear implant users. Most common uses were (in order of priority): television, meeting, car, church. A number of technical, individual, social and environmental factors influenced use of the systems. Authors cited the need for additional counseling and instructions in order to achieve success	Fitzpatrick et al (2010)	4	B	EV	CI
1	Study aimed to develop a real-world questionnaire to evaluate benefits and difficulties associated with remote microphone systems. Trialled on 12 adult cochlear implant users	Fournier et al (2012)	5	B	EV	CI
1	Laboratory study comparing speech perception in noise with hearing aids including directional microphones and hearing aids coupled with remote microphone systems. Forty-six participants with mild sloping to severe hearing loss were included. Remote microphone systems were superior to hearing aids	Lewis et al (2004)	3	B	EF	MM
1, 3	14 adult cochlear implant users had speech perception in noise tests pre- and posttrial period with remote microphone systems. Benefits of remote microphone systems were evident in the laboratory tests; however, benefits were less consistently evident in self-report of real-world performance	Schafer et al (2013)	4	B	EF/EV	CI

**Table:** Second Edition Evidence

Rec.	Source (reference)	QUADS criteria													Total
		1	2	3	4	5	6	7	8	9	10	11	12	13	
1	Zanin et al (2025)	3	3	3	2	2	3	3	2	2	2	2	0	3	30
1	Ellag et al (2025)	3	2	2	2	2	2	3	2	1	2	2	0	2	25
1	Wesaarg et al (2020)	2	2	2	2	2	3	2	1	1	1	3	0	3	24
1	Chen et al (2021)	3	3	3	2	2	3	3	3	1	3	3	0	3	32
1, 3	Lazzerni et al (2023)	2	2	3	3	NA	3	2	3	NA	3	3	1	2	27
1	Dorman et al (2021)	3	3	3	3	2	3	3	3	2	3	3	3	3	37
1	Thibodeau et al (2024)	3	3	3	3	2	2	3	3	1	3	3	1	3	33
8	Scarinci et al (2022)	3	3	3	2	2	3	3	2	2	2	2	0	3	30

#### 5.1.2 Component Considerations

**Table:** First Edition Evidence

Rec.	Evidence	Source (reference)	Level	Grade	EF/EV	MM/P/CI
1	The system should provide benefit in challenging communications situations beyond that obtained with the local microphone system (hearing aid and/or cochlear implant) according to the individual's communication demands	Thibodeau (2004, 2010)	4	B	EV	MM
1	Should the user have frequent challenges in group settings, the ability to select directional pickup patterns of the RMT is optimal. This can provide up to 16% improvement in speech recognition in noise over RMT with fixed omnidirectional patterns	Thibodeau (2019a)	3	B	EF	MM, CI
2	If compatible with the personal device, the RMT should have the capability of hard-wired audio input connections to devices without Bluetooth and wireless connections to those devices such as smartphones with Bluetooth	Thibodeau (2007)	4	C	EV	–
3	The system should be comprised of the minimal number of components to facilitate troubleshooting and minimize repairs in bilateral/bimodal arrangements with consideration of financial constraints for the individual	Thibodeau (2019b)	4	B	EV	–
		AAA (2011)	6	D	EV	
3	Benefits with direct connection remote microphone systems can be achieved by cochlear implant users but are not as great as more expensive multi-component systems	Wolfe et al (2015c)	3	B	EV	CI
3	Adaptive digital remote microphone technology provided about 20% greater benefit than fixed-gain technology at higher noise levels	Wolfe (2018)	3	B	EV	
4	The charging options and battery life should meet the communication needs and lifestyle of the user	Thibodeau (2019b)	4	B	EV	–
5	The system should efficiently interface with other assistive technology that may be provided based on ADA requirements in employment and/or higher education settings	Thibodeau (2019b)	4	B	EV	–
		AAA (2011)	6	D	EV	–

**Table:** Second Edition Evidence

Rec.	Source (reference)	QUADS criteria													Total
		1	2	3	4	5	6	7	8	9	10	11	12	13	
1, 4, 5	Ellag et al (2025)	3	2	2	2	2	2	3	2	1	2	2	0	2	25
5	Dorman et al (2021)	3	3	3	3	2	3	3	3	2	3	3	3	3	37

#### 5.1.3 Considerations Regarding Remote Microphone Verification

**Table:** First Edition Evidence

Rec.	Evidence	Source (reference)	Level	Grade	EF/EV	MM/P/CI
1	If a hearing aid is part of the RMT, it should first be evaluated to ensure adequate function	ANSI S3.22	6	D	EF	–
2	If the remote microphone is interfaced with personal ear level technology, the output across the spectrum at the listener's ear when using the remote microphone is equivalent to the output when using the personal technology	AAA Guidelines	6	D	EF	–
2	Evaluation of electroacoustic output across remote microphone systems from four manufacturers with a single hearing aid revealed differences in frequency response	Salehi, Parsa, & Folkeard (2018)	6	D	EF	–
3	The RMT should produce minimal circuit noise	ANSI S3.47	6	D	EF	–
4	The RMT should produce minimal distortion	ANSI S3.47	6	D	EF	–
5	The RMT should match the frequency response of the personal hearing aid	AAA Guidelines	6	D	EF	–
5	There should be transparency between the output curves obtained with 65 dB SPL input for the hearing aid and the hearing aid plus the RMT	AAA Guidelines	6	D	EF	–
5	In general, the steps involve first placing the hearing aid in the test box to measure the output of the hearing aid alone, followed by placing the RM in the test box to measure the output of the combined hearing aid and RMT when each is tested with a 65 dB SPL complex signal input. The two output curves should be closely aligned, which will then result in the optimal SNR when RM receives the typical input of 80 dB SPL from the talker	AAA Guidelines	6	D	EF	–
5	If the two output curves are not similar, adjustments may be necessary in the hearing aid or receiver to compensate for the offset	Bondurant & Thibodeau (2011)	3	B	EF	–
6	Behavioral performance with the RMT should be significantly better than without it. The individual with the hearing aid combined with the RMT receiver is seated in the sound booth at 0 degrees azimuth, while the examiner with the RM is seated at the audiometer outside the booth. In general, the steps for the behavioral verification include obtaining the first score with the hearing aid/implant alone via live-voice presentation of the age-appropriate speech materials at 50 dB HL combined with 50 dB HL of competing noise, i.e., a 0 dB S/N. If this first score is not below 80%, the noise may be increased to create a more challenging SNR. The next condition is similar to the first measure except now the examiner has turned on the RM. The benefit is determined by comparing the score with the hearing aid/implant alone to the score obtained when the RMT was added	AAA Guidelines	6	D	EF	–
6	The average benefit for ten adults with hearing aids when tested using this protocol with FM technology was 34%	Thibodeau (2007)	3	B	EF	–
6	The average benefit for ten adults with hearing aids/cochlear implants when tested with digital modulation technology was 61%	Thibodeau (2019a)	3	B	EV	MM. CI
6	Use of remote microphone technology resulted in significant improvements in speech recognition in noise as great as 50% at higher noise levels	Wolfe et al (2015a,b, 2009)	3	B	EV	CI
6	Behavioral performance in real-world settings should be better with the RMT as reported by the user and/or communication partners on a self-assessment scale such as the TELEGRAM	Thibodeau (2004)	4	B	EV	MM
		Thibodeau (2007)	4	C	EV	–

**Table:** Second Edition Evidence

Rec.	Source (reference)	QUADS criteria													Total
		1	2	3	4	5	6	7	8	9	10	11	12	13	
2, 6	Zanin et al (2025)	3	3	3	2	2	3	3	2	2	2	2	0	3	30
6	Ellag et al (2025)	3	2	2	2	2	2	3	2	1	2	2	0	2	25
6	Wesaarg et al (2020)	2	2	2	2	2	3	2	1	1	1	3	0	3	24
6	Chen et al (2021)	3	3	3	2	2	3	3	3	1	3	3	0	3	32
6	Lazzerni et al (2023)	2	2	3	3	NA	3	2	3	NA	3	3	1	2	27
6	Dorman et al (2021)	3	3	3	3	2	3	3	3	2	3	3	3	3	37
6	Scarinci et al (2022)	3	3	3	2	2	3	3	2	2	2	2	0	3	30

### 5.2 Relevant Mobile Apps

**Table:** Second Edition Evidence

Rec.	Source (reference)	QUADS criteria													Total
		1	2	3	4	5	6	7	8	9	10	11	12	13	
1	Kang et al (2025)	1	2	1	2	0	0	1	0	0	1	1	1	2	12
1	Romero et al (2019)	2	3	2	3	3	3	2	3	2	3	3	2	2	33
3	Zitelli & Mormer (2020)	1	1	1	1	1	1	1	1	1	1	1	1	1	13

### 5.3 Alerting Devices and Other Assistive Listening Solutions

**Table:** First Edition Evidence

Rec.	Evidence	Source (reference)	Level	Grade	EF/EV	MM/P/CI
1	The FOCAS is a single, clinical tool that: (i) Integrates family-centered care (FCC) so that clients and their families develop shared goals, are fully informed of the various rehabilitation options available and are central in deciding which is best for them (ii) Explores the emotive impact of hearing loss (iii) Considers holistic hearing needs, including both near- and far-field situations	Crowhen & Turnbull 2018	4	C	EV	MM
1	Some deaf seniors are early and enthusiastic adopters of technology, some are more hesitant, and some are in between. Technology deployment strategies will need to be tailored for everyone.	Singleton (2018)	4	B	EV	–
2	For assistive technologies: There is a serious lack of expertise and skills available in most countries	de Witte et al (2018)	6	B	EF	
2	A study of 208 NHS sites was surveyed, including ENT and Audiology departments. A significant lack of “deaf awareness” among frontline staff was identified	Jama et al (2019)	4	B	EV	–
2	Poor training is also a problem in low- and medium-income countries.	McPherson (2014)	6	D	EV	–
2	There is a disproportionately high level of morbidity among deaf and hard of hearing during natural disasters	Romero et al (2018)	4	C	EV	–
2	Voice-only cell/mobile phone is very difficult	Singleton (2018)	4	B	EV	–
2	Text-based communication is highly used, with the majority using smartphones, social media, email, SMS texting	Singleton (2018)	4	B	EV	–
2	Ensure the safety and appropriateness of Apps	Romero et al (2019)	4	C	EF	–
2	Majority no longer use TTY (Text-Telephone also called TDDs)	Singleton (2018)	4	B	EV	–
2	Many use closed-caption television	Singleton (2018)	4	B	EV	–
2	Many use alert technology: flashing-light alerts, e.g., doorbell, phone, etc, vibrating alarm	Singleton (2018)	4	B	EV	–
2	Minority use Home-security systems	Singleton (2018)	4	B	EV	–
2	Many use internet-based video conferencing for sign-language conversation	Singleton (2018)	4	B	EV	–
2	Automated sign-language translation is under development but currently cannot be applied in real-time	Hermawati & Pieri (2019)	1	A	EV	–
2	Sign-language interpreters continue to be needed	Singleton (2018)	4	B	EV	–
2	For a review and recommendation of a range of assistive technologies for severe and profound hearing loss: See Hermawati et al (2019) Table 3 p. 8	Hermawati & Pieri (2019)	1	A	EV	–
3	There has been a focus on high-end technical solutions in recent research and developments. There is a great need for low-tech and affordable assistive technologies. There are… advantages of scale when accessibility of the environment is addressed in the community, workplace and public settings, e.g., through hearing loops	MacLachlan (2018; WHO)	6	D	EF	–
3	Of 208 reception points surveyed, 64% of Audiology clinics and 42% of ENT clinics had communication assistive devices available for clients. Eighty-three percent of the devices were telecoils	Jama et al (2019)	4	B	EV	
3	In response to one looped venue of their choice, 458 participants rated 756 venues on a 10-point scale where 1 = “heard nothing” to 10 = ”heard every word.” Ratings of ≥8 were given by13.5% of participants for hearing aid alone and 86% for the hearing loop system	Kochkin et al (2014)	4	B	EV	–
3	90% reported that the loop system increased their satisfaction with their hearing aids and cochlear implants	Kochkin et al (2014)	4	B	EV	–
4	Working demos in the clinic client rooms increase client involvement and unprompted engagement with working demos	Bankaitis (2007)	6	D	EV	–
4	Demos were reported to be beneficial for “learning the basics,” but limited in experimenting with a product thoroughly. Some participants preferred trial versions with relatively extended periods	Ding et al (2015)	4	C	EV	–
4	70% of hearing-impaired clients reported that they were not informed about hearing assistive technology (except hearing aids)	Bankaitis (2007)	6	D	EV	–
5	Expert opinion		6	D	–	–
6	Humanitarian activities can include commitments to provide affordable hearing assistive devices, including professional associations, charitable foundations, and faith-based organizations	McPherson (2014)	6	D	EV	–
6	Systems to provide Assistive technology have been in place for many years as part of national and healthcare welfare systems. This will increase following the United Nations Convention on the Rights of Persons with Disabilities (2006)	de Witte et al (2018)	6	B	EF	–
7	Dog recipients reported significant reductions in hearing-related problems with environmental sounds, reduced tension and anxiety, depression, and increased social involvement and independence	Guest et al (2006)	1	A	EV	–
		Rintala et al (2008)	1	A	EV	–
7	Dogs helped with safety by alerting to someone calling the dog recipient's name, finding help in an emergency, door and elevator bells	Rintala et al (2008)	1	A	EV	–
7	Hearing dog owners reported decreased loneliness, increased socializing in the hearing community, and scored lower on life stress scores that control	Hart et al (1996)	1	A	EV	–

**Table:** Second Edition Evidence

Rec.	Source (reference)	QUADS criteria													
		1	2	3	4	5	6	7	8	9	10	11	12	13	Total
1, 2, 3, 7	Atcherson (2022) Discussion paper	1	2	0	1	0	0	0	0	0	0	1	2	0	7

### 5.4 Auracast

**Table:** Second Edition Evidence

Rec.	Source (reference)	QUADS criteria													
		1	2	3	4	5	6	7	8	9	10	11	12	13	Total
1	Baghel et al (2023)	0	0	0	0	0	0	0	0	0	0	0	0	0	0
1	Chandula & Thukivakam (2025)	0	0	0	0	0	0	0	0	0	0	0	0	0	0

### 5.5 Cochlear Implant Candidacy and Bimodal Hearing

**Table:** First Edition Evidence

Rec.	Evidence	Source (reference)	Level	Grade	EF/EV	MM/P/CI
1	Of adults presenting for Cochlear Implant assessment, only 30% of candidates were found to have sufficient hearing aid gain to achieve the NAL_NL2 target audibility for 60 dB SPL speech	Holder et al (2018)	3	C	EV	P, CI
1	177 of 287 patients presented for CI assessment with no hearing aid, reporting lack of perceived benefit. (Holder, Reynolds, Sunderhaus & Gifford, 2018, p. 4)	Holder et al (2018)	3	C	EV	P, CI
1	A period of amplification may be mandatory prior to implantation. See BAA (2020) Cochlear Implants. BCIG (202) NICE Cochlear implants (2019) CMS (2005)	BAA (2020)				CI
		CMS (2005)				
		NICE (2019)				CI
		BCIG (2020)				CI
1	Wireless microphone technology can be considered as a standard component of a rehabilitation program	Thibodeau (2019a)	3	B	EF	MM, CI
2	CI candidacy criteria change over time: see CMS (2005) AND NICE Cochlear implants (2019)	CMS (2005)				CI
		NICE (2019)				
		Raine et al (2016)	6	D	–	CI
		BCIG (2017)				CI
3	Average preoperative word recognition score with appropriately fitted power hearing aids was just 8.7% correct	Holder et al (2018)	3	C	EV	P, CI
3	Providers referring for CI waited until this group was missing over 90% of the auditory speech signal, on average	Holder et al (2018)	3	C	EV	P, CI
3	Cochlear implantation is considered the standard of care treatment for adults with severe and profound sensorineural hearing loss	Wilson (2018)	4	c	EF	CI
3	Cochlear implantation is not only appropriate when a patient receives insufficient benefit from their hearing aids when listening in quiet, but can also be appropriate when hearing aids provide insufficient benefit only when listening in background noise. Cochlear implantation can also be appropriate in patient groups where speech understanding is not possible or appropriate to measure	BCIG (2017)				CI
4	Start the conversation by introducing CI as a part of a continuum of care starting with hearing aid use and ultimately progressing to CI candidacy	Helms et al (1997)	3	C	EF EV	CI
		Koch et al (2004)	3	C	EF EV	CI
		Balkany et al (2007)	2	B	EF EV	CI
		Blamey et al (1996)	1	A	EF	CI
		Rubinstein et al (1999)	3	B	EF	CI
		Friedland et al (2003)	4	C	EF	CI
4	Hearing Health Professionals should become confident in discussing the benefits and outcomes of Cochlear Implantation so they can adequately address client questions and concerns. This may require attending regular training and continued professional development sessions. For evidence, see BAA (2020) Cochlear Implants	BAA (2020)				CI
5	A recent study of 287 adult patients at a large academic medical center revealed that over 95% of adults referred for preoperative CI evaluation met labeled candidacy criteria	Holder et al (2018)	3	C	EV	P, CI
	Evidence shows large, life-changing benefits postimplantation, the magnitude of which cannot begin to be achieved through the use of hearing aid technology alone. Examples are average sentence recognition scores in quiet jumped from 10% preimplantation to 77% postimplantation in 110 adult patients implanted in 2017–2018. For evidence see BAA (2020) Cochlear Implants	BAA (2020)				CI
5	Adults with bilateral severe and profound sensorineural hearing loss derive significant communication benefit from cochlear implantation	Helms et al (1997)	3	C	EF EV	CI
		Koch et al (2004)	3	C	EF EV	CI
		Balkany et al (2007)	2	B	EF EV	CI
5	Only 5 to 7% of adults in the United States with qualifying hearing loss actually receive a CI	Sorkin & Buchman (2016)	6	C	–	CI
		Sorkin (2013)	4	C	EV	CI
5	<7% of estimated eligible adults receive a CI	BAA (2020)				CI
5	The range of patient groups in which cochlear implantation is appropriate is considerably broader than the range of groups who are currently eligible according to NICE guidance	BCIG (2017)				CI
6	For evidence see BAA (2020) Cochlear Implants	BAA (2020)				CI
6	Speech tests are too unreliable to use to establish a specific criterion or cut-off for candidacy, but their results should be considered by the multidisciplinary team	BCIG (2017)				CI
6	Assessment for CI candidacy will be by a multidisciplinary team.	BCIG (2020)				CI
7	For evidence see BAA and NICE Cochlear implants (2019)	BAA (2020) NICE (2019)				CI
8	For evidence see BAA (2020) Cochlear Implants	BAA (2020)				CI
8	On completion of the assessment pathway a comprehensive report should be provided to the referrer, the patient's GP and the patient as indicated	BCIG (2020)				CI
9	Audit to ensure that all staff are delivering treatment that is safe, accurate and effective	BCIG (2020)				CI
9	For evidence see BAA (2020) Cochlear Implants	BAA (2020)				CI
10	For evidence see BAA (2020) Cochlear Implants	BAA (2020)				CI

**Table:** Second Edition Evidence

Rec.	Source (reference)	QUADS criteria													
		1	2	3	4	5	6	7	8	9	10	11	12	13	Total
1	Neukam et al, 2024	3	3	3	3	3	3	3	3	3	3	3	3	3	39
1	Carlson M (2020)	2	2	1	2	1	2	2	2	2	2	2	2	1	21
1	CIICA-Consensus-Statement.pdf or Living Guidelines | Adult Hearing	3	3	3	3	3	3	3	3	3	3	3	3	3	39
1	Cullington (2023)	3	3	3	3	2	3	2	2	1	3	3	3	3	32
1	Hoppe et al (2020)	3	3	3	3	3	2	2	3	3	3	3	3	3	37
1	Zeitler et al (2023)	3	3	3	3	3	3	3	3	3	3	3	3	3	39
2	Zwolan (2021)	3	3	3	3	3	3	3	3	3	3	3	3	2	38
3	BAA (2025)	3	3	3	3	3	3	3	3	3	3	3	3	3	39

#### 5.6.4 Understand the Benefits of Bimodal Fitting

**Table:** First Edition Evidence

Rec.	Evidence	Source (reference)	Level	Grade	EF/EV	MM/P/CI
1	Of adults presenting for Cochlear Implant assessment, only 30% of candidates were found to have sufficient hearing aid gain to achieve the NAL_NL2 target audibility for 60 dB SPL speech	Holder et al (2018)	3	C	EV	P, CI
1	177 of 287 patients presented for CI assessment with no hearing aid, reporting lack of perceived benefit. (Holder, Reynolds, Sunderhaus & Gifford, 2018, p. 4)	Holder et al (2018)	3	C	EV	P, CI
1	A period of amplification may be mandatory prior to implantation. See BAA (2020) Cochlear Implants. BCIG (202) NICE Cochlear implants (2019) CMS (2005)	BAA (2020)				CI
		CMS (2005)				
		NICE (2019)				CI
		BCIG (2020)				CI
1	Wireless microphone technology can be considered as a standard component of a rehabilitation program	Thibodeau (2019a)	3	B	EF	MM, CI
2	CI candidacy criteria change over time: see CMS (2005) AND NICE Cochlear implants (2019)	CMS (2005)				CI
		NICE (2019)				
		Raine et al (2016)	6	D	–	CI
		BCIG (2017)				CI
3	Average preoperative word recognition score with appropriately fitted power hearing aids was just 8.7% correct	Holder et al (2018)	3	C	EV	P, CI
3	Providers referring for CI waited until this group was missing over 90% of the auditory speech signal, on average	Holder et al (2018)	3	C	EV	P, CI
3	Cochlear implantation is considered the standard of care treatment for adults with severe and profound sensorineural hearing loss	Wilson (2018)	4	c	EF	CI
3	Cochlear implantation is not only appropriate when a patient receives insufficient benefit from their hearing aids when listening in quiet, but can also be appropriate when hearing aids provide insufficient benefit only when listening in background noise. Cochlear implantation can also be appropriate in patient groups where speech understanding is not possible or appropriate to measure	BCIG (2017)				CI
4	Start the conversation by introducing CI as a part of a continuum of care starting with hearing aid use and ultimately progressing to CI candidacy	Helms et al (1997)	3	C	EF EV	CI
		Koch et al (2004)	3	C	EF EV	CI
		Balkany et al (2007)	2	B	EF EV	CI
		Blamey et al (1996)	1	A	EF	CI
		Rubinstein et al (1999)	3	B	EF	CI
		Friedland et al (2003)	4	C	EF	CI
4	Hearing Health Professionals should become confident in discussing the benefits and outcomes of Cochlear Implantation so they can adequately address client questions and concerns. This may require attending regular training and continued professional development sessions. For evidence, see BAA (2020) Cochlear Implants	BAA (2020)				CI
5	A recent study of 287 adult patients at a large academic medical center revealed that over 95% of adults referred for preoperative CI evaluation met labeled candidacy criteria	Holder et al (2018)	3	C	EV	P, CI
	Evidence shows large, life-changing benefits postimplantation, the magnitude of which cannot begin to be achieved through the use of hearing aid technology alone. Examples are average sentence recognition scores in quiet jumped from 10% preimplantation to 77% postimplantation in 110 adult patients implanted in 2017–2018. For evidence see BAA (2020) Cochlear Implants	BAA (2020)				CI
5	Adults with bilateral severe and profound sensorineural hearing loss derive significant communication benefit from cochlear implantation	Helms et al (1997)	3	C	EF EV	CI
		Koch et al (2004)	3	C	EF EV	CI
		Balkany et al ( et al (2007)	2	B	EF EV	CI
5	Only 5 to 7% of adults in the United States with qualifying hearing loss actually receive a CI	Sorkin & Buchman (2016)	6	C	–	CI
		Sorkin (2013)	4	C	EV	CI
5	<7% of estimated eligible adults receive a CI	BAA (2020)				CI
5	The range of patient groups in which cochlear implantation is appropriate is considerably broader than the range of groups who are currently eligible according to NICE guidance	BCIG (2017)				CI
6	For evidence see BAA (2020) Cochlear Implants	BAA (2020)				CI
6	Speech tests are too unreliable to use to establish a specific criterion or cut-off for candidacy, but their results should be considered by the multidisciplinary team	BCIG (2017)				CI
6	Assessment for CI candidacy will be by a multidisciplinary team	BCIG (2020)				CI
7	For evidence see BAA and NICE Cochlear implants (2019)	BAA (2020) NICE (2019)				CI
8	For evidence see BAA (2020) Cochlear Implants	BAA (2020)				CI
8	On completion of the assessment pathway a comprehensive report should be provided to the referrer, the patient's GP and the patient as indicated	BCIG (2020)				CI
9	Audit to ensure that all staff are delivering treatment that is safe, accurate and effective	BCIG (2020)				CI
9	For evidence see BAA (2020) Cochlear Implants	BAA (2020)				CI
10	For evidence see BAA (2020) Cochlear Implants	BAA (2020)				CI

**Table:** Second Edition Evidence

Rec.	Source (reference)	QUADS criteria													
		1	2	3	4	5	6	7	8	9	10	11	12	13	Total
1	Casarella (2024)	3	3	3	3	3	3	3	3	3	3	3	3	3	39
1	Kelsall (2021)	3	3	3	3	3	3	3	3	3	3	3	2	2	37
1	Firszt et al (2023)	3	3	3	3	3	3	3	3	3	3	3	2	3	38
2	Mertens (2024)	2	3	2	2	2	2	2	3	2	2	2	2	2	27
2	Vroegop (2018)	3	3	3	3	3	3	3	3	3	3	3	3	2	38
2	Holder et al (2022)	3	3	3	3	3	3	3	3	3	3	3	3	2	38

#### 5.6.5 Other Implantable Devices: Understand the Limitations for this Population

**Table:** First Edition Evidence

Rec.	Evidence	Source (reference)	Level	Grade	EF/EV	MM/P/CI
1	Middle ear implants require a functional and intact middle ear system, and both middle ear implants and bone-anchored implants require sufficiently functioning inner hair cells for effective cochlear stimulation, as 95% of afferent auditory nerve fibers are innervated by our inner hair cells	FDA, 2023				
1	Middle ear implants are currently approved for use with adults in the United States who have sensorineural hearing losses ranging from mild to severe and profound	FDA, 2023				
1	Middle ear implants offer an effective method of rehabilitating moderate-to-severe SNHL	Kahue 2014	4	B	EV	
1	Bone-anchored implants are approved for use with adults with bilateral mixed hearing losses for which the pure tone average, obtained via bone conduction, is £ 65 dB HL	FDA, 2023				
1	Individuals with audiometric thresholds > 60 dB HL have a significantly greater incidence of cochlear dead regions—or areas of complete inner hair cell dysfunction	Vinay & Moore (2007)	3	B	EF	–
		Hornsby & Dundas (2009)	4	B	EF	–
		Pepler et al (2014)	4	B	EF	–
		Chang et al (2019)	4	B	EF	–

**Table:** Second Edition Evidence

Rec.	Source (reference)	QUADS criteria													
		1	2	3	4	5	6	7	8	9	10	11	12	13	Total
1	Rodrigues da Silva (2025)	3	3	3	3	3	3	3	3	3	3	3	3	3	39
1	Maier (2022)	3	3	3	3	3	3	3	3	3	3	3	3	3	39
1	Ontario HTA (2020)	3	3	3	3	3	3	3	3	3	3	3	3	3	39
1	Wang (2024)	3	3	3	3	3	3	3	3	3	3	3	3	3	39
2	NICE (2005)	0	0	0	0	0	0	0	0	0	0	0	0	0	0

### 5.7 Remote Fittings

#### 5.7.1 Remote Initial Fittings

**Table:** Second Edition Evidence

Rec.	Source (reference)	QUADS criteria													
		1	2	3	4	5	6	7	8	9	10	11	12	13	Total
1	Venail et al (2019)	3	3	3	3	3	3	3	3	3	3	3	2	3	38
1	Dillard et al (2024)	3	3	3	3	3	3	3	3	3	3	3	2	3	38
1	Frisby et al (2022)	2	3	3	2	2	2	3	3	2	2	3	2	3	32
1	Swanepoel (in press)	2	3	3	2	NA	2	3	2	NA	2	3	2	3	27
1	ASHA (2021)	2	3	NA	NA	NA	2	2	2	NA	3	2	3	3	22
1	Audiology Australia (2022)	2	3	NA	NA	NA	2	2	2	NA	3	2	3	3	22
1	BAA/BSA (2021)	2	3	NA	NA	NA	2	2	2	NA	3	2	3	3	22
2	Benson et al (2018)	2	3	3	2	2	2	3	2	1	2	3	2	3	30
3	Bagatto & Scollie (2010)	2	3	3	3	2	3	3	3	2	3	3	2	3	35
3	Dillon (2012)	1	3	2	2	NA	NA	3	2	NA	2	3	2	2	22

#### 5.7.2 Remote Fine-Tuning and Follow-Up

**Table:** Second Edition Evidence

Rec.	Source (reference)	QUADS criteria													
		1	2	3	4	5	6	7	8	9	10	11	12	13	Total
1	Tao et al (2018)	3	3	3	3	3	3	3	3	3	3	3	2	3	38
1, 4, 5	Tao et al (2021)	3	3	3	3	3	3	3	3	3	3	3	2	3	38
1	Venail et al (2019)	3	3	3	3	3	3	3	3	3	3	3	2	3	38
1, 4	Malmberg & Hagberg (2024)	3	3	3	3	3	3	3	3	3	3	3	2	3	38
1, 4	Malmberg & Hagberg (2025)	3	3	3	3	3	3	3	3	3	3	3	2	3	38
2	Cox & Alexander (2000)	3	3	3	3	3	3	3	3	3	3	3	2	3	38
2, 7	Kokkonen et al (2023)	2	3	3	2	2	2	3	3	2	2	3	2	3	32
3	Benson et al (2018)	2	3	3	2	2	2	3	2	1	2	3	2	3	30

#### 5.7.3 Patient and Clinician Perspectives

**Table:** Second Edition Evidence

Rec.	Source (reference)	QUADS criteria													
		1	2	3	4	5	6	7	8	9	10	11	12	13	Total
5.7.2 Rec 6	Blyth & Saunders (2024)	3	3	3	3	3	2	3	3	2	3	3	3	3	37

#### 5.7.4 Infrastructure and Future Directions

**Table:** Second Edition Evidence

Rec.	Source (reference)	QUADS criteria													
		1	2	3	4	5	6	7	8	9	10	11	12	13	Total
5.7.2 Recs 5, 6	DiFabio et al (2022)	3	3	3	3	3	2	3	3	3	3	3	2	3	37
5.7.2 Rec 6	Ferguson (2023)	2	3	3	2	2	2	3	2	1	2	3	2	3	30

#### 5.7.5 Safety and Risk Management

**Table:** Second Edition Evidence

Rec.	Source (reference)	QUADS criteria													
		1	2	3	4	5	6	7	8	9	10	11	12	13	Total
5.7.1 Recs 1, 2	BAA/BSA (2021)	2	3	NA	NA	NA	2	2	2	NA	3	2	3	3	22
5.7.1 Recs 1, 2	Audiology Australia (2022)	2	3	NA	NA	NA	2	2	2	NA	3	2	3	3	22
5.7.1 Recs 1, 2	DiFabio et al (2022)	3	3	3	3	3	2	3	3	3	3	3	2	3	37

#### 5.7.6 Special Considerations for Severe-to-Profound Hearing Loss

**Table:** Second Edition Evidence

Rec.	Source (reference)	QUADS criteria													
		1	2	3	4	5	6	7	8	9	10	11	12	13	Total
5.7.1 Recs 1–3	Glista et al (2009)	2	3	3	3	3	2	3	3	2	3	3	2	3	35
5.7.1 Recs 1–3	Alexander (2013) ^a^	2	3	3	3	2	2	3	3	2	3	3	2	3	34
5.7.1 Recs 1–3	Benson et al (2018)	2	3	3	2	2	2	3	2	1	2	3	2	3	30
5.7.2 Rec 7	Kokkonen et al (2023)	2	3	3	2	2	2	3	3	2	2	3	2	3	32

## Rehabilitation and Other Treatment Considerations

### 5.1 Rehabilitation: Communication and Psychosocial Concerns

**First Edition Evidence**

### 3.1 Help in Adjusting to Life with Severe and Profound Hearing Loss

**Table:** 

Rec.	Evidence	Source (reference)	Level	Grade	EF/EV	MM/P/CI
1	Communication support is a key reasonable adjustment. Hearing care professionals should take steps to be as accessible as possible, e.g., by: offering a range of contact methods, recording and meeting communication needs, providing deaf awareness training for all staff, installing and maintaining loop or infrared systems, providing communication support such as digital text-based apps, speech-to-text reporters and sign language interpreters when appropriate, and captioning video content	Action on HL (2015)	6	D	–	–
2	Clients can be confused when their clinical management is inappropriately driven by algorithmic protocols and ignores their individual circumstances	Greenhalgh et al (2014)	6	D	–	–
2	Some people describe difﬁculty in communicating their problems to their Hearing Care Professional, and the use of tools to facilitate this process may help	Manchaiah & Stephens (2011)	4	C	EV	MM
2	Instructional materials for all literacy levels are an important part of improving self-management skills	Arnold et al.(2019)	4	C	–	–
3	It is important to evaluate clients' communication and relationship challenges across all aspects of their life	Bess (2000)	4	C	–	–
3	Individual differences in how older people adjust to hearing loss are large	Manchaiah & Stephens (2011)	4	C	EV	MM
3	Management in audiology services should include the person's hearing and communication needs at home, at work or in education, and in social situations; any psychosocial difficulties related to hearing; the person's expectations and motivations with respect to their hearing loss and the listening and communication strategies available to them	Valente et al (2005)	3	B	–	–
3	Establish client-specific communication needs and realistic expectations from treatment, including client-specific goals	Valente et al (2005)	3	B	–	–
4	The audiologist can improve the client's activity, participation and quality of life by ensuring both external (lifestyle, social attitudes) and internal (age, education, coping style, personal expectations) factors are addressed	Boothroyd (2007)	6	D	–	–
4	Assessment and management in audiology services should include the person's hearing and communication needs at home, at work or in education, and in social situations; any psychosocial difficulties related to hearing; the person's expectations and motivations with respect to their hearing loss and the listening and communication strategies available to them	Valente et al (2005)	3	B	–	–
5	Third-party disability can be experienced by a family member, and so they should be included in the assessment and rehabilitation for their family member	Meyer et al (2015)	3	B	EV	MM
		Preminger & Meeks (2012)	3	B	EV	MM
		Scarinci et al (2012)	3	B	EV	MM
5	Hearing loss affects both the client and their communication partner. Aligned coping strategies can facilitate adjustment to hearing loss	Bentler & Kramer (2000)	6	D	EV	MM
		Ekberg et al.(2015)	4	B	EF,EV	MM
		Meyer et al (2015)	3	B	EV	MM
5	While family members currently have minimal participation in audiology appointments, they display a strong interest in being involved and sharing their experience—best practice will demonstrate family-centered care principles in audiology practice	Ekberg et al.(2015)	4	B	EF,EV	MM
6	Motivation and behavior after fitting of hearing aids needs more attention than motivation to use them in the first place	Sawyer et al (2019)	3	B	EV	
6	Information and counseling are important to ensure effective self-management	Borg & Borg (2015)	4	B	EV	–
6	Instruction, cognitive training, and motivational engagement grounded in behavior change theory are important in optimizing outcomes	Ferguson et al (2019)	1	A	–	–
6	Motivational engagement can be beneficial in early client management	Ferguson et al (2016b)	3	B	EV	
6	Motivational engagement enhances client participation in shared decision-making and improves their understanding of the issues	Ferguson et al (2016b)	3	B	EV	
7	An in-depth inquiry on the client's listening satisfaction, to clarify and expand on questionnaire responses, may help in furthering our understanding of people with severe and profound hearing loss. The differences in client and professional perspectives may be attributed to differences in educational, ethnic, and socioeconomic backgrounds. These differences in perspectives can have important implications for the effective management of illness	Gottermeier & De Filipp (2018)	3	C	EV	–
7	When changing the negative reactions of a person with severe and profound hearing loss to ampliﬁcation, frequent communication and personal contact with the Hearing Care Professional and discussion of what they should expect from newer technology is important	Gottermeier & De Filipp (2018)	3	C	EV	–
8	Effective management of sudden hearing losses requires a comprehensive and multi-disciplinary perspective	Carlsson et al (2011)	4	B	EV	–
8	People with sudden hearing loss are more than twice as likely as those with normal hearing to develop depressive disorders	Tseng et al (2016)	4	C		
8	Sudden hearing loss is associated with higher than normal levels of anxiety disorder	Chung et al (2015)	4	C	–	–
8	Clients with severe and profound hearing loss require early assessment and intervention for depression and anxiety	Carlsson et al (2014)	4	B	EV	CI
8	Both depression and anxiety are higher in people with hearing loss	Kvam et al (2007)	4	C	EF	–
8	Mental ill-health is associated with severe hearing loss	De Graaf & Bijl, (2002)	4	C	EF	–
9	Incorporating information about a client's self-management of their hearing loss improves clinical decision making and management planning	Convery et al (2019)	3	C	EV	–

### 3.2 Psychosocial and Communication Rehabilitation: Training to Develop Effective Communication Strategies, in Client and Family

**Table:** 

Rec.	Evidence	Source (reference)	Level	Grade	EF/EV	MM/P/CI
1	The client's health problems are always contextualized in the everyday life activities of the client	Tjørnhøj-Thomsen (2009)	6	D	EV	–
2	The clinician should aim to understand the client's experience of hearing loss to find out the individual needs that would lead to an individualized rehabilitation plan	BSA Rehabilitation (2016)	1	A	EV	–
3	The psychosocial concerns of the client should be addressed in the appointments to increase their motivation	Ekberg et al (2014)	4	B	EV	–
3	Motivational engagement early in the client journey might have some positive effects on the rehabilitation process	Ferguson et al (2016b)	3	B	EV	–
3	The empirical findings of the study indicate that motivation is an important contributor to decision-making in hearing rehabilitation	Ridgway et al (2015)	4	B	EV	MM
3	It is important to help clients using volitional processes to translate high motivation into behavior	Sawyer et al (2019)	3	B	EV	–
4	Auditory training or perceptual learning tends to induce plastic changes in the brain. Therefore, it seems plausible that motivation of the client to consistently and intensively train for a longer time is necessary for success. client	Stropahl et al (2019)	1	A	EF	–
5	Cued speech is able to enhance speech perception in patients with severe and profound hearing impairment	Bayard et al (2019).	3	B	EF	P
5	If clients acquire severely maladaptive communication strategies, onward referral to an external source of communication support is often helpful to support the client and the audiologist	Hallam et al (2008)	4	B	EV	
6	There is a variety of options on auditory rehabilitation that should be known by the audiologist and recommended to the client based on individual needs	BSA Rehabilitation (2016)	1	A	EV	–
7	Computerized auditory training supports auditory rehabilitation	Henshaw & Ferguson (2013)	1	A	EV	–
7	Evidence exists that the internet/online tools are valid for interventions of auditory rehabilitation for hearing-aid users	Thorén et al (2014)	2	A	EF	–
8	Client-centered approaches support people to develop effective ways to manage their condition independently	BSA Rehabilitation (2016)	1	A	EV	–
8	Clients with severe and profound hearing loss need to manage their condition on their own most of the time. Audiologists therefore should facilitate the self-management of their clients	Barker et al (2015)	4	B	EV	–
9	A rehabilitation program that includes the significant other has a positive effect on the attitude of the client with hearing impairment and the significant other toward the hearing aids	Kramer et al (2005)	1	A	EV	–
		Barker et al (2017)	1	A	EV	MM
10	It is proposed that the use of scientifically developed change behavior models enhances audiological rehabilitation	Coulson et al (2016)	6	D	EF	–
10	The use of hearing health behavior change theories is increasing to support help-seeking clients with hearing impairment	Ferguson et al (2016a)	6	D	EF	–
10	There is reasonably good evidence that participation in an adult aural rehabilitation program provides short-term reduction in self-perception of hearing handicap and potentially better use of communication strategies and hearing aids	Hawkins (2005)	1	A	EV	

### 3.3 Psychosocial and Communication Rehabilitation: Contact with Peers to Provide Support and Reduce Isolation

**Table:** 

Rec.	Evidence	Source (reference)	Level	Grade	EF/EV	MM/P/CI
4	The intensive rehabilitation program included full integration of family members into all aspects of the programme, peer education whereby specially trained deafened people provide most of the education and guidance, and an emphasis on learning and therapy through group work	Sherbourne et al (2002)	4	B	EV	–
1	Around 65 to 70% of participants were in regular contact with other people with hearing impairments. In around 40%, this was through a self-help organization	Hallam (2006)	4	C	EV	–
1	Significant effects from attending the programme included training provided by deafened people who have personal experience of acquired deafness, and who offer realistic managing skills and positive role models	Sherbourne et al (2002)	4	B	EV	–
2	Access to Peer Support Group services is often mediated by Hearing Health Care Professionals	Southall et al (2019)	4	C	EV	–
2	Peer Support Group referral is low, ranging from less than 5% of hearing aid users (Kochkin et al, 2010) to 19.1% of audiological clients with hearing loss (Stika & Ross, 2006)	Southall et al (2019)	4	C	EV	–
3	As might be expected, audiologists were most consistently regarded as useful, followed by medical consultants. Help received when first deafened was usually regarded as more useful than help currently received	Hallam (2006)	4	C	EV	–
3	A qualitative study of 10 adults with profound hearing loss reported that the benefits of Peer Support Group involvement were: (1) practical and accessible information about hearing loss; (2) social belonging leading to personal transformation; and (3) a new and mutually beneficial direction	Southall et al (2019)	4	C	EV	–
4	The intensive rehabilitation program included full integration of family members into all aspects of the program, peer education whereby specially trained deafened people provide most of the education and guidance, and an emphasis on learning and therapy through group work	Sherbourne et al (2002)	4	B	EV	–
4	Significant effects of the program included participation of carers in all aspects of a program that is designed to address their needs too	Sherbourne et al (2002)	4	B	EV	–
5	Enhanced collaboration between Hearing Health Care Professionals and Peer Support Group organizers may lead to a more comprehensive level of hearing health care than what is currently offered by Aural Rehabilitation programs or Peer Support Groups alone	Southall et al (2019)	4	C	EV	–
6	A support center for people with hearing loss found that 51.1% reported that as a result they took part in activities more. The services' benefit was that early intervention by rehab services can help maintain safety, help maintain independence, and minimize the emotional impact of hearing loss	Smith (2016)	4	B	EV	MM

#### 5.1.1 Addressing the Individual Psychosocial Impact of Severe and Profound Hearing Loss

**Table:** Second Edition Evidence

Rec.	Source (reference)	QUADS criteria													
		1	2	3	4	5	6	7	8	9	10	11	12	13	Total
3, 4, 8	Timmer et al, 2024	3	2	1	3	0	2	0	2	0	2	0	1	0	16
3	Ekberg et al, 2025	3	2	1	0	0	2	1	2	1	0	1	2	2	17
4, 6, 7	Bennett et al, 2021	3	3	3	2	2	2	3	2	2	3	3	3	2	33
4, 9, 10	Bennett et al, 2023	3	3	2	3	1	3	3	3	1	2	3	2	2	31

#### 5.1.2 Incorporating Family in Rehabilitation for Shared Psychosocial Well-Being

**Table:** Second Edition Evidence

Rec.	Source (reference)	QUADS criteria													
		1	2	3	4	5	6	7	8	9	10	11	12	13	Total
2	Ekberg et al, 2023	2	3	3	2	2	2	3	3	2	2	3	2	3	32
1, 4, 5	Timmer et al, 2024	3	2	1	3	0	2	0	2	0	2	0	1	0	16
3	Meyer et al, 2025	2	3	3	2	2	2	3	3	2	2	3	2	3	32

#### 5.1.3 Building Psychosocial Support in the Community and Workplace

**Table:** Second Edition Evidence

Rec.	Source (reference)	QUADS criteria													
		1	2	3	4	5	6	7	8	9	10	11	12	13	Total
2, 3	Timmer et al, 2024	3	2	1	3	0	2	0	2	0	2	0	1	0	16
7	Hogan et al, 2021	2	0	1	1	1	0	1	1	1	0	1	1	2	12
4, 10	Bennett et al, 2023	3	3	2	3	1	3	3	3	1	2	3	2	2	31

#### 5.1.4 Addressing Psychosocial Impact for Deaf Sign Language Users

Second edition evidence—see Table 2.1

### 5.2 Tinnitus

**Table:** First Edition Evidence

Rec.	Evidence	Source (reference)	Level	Grade	EF/EV	MM/P/CI
1	Hearing care professionals should use at least one measure of tinnitus-related disability if tinnitus is reported that impairs emotion, cognition, attention, tasks, and daily life	Cima et al (2019)	1	A	EV	MM
	The tinnitus assessment includes an audiologic examination and appropriate questionnaires, such as the Tinnitus Handicap Inventory (THI)	Tunkel et al (2014)	1	A	EV	MM
2	The constant wearing of hearing aids with occluding earmolds increases the risk of impacted earwax or cerumen. Common treatable otologic conditions that cause tinnitus, such as cerumen impaction or other ear canal obstructions, should be excluded first	Tunkel et al (2014)	1	A	EV	MM
3	In most of the cases, the origins of tinnitus are unknown. However, tinnitus may occur due to a specific cause which might be treatable (i.e., cerumen or cardiovascular issues). For a list of known conditions associated with tinnitus, please see Table 7 on page S20 of Cima et al (2019)	Cima et al.(2019)	1	A	EV	MM
4	In the case of severe and profound hearing loss with tinnitus, there are several ontological diseases which are known risk factors for tinnitus, including otosclerosis, Ménière's disease, and vestibular schwannoma (acoustic neuroma)	Baguley et al (2013)	1	A	EV	CI
4.	Long-standing tinnitus should be investigated if the tinnitus changes and the hearing loss is stable	Tunkel et al (2014)	1	A	EV	MM
**Rec.**	**Evidence**	**Source (reference)**	**Level**	**Grade**	**EF/EV**	**MM/P/CI**
1	In the case of hearing loss and tinnitus, hearing aids are recommended to treat hearing loss in the first instance. This is likely to enhance the individual's listening and communication abilities, improve quality of life, and have positive effects on the tinnitus symptoms	Cima et al (2019)	1	A	EV	MM
		Tunkel et al (2014)	1	A	EV	MM
		AAA 2001	1	A	EV	
1	Provision of hearing aids for tinnitus will always have the potential consequence of reducing the distress associated with hearing loss and so any clinical improvement that is specific to tinnitus will always be difficult to estimate accurately	Hoare et al (2014)	1	A	EV	MM
2	Cochlear implantation is recommended only for clients meeting the hearing loss criteria for candidacy and not for the treatment of tinnitus	Cima et al (2019)	1	A	EV	MM
2	Cochlear implantation is not only appropriate where the primary motivation for treatment is the restoration of speech understanding but can also be appropriate where it is for the alleviation of tinnitus	BCIG (217)				CI
2	Cochlear implantation improves or eliminates tinnitus in up to 86% of clients with profound hearing loss and tinnitus	Baguley et al (2013)	1	A	EV	CI
2	There is a risk that Cochlear implantation might induce tinnitus in about 9% of the cases	Kompis et al (2012)	3	B	EV	CI
**Rec.**	**Evidence**	**Source (reference)**	**Level**	**Grade**	**EF/EV**	**MM/P/CI**
8	Sound generators (as available in combination devices of hearing aids and sound generators) can provide tinnitus relief by providing sound enrichment	Tunkel et al. (2014)	1	A	EV	MM
		Cima et al. (2019)	1	A	EV	MM
8	However, multiple studies, including two randomized controlled trials (RCTs), found no difference in effectiveness between amplification only and in combination devices	Tutaj et al. (2018)	1	A	EV	–
		Sereda et al. (2018)	1	A	EV	–
8	Hearing aids with tinnitus sound generators must be used with extreme care. In the presence of severe and profound hearing loss, adding generated noise in a hearing aid program which is intended for speech must be avoided due to the high risk that it will reduce speech detection/perception or understanding	Expert opinion	6	D	EV	–
9	There is no evidence that sound therapy for tinnitus is any more effective than no-device methods, such as waiting list control, placebo, or education/information counseling. There is also no evidence that one or another sound therapy option is better than hearing aid alone, including sound generator or combination sound generator and hearing aid	Sereda et al. (2018)	1	A	EV	–
9	Sound therapy may be useful in the treatment of acute tinnitus, but evidence for long-term benefits is lacking. There is no risk for safety but also little evidence for effectiveness	Sereda et al. (2018)	1	A	EV	–
		Cima et al. (2019)	1	A	EV	MM
		Tunkel et al (2014)	1	A	EV	MM
10	For adults with severe and profound hearing loss, sound therapy using environmental enrichment sounds is not recommended due to the high levels of sound needed to provide relief of tinnitus. Other important sounds might be masked by the sound enrichment	Expert opinion	6	D	EV	–
11	For Tinnitus Retraining Therapy (TRT), there is evidence for safety but little high-level evidence for the eﬀectiveness. The finding is based on one RCT and two systematic reviews. TRT is currently not recommended	Cima et al. (2019)	1	A	EV	MM
		Hoare et al. (2011)	1	A	EV	MM
11	Cognitive Behavioral Therapy (CBT) is highly recommended for the treatment of tinnitus. There is high-level evidence for the effectiveness and safety of CBT for tinnitus from both systematic reviews and a more recent RCT	Cima et al. (2019)	1	A	EV	MM
		Hoare et al. (2011)	1	A	EV	MM
11	Cognitive behavior therapy was more effective than controls at posttreatment. Effects were maintained at follow-up and were robust	Hesse et al. (2011)	1	A	EF EV	MM
11	Despite psychological treatment modalities having the best evidence base for successful tinnitus management, only a minority of tinnitus patients ever get to meet a psychologist	McFerran et al. (2018)	4	C	–	MM
11	The multimodal treatment program for tinnitus and hyperacusis, including a specific CBT method, proves to be a highly effective means of significantly reducing not only tinnitus and hyperacusis but also accompanying distress	Nolan et al. (2020)	4	B	–	MM
12	Those who administer therapies and counseling may include professionals and volunteers who are not trained in audiology and may therefore require the support of the HCP in effectively delivering therapies in the presence of severe and profound hearing loss	McKenna et al. (2017)	1	A	EV	–
	It is important to be aware that severe and profound hearing loss may impair communication in a way that can prevent full participation in therapy when it is delivered in a group or online. In the case of severe and profound hearing loss, therapy can be optimally delivered in individual and face-to-face sessions	Expert opinion	6	D	EV	–
13	Anxiety and depression are known comorbidities of Tinnitus and should be addressed in case of occurrence	Baguley et al. (2013)	1	A	EV	CI
	Severe and profound hearing loss is a compounding factor known to be associated with increased rates of depression and anxiety (comorbidities of tinnitus). For example, these factors result in greater reluctance to participate in social occasions, increasing the likelihood of social isolation	Souza & Hoover (2018)	4	B	EV	–
	Severe and profound hearing loss also causes difﬁculty with everyday communication that has implications for work, social activities, and overall health. As a consequence of communication difﬁculty, listeners with severe hearing loss report higher levels of anxiety and stress (comorbidities of tinnitus)	Gevonden (2015)	1	A	EV	–
	For hearing aid wearers with severe and profound hearing loss, annoying tinnitus might have strong negative effects on quality of life. Any resulting anxiety, depression (and vertigo) should be referred for treatment as early as possible in the rehabilitative process	Carlsson et al. (2014)	4	B	EV	CI
	Signs of anxiety or depression can be assessed with appropriate questionnaires. In case of symptoms clients must be referred to appropriate medical staff, especially psychologists	Cima et al. (2019)	1	A	EV	MM
		McFarren et al. (2018)	4	B	EV	MM

#### 5.2.2 Tinnitus Management

**Second Edition Evidence**

##### 5.2.2.4 Hearing Assistance

**Table:** 

Recommendations	Source (reference)	QUADS criteria													Total
		1	2	3	4	5	6	7	8	9	10	11	12	13	
1, 2	(Ueno et al., 2025)	2	2	3	3	1	3	3	2	2	2	2	1	2	28
3	3 (Dixon et al., 2020)	2	2	3	2	2	2	3	3	3	2	3	1	3	31
3	(Pierzycki & Kitterick, 2021)	3	3	3	2	1	3	3	3	3	3	3	2	2	34
3	(Yang et al., 2021)	2	2	1	3	1	2	3	2	2	2	3	0	1	24
3	(Assouly et al., 2022)	3	3	2	2	1	3	3	3	3	3	3	1	2	32
3	(González et al., 2022)	2	2	2	1	1	2	2	1	1	1	2	0	1	18
3	(Chau et al., 2023)	2	2	1	2	0	1	2	1	2	1	2	0	2	18
3	(Quaranta et al., 2023)	1	2	2	2	1	1	2	2	2	2	2	0	2	21
3	(Anari et al., 2024)	2	2	2	2	2	2	2	2	3	1	2	0	1	23
3	(Perreau et al., 2021)	3	3	3	2	0	2	3	3	3	2	3	1	3	31

##### 5.2.2.5 Sound-Based Therapies

**Table:** 

Recommendations	Source (reference)	QUADS criteria													Total
		1	2	3	4	5	6	7	8	9	10	11	12	13	
1	(Perreau et al., 2021)	3	3	3	2	0	2	3	3	3	2	3	1	3	31

##### 5.2.2.6 Other Therapies

**Table:** 

Recommendations	Source (reference)	QUADS criteria													Total
		1	2	3	4	5	6	7	8	9	10	11	12	13	
1	(Perreau et al., 2021)	3	3	3	2	0	2	3	3	3	2	3	1	3	31
3	(Vasudevan et al., 2021)	2	2	0	1	0	1	1	1	1	1	1	1	2	14

### 5.3 Genetic Hearing Loss and Gene Therapy for Genetic Hearing Loss

**QuADS Criteria Table for Recommendations (Hearing Loss Genetics)**

**Table:** Second Edition Evidence

Rec.	Source (reference)	1	2	3	4	5	6	7	8	9	10	11	12	13	Total
1	ACMG Hearing Loss Practice Resource (2022); JCIH Position Statement (2019); NHS Genomic Test Directory eligibility criteria (v8.1)	2	2	3	2	2	1	2	1	0	1	2	2	1	21
2	GMC Decision making and consent (2020); JCGM Consent & confidentiality in genomic medicine (2019); ACMG Points to Consider for informed consent in genomic sequencing (2013)	3	3	3	2	1	2	3	1	0	1	2	3	2	26
3	ACMG Hearing Loss Practice Resource (2022); JCGM Consent and confidentiality in genomic medicine (2019); ACMG Points to Consider for informed consent in genomic sequencing (2013)	3	2	3	2	1	2	3	1	0	1	2	3	1	24
4	ACGS UK practice guidelines for variant classification (2024); ACMG Hearing Loss Practice Resource (2022)	3	3	3	2	1	2	3	2	0	2	2	3	2	28
5	ACMG Hearing Loss Practice Resource (2022); Sloan-Heggen et al. large cohort diagnostic yield study (2016); PanelApp consensus gene panel curation paper (2016)	3	2	3	3	2	2	3	3	3	2	3	2	2	33
6	Richards et al. ACMG/AMP standards (2015); ClinGen Hearing Loss Expert Panel specifications (2018); ACGS variant classification guidance (2024); International expert consensus on gene therapy for hereditary hearing loss (2024)	3	3	3	2	2	2	3	2	1	3	3	2	2	31

**QuADS Criteria Table for Recommendations (Gene Therapy—Hereditary Hearing Loss).**

**Table:** Second Edition Evidence

Rec.	Source (reference)	1	2	3	4	5	6	7	8	9	10	11	12	13	Total
1	Fan X et al. International expert consensus on gene therapy for hereditary hearing loss: based on clinical trials. Med 2026;7:100886.; GMC Decision making and consent (2020); JCGM Consent and confidentiality in genomic medicine (2019).	2	3	2	2	1	2	2	1	0	1	2	2	1	21
2	Fan X et al. Med 2026;7:100886.; NEJM DB-OTO trial (AAV gene therapy for DFNB9; 2024).	2	3	2	2	1	2	2	1	0	1	2	2	1	21
3	Fan X et al. Med 2026;7:100886.; NEJM DB-OTO trial staged dosing design (2024).	1	2	1	2	1	1	1	1	0	1	2	3	1	17
4	Fan X et al. Med 2026;7:100886.; JCGM Consent and confidentiality (2019).	1	2	1	2	1	1	1	1	0	1	2	2	1	16
5	ACMG/AMP variant interpretation (Richards et al, 2015); ClinGen HL-EP hearing loss specifications (2018); ACGS UK variant classification guidelines (2024); Fan X et al. Med, 2026;7:100886.	3	3	3	2	2	2	3	2	1	3	3	2	2	31
6	NEJM DB-OTO trial eligibility/phenotype (2024); Fan X et al. Med, 2026;7:100886.	2	3	3	3	2	2	3	2	1	2	3	2	2	30
7	Fan X et al. Med, 2026;7:100886.; NEJM DB-OTO trial baseline imaging/safety/vestibular monitoring (2024).	2	3	2	2	2	2	2	2	1	2	2	2	2	26
8	Fan X et al. Med, 2026;7:100886. (NAb/immune considerations); Principles of inner ear gene therapy reviews (2025).	1	2	1	2	1	2	2	1	0	1	2	2	1	18
9	Fan X et al. Med, 2026;7:100886.; NEJM DB-OTO trial safety assessments (2024).	1	2	1	2	1	2	2	1	0	1	2	2	1	18
10	Principles of gene therapy of the inner ear (Lin & Shibata 2025); NEJM DB-OTO trial (2024).	1	2	1	2	1	1	1	1	0	1	2	2	1	16
11	Fan X et al. Med, 2026;7:100886. (GMP/handling expectations); product-specific CMC/handling requirements are protocol-defined.	1	2	1	2	1	1	1	1	0	1	2	2	1	16
12	Fan X et al. Med, 2026;7:100886. (peri-operative management statements).	1	2	1	2	1	2	2	1	0	1	2	2	1	18
13	Fan X et al. Med, 2026;7:100886. (glucocorticoid statement; acknowledges evidence gaps).	1	2	1	2	1	2	2	1	0	1	2	2	1	18
14	NEJM DB-OTO trial auditory endpoints and follow-up schedule (2024); Fan X et al. Med, 2026;7:100886.	2	3	2	3	2	2	3	2	1	2	3	2	2	29
15	Fan X et al. Med, 2026;7:100886. (minimum safety evaluation incl. shedding, labs); NEJM DB-OTO trial safety monitoring (2024).	2	3	2	3	2	2	3	2	1	2	3	2	2	29
16	NEJM DB-OTO trial AE/SAE reporting and oversight (2024); Fan X et al. Med, 2026;7:100886.	1	2	1	2	1	1	1	1	0	1	2	2	1	16
17	Fan X et al. Med, 2026;7:100886.; NEJM DB-OTO trial follow-up duration framework (2024); guideline recommends ≥ 5 y	1	2	1	2	1	1	1	1	0	1	2	2	1	16

## Ongoing Care and Support

### Ongoing Care and Support: Measurement of Outcomes and Assessing if Treatment Goals Have Been Addressed

**Table:** First Edition Evidence

Rec.	Evidence	Source (reference)	Level	Grade	EF/EV	MM/P/CI
1	Self-report outcome measures with known psychometric properties can be useful for determining the benefits and effectiveness of hearing aids and the impact on the client's quality of life.	Valente et al. (1998)	6	D	–	
1	Many factors need to be considered when measuring outcomes	Saunders et al. (2005)	6	D	–	–
2	Questionnaire data are sensitive to fitting parameters	Valente et al.(2018)	2	A	EF	MM
		Anderson et al. (2018)	2	A	EF	MM
3	For maximum clinical usefulness, the outcome questionnaire should be specifically in the hearing domain, as greater effect sizes are shown	Chisolm et al (2007)	4	B	EV	–
3	Outcome questionnaire should align with ICF core set for hearing loss	Danermark et al. (2014)	6	D	–	–
		Granberg et al. (2014)	6	D	EV	–
4	Chosen questionnaire should have proven reliability, validity, sensitivity, and available normative data	Cox (2005b)	6	D	–	MM
5	Client-reported outcome measures are available that show before and after comparisons of an intervention such as hearing aid fitting	Dillon et al. (1997)	4	B	EV	–
		Gatehouse (1999)	4	B	EV	–
		Cox et al. (2000)	6	D	EV	MM
6	Ecological momentary assessment or similar tools may offer future alternatives to subjective questionnaires for gathering outcome information and are less dependent on subjective recall	Timmer et al. (2017)	4	B	EV	MM

### Ongoing Care and Support: Assessing the Need for Onward Referral

**Table:** 

Rec.	Evidence	Source (reference)	Level	Grade	EF/EV	MM/P/CI
1	Hearing care professionals should become familiar with local criteria for cochlear implants	Chundu & Buhagiar (2013)	4	B	EV	CI
		Carlson et al. (2018)	4	B	EV	
		Vickers et al. (2016)	4	B	EV	
		Raine et al. (2016)	6	D	–	CI
2	Aided speech testing has an important role in screening for candidacy for cochlear implant assessment.	Holder et al. (2018)	3	C	EV	P, CI
2	There are practical challenges in using speech audiometry to measure the differences between rehabilitative interventions.	Green 1997	6	D	–	–
2	Aided speech testing does not correlate well with subjective measures of use and benefit of hearing aids	Parving 1991	4	B	EF	MM
3	Remote microphones aid speech discrimination in noise	Rodemerk & Glaster (2015)	3	B	EF	–
		Kates et al. (2019)	3	B	EF	–
4	Consider referring to an ear, nose and throat service if there is abnormal appearance of the outer ear or eardrum, or if persistent middle ear effusion.	NICE Hearing loss in adults (2018)	6	D	–	–

### Ongoing Care and Support: Ensuring Appropriate On-Going Management

**Table:** 

Rec.	Evidence	Source (reference)	Level	Grade	EF/EV	MM/P/CI
1	Individuals with moderately severe hearing loss had lower self-efficacy for aided listening conditions than individuals with mild hearing loss	Kawaguchi et al. (2019)	4	B	EV	MM
1	Hearing aid management skills and knowledge are associated with better outcomes	Bennett et al. (2018)	4	B	EV	MM
2	Hearing and earmolds should be regularly maintained	Souza (2009)	6	D	–	–
4	Individuals should be seen for regular review	Goggins & Day (2009)	4	C	EV	–
6	Regular review should explore CI candidacy given that one of the reasons for not pursuing a CI is lack of awareness	Holder et al. (2018)	3	C	EV	P, CI
		Turunen-Taheri et al. (2019)	4	B	EV	CI
7	While many hearing care professionals are aware of CI criteria, many reported a lack of training and confidence for discussing CIs and making referrals	Chundu & Buhagiar (2013)	4	B	EV	CI
		Allen et al. (2018)	4	D	EV	CI
7	Lack of health care professional knowledge is one of the barriers to cochlear implant uptake	Bierbaum et al. (2019)	4	B	EV	CI
7	Training can significantly improve knowledge of CI candidacy	Raine et al. (2016)	6	D	–	CI
8	Numbers of adults with severe and profound hearing loss could be as low as less than 7% of a clinic	Turton & Smith (2013)	4	B	EV	–
8	Hearing care professionals prefer training in a variety of formats: in-house training in their local centers, workshops at CI-centered, online training, and training by CI manufacturers	Allen et al. (2018)	4	D	EV	CI
		Davies et al. (2019)	6	D	–	–

## Second Edition Evidence

### 6.1 Measurement of Patient-Reported Outcomes and Assessment of Treatment Goals

**Table:** 

Rec.	Source (reference)	QUADS criteria													
		1	2	3	4	5	6	7	8	9	10	11	12	13	Total
1–4	Boisvert et al., 2020	2	3	2	3	3	3	3	3	0	2	3	0	2	29
1–4	Nijmeijer et al., 2020	2	3	2	3	3	3	3	3	0	3	3	0	3	31
1–4	Olsson et al., 2022	1	1	2	2	1	1	1	2	2	2	1	0	2	18
1–4	Hori et al., 2025	1	2	2	3	3	3	3	3	1	3	3	0	3	30
1–4	Tang et al., 2024	1	1	2	2	2	2	2	3	0	1	2	0	2	20
1–4	Tang et al., 2025	1	1	2	2	2	2	2	3	0	1	2	0	2	20

### 6.2 Assessing the Need for Onward Referral

**Table:** 

Rec.	Source (reference)	QUADS criteria													
		1	2	3	4	5	6	7	8	9	10	11	12	13	Total
1–3	Boisvert et al., 2020	2	3	2	3	3	3	3	3	0	2	3	0	2	29
2, 3	Nijmeijer et al., 2020	2	3	2	3	3	3	3	3	0	3	3	0	3	31
2, 3	Hori et al., 2025	1	2	2	3	3	3	3	3	1	3	3	0	3	30
1, 2	Tang et al., 2024	1	1	2	2	2	2	2	3	0	1	2	0	2	20

### 6.3 Ensuring Appropriate Ongoing Management

**Table:** 

Rec.	Source (reference)	QUADS criteria													
		1	2	3	4	5	6	7	8	9	10	11	12	13	Total
1, 4, 5	Boisvert et al., 2020	2	3	2	3	3	3	3	3	0	2	3	0	2	29
2, 3, 5	Nijmeijer et al., 2020	2	3	2	3	3	3	3	3	0	3	3	0	3	31
2, 3, 5	Olsson et al., 2022	1	1	2	2	1	1	1	2	2	2	1	0	2	18
4, 5	Tang et al., 2024	1	1	2	2	2	2	2	3	0	1	2	0	2	20
2, 4, 5	Tang et al., 2025	1	1	2	2	2	2	2	3	0	1	2	0	2	20

## Appendix 2

### Bibliography

## Appendix 3

### Toolkit Tables

#### Accessibility 1.0

**Table 1:** Supporting accessibility and deaf awareness and sign language

Tool	Reference/link
Deaf awareness training	
BSA online training on deaf awareness (available to members) UCL Deaf Awareness for Audiologists (United Kingdom) NAD range of Deaf Awareness courses suitable for Audiologists (United States)	https://www.thebsa.org.uk/ https://www.ucl.ac.uk/short-courses/search-courses/deaf-awareness-online-training-audiologists https://nationaldeafcenter.org/learn/online-learning/
Deaf awareness advice	
British Society of Audiology recommendations	British Society of Audiology (2025), Guiding Principles of Deaf Awareness in Healthcare Settings (Online). Available from: BSA-Practice-Guidance-Deaf-Awareness-January-2026.pdf Accessed on 16.1.26
British Deaf Association communication tips	https://bda.org.uk/help-resources/#communicating
Communication support and assistive technology	
Visual alerting examples	https://screenstar.net/?msclkid=ba6c35f6b9c81f2c0a10501ec3b39135
Vibrating pager system examples	https://royaldeaf.org.uk/
Loop systems	https://www.hearingdogs.org.uk/support/living/assistive-technology/loops
Remote captioning	https://www.nadp.org.uk/wp-content/uploads/2020/12/Making-your-online-meetings-accessible-for-people-who-are-deaf-or-hard-of-hearing-with-live-captioning.pdf Zoom Teams
Video relay interpreting	https://sorenson.com (United States) https://sorenson.co.uk (United Kingdom) https://www.convo.io (Global—Australia, Canada, South Africa, United Kingdom and United States)
Sign language resources	
Professional Sign Language Interpreter Guidance	https://bda.org.uk/help-resources/#interpreters
UCL—Sign Bank—It has two functions: one as a dictionary (for learners, teachers, interpreters, etc) and another as a lexical database for researchers	https://bslsignbank.ucl.ac.uk/
Resources for patients	
National Association for Deafened People—Medical visit preparation	https://nadp.org.uk/wp-content/uploads/2021/05/Deaf-Hard-of-Hearing-Medical-Handout.pdf

### Assessment 2.0

**Table 2:** Understanding communication needs and the impact of hearing loss

Tool	Reference
Communication needs and treatment goals	
Client-Orientated Scale of Improvement (COSI) a	Dillon H, James A, Ginis J. Client Oriented Scale of Improvement (COSI) and its relationship to several other measures of benefit and satisfaction provided by hearing aids. J Am Acad Audiol 1997;8:27–43
Glasgow Hearing Aid Benefit Profile (GHABP) a	Gatehouse S. Glasgow Hearing Aid Benefit Profile: derivation and validation of client centered outcome measures for hearing aid services. J Am Acad Audiol 1999;10:80–103
Glasgow Hearing Aid Difference Profile (GHADP)	Gatehouse S. Glasgow Hearing Aid Benefit Profile: derivation and validation of client centered outcome measures for hearing aid services. J Am Acad Audiol 1999;10:80–103
Revised Hearing Handicap Inventory (RHHI) a	Cassarly C, Matthews LJ, Simpson AN, Dubno JR. The Revised Hearing Handicap Inventory and screening tool based on psychometric reevaluation of the hearing handicap inventories for the elderly and adults. Ear Hear. 2020;41(1):95
Hearing Handicap Inventory for the Elderly (HHIE) a	Ventry IM, Weinstein BE. The Hearing Handicap Inventory for the Elderly: A new tool. Ear Hear 1982;3(3):128–134
Hearing Handicap Inventory for Adults (HHIA) a	Newman C, Weinstein B, Jacobson G, Hug G. The Hearing Handicap Inventory for Adults: psychometric adequacy and audiometric correlates. Ear Hear 1990;11(6):430–433
Hearing Handicap Questionnaire (HHQ) a	Gatehouse S, Noble W. The speech, spatial and qualities of hearing scale (SSQ). Int J Audiol 2004;43(2):85–99
Quantified Denver Scale of Communication Function (QDS) a	Schow, R.L. & Nerbonne, M.A. 1982. Communication screening profile; use with elderly clients. Ear Hear, 3, 135–147
Self-Assessment of Communication (SAC) a	Schow RL, Nerbonne MA. Communication screening profile: Use with elderly clients. Ear Hear 1982;3(3):135–147
Speech, Spatial and Qualities of Hearing scale (SSQ-12) ^b^	Noble W, Søgaard Jensen N, Naylor G, Bhullar N, Akeroyd M. A short form of the Speech, Spatial and Qualities of Hearing scale suitable for clinical use: The SSQ12. Int J Audiol 2013;52(6):409–41280
Social Participation Restrictions Questionnaire (SPARQ) a	Heffernan E, Maidment D W, Barry JG, Ferguson, M. Refinement and Validation of the Social Participation Restrictions Questionnaire: An Application of Rasch Analysis and Traditional Psychometric Analysis Techniques. Ear Hear 2019;40(2), 328–339
Ida Institute Living Well Tool	Scarinci, N. Tulloch, K. Meyer, C. Ekberg, K. Lind, C. Using an online tool to apply a person-centred approach in audiological rehabilitation: a pilot Study. Audiol Res 2022; 12: 620–634
Measures for communication partners	
The Hearing Impairment Impact-Significant Other Profile (HII-SOP) a	Preminger J, Meeks S. The Hearing Impairment Impact Significant Other Profile (HIT-SOP): a tool to measure hearing loss-related quality of life in spouses of people with hearing loss. J Am Acad Audiol 2012;23(10):807–823
Significant Other Scale for Hearing Disability (SOS-HEAR) a	Scarinci N, Worrall L, Hickson L. The effect of hearing impairment in older people on the spouse: development and psychometric testing of the significant other scale for hearing disability (SOS-HEAR). Int J Audiol 2009;48(10):671–683.
Family Oriented Communication Assessment and Solutions (FOCAS)	Crowhen D, Turnbull B. FOCAS: Family oriented communication assessment and solutions: a new holistic tool for performance hearing needs assessments. Hearing Review. https://www.hearingreview.com/practice-building/focas-family-oriented-communication-assessment-solutions . 2018;20–26
Goal Sharing for Partners (GPS)	Preminger J, Lind C. Assisting communication partners in the setting of treatment goals: the development of the goal sharing for partners strategy. Seminars in Hearing 2012; 33(1):53–64.

a Many measures can also be used to measure the outcomes of interventions by comparing scores prior to intervention with postinterventions scores.

**Table 3:** Useful tools for nonauditory needs assessment

Issue to be assessed	Tool	Reference
Tests that could be used within an audiology clinic		
Cognitive ability	Montreal Cognitive Assessment (MoCA/MoCHA-HI)	Nasreddine Z, Phillips N, Be,dirian V, et al. The Montreal Cognitive Assessment, MoCA: a brief screening tool for mild cognitive impairment. *J Am Geriatr Soc* 2005;0.53(4):695–699
		Lin V, Chung J, Callahan B, et al. Development of cognitive screening test for the severely hearing impaired: hearing-impaired MoCA. *Laryngoscope* 2017;127(S1):S4–S11
	Mini Mental State Examination (MMSE)	Folstein MF, Folstein SE, McHugh PR. “Mini-mental state”: a practical method for grading the cognitive state of patients for the clinician. *J Psychiatr Res* 1975;12:189–198
Mental Health status	HADs Hospital anxiety and depression scale	Zigmond AS, Snaith RP. The hospital anxiety and depression scale. *Acta Psychiatr Scan* 1983;67(6):361–370
General, physical health status and dexterity	Assess general and physical health through careful history taking with particular attention to factors that would affect ability to use and manage devices	NA
Visual status	Assess visual status in history with particular attention on visual ability to see faces for lipreading.	NA
Additional tests likely to require onward referral		
General health	EuroQOL (EQ-5D)	EuroQol Research Foundation. EQ-5D-5L User Guide. https://euroqol . org/publications/user-guides. Updated 2019
	Health Utilities Index (HUI)	Horsman J, Furlong W, Feeny D, Torrance G. The Health Utilities Index (HUI): concepts, measurement properties and applications. *Health Qual Life Out* 2003;1:1–13
	Short Form—36 Health Survey (SF-36)	Ware JE, Sherbourne CD. The MOS 36-Item Short-Form Health Survey (SF-36): I. Conceptual Framework and Item Selection. *Med Care* 1992;30(6):473–483
	Sickness Impact Profile (SIP)	Bergner M, Bobbitt RA, Carter WB, Gilson BS. The sickness impact profile: development and final revision of a health status measure. *Med Care* 1981;19(8):787–805
	World Health Organization Measure of QOL (WHO-QOL)	World Health Organization (WHO). Microsoft Word—95 FT 100 Q's. doc. https://www.who.int/mental_health/evidence/WHOQOL_100.pdf? ua = 1. Updated 1995
Cognition	6CIT Six-item Cognitive Impairment Test	Brooke P, Bullock R. Validation of a 6 item cognitive impairment test with a view to primary care usage. *Int J Geriatr Psychiatry* 1999;14 (11):936–940
	Cambridge Cognitive Examination (CAMCOG: short version of CAMDEX and CAMTAB app)	Huppert FA, Brayne C, Gill C, Paykel ES, Beardsall L. CAMCOG: a concise neuropsychological test to assist dementia diagnosis: socio-demographic determinants in an elderly population sample. *Br J Clin Psychol* 1995;34:529–541
		Cambridge Cognition Ltd. CANTAB app. www.camcog.com Roth M, Tym E, Mountjoy CQ, Huppert FA. CAMDEX: a standardized instrument for the diagnosis of mental disorders in the elderly with special reference to early detection of dementia. *Br J Psychiatry* 1986;149(6):698–709
	Cognitive Status Exam (Cognistat)	Schwamm LH, Van Dyke C, Kiernan RJ, Merrin E, Mueller J. The Neurobehavioral Cognitive Status Examination: comparison with the NCSE and MMSE in a neurosurgical population. *Ann Intern Med* 1987;107(4):486–491
	Kahn-Goldfarb Mental Status Questionnaire (MSQ)	Kane RA, Kane RL. Assessing the elderly: a practical guide to measurement. Lexington, MA: Lexington Books; 1981
	MicroCog Assessment of Cognitive Functioning Computerized Testing Instrument	Powell DH, Kaplan EF, Whitla D, Weintraub S, Catlin R, Funkenstein HH. MicroCog: Assessment of Cognitive Functioning (Version 2.1) (Computer software). The Psychological Corporation, San Antonio, TX; 1993
	Short Portable Mental Status Questionnaire (Short Portable MSQ)	Pfeiffer E. A short portable mental status questionnaire for the assessment of organic brain deficit in elderly patients. *J Am Geriatr Soc* 1975;23(10):433–441
	Wechsler Adult Intelligence Scale (WAIS)	Wechsler, D. The Measurement and Appraisal of Adult Intelligence (4th ed.). Baltimore, MD: Williams & Wilkins; 1958
	Tests for memory: California Verbal Learning Test (CVLT)	Delis DC, Kramer JH, Kaplan E, Ober BA. The California verbal learning test: research edition, adult version. San Antonio, TX: The Psychological Corporation; 1987
	Digit Span Test	Ramsay MC, Reynolds CR. (1995). Separate digits tests: a brief history, a literature review, and a reexamination of the factor structure of the Test of Memory and Learning (TOMAL). *Neuropsychol Rev* , *5* :151–171
	Sentence Span Task	Daneman M, Carpenter PA. (1980). Individual differences in working memory and reading. *J Verbal Learning Verbal Behav* , *19* (4):450–466
	Rey Auditory Verbal Learning Test (AVLT)	Schmidt M. Rey auditory verbal learning test: a handbook. Los Angeles, CA: Western Psychological Services; 1996
	Wechsler Memory Scale-III (WMS-III)	Wechsler D. (1945). A standardised memory scale for clinical use. *J Psychol* , *19* :87–95
	Word Span Task	Conway AR, Kane MJ, Bunting MF, Hambrick DZ, Wilhelm O, Engle RW. (2005). Working memory span tasks: a review and a user's guide. *Psychon Bull Rev* , *12* :769–786
Mental health	Beck Depression Inventory (BDI)	Beck AT, Steer RA, Ball R, Ranieri W. (1996). Comparison of Beck depression inventories—IA and II in psychiatric outpatients. *J Pers Assess* , *67* (3):588–597
	Patient Health Questionnaire (PHQ-9)	Kroenke K, Spitzer RL, Williams JB. (2001). The PHQ-9: validity of a brief depression severity measure. *J Gen Intern Med* , *16* (9):606–613
Manual dexterity	Modified Characteristic of Amplification Tool (COAT)	Sandridge S, Newman C. Improving the efficiency and accountability of the hearing aid selection process—use of the COAT. AudiologyOnline. com.
	Nine-Hole Peg Test of manual dexterity	Feys P, Lamers I, Francis G, et al. (2017). The Nine-Hole Peg Test as a manual dexterity performance measure for multiple sclerosis. *Mult Scler J* , *23* (5):711–720

Note: Some of these tools will not be administered by the hearing care professional but will be used by other healthcare professionals. It is important that the hearing care professional understands the local setup for referrals and the use of these tools.

**Table 4:** Useful tools for obtaining behavioral diagnostic information

Tool	Reference
Tinnitus evaluation tools	
Tinnitus Functional Index	Henry JA, Stewart BJ, Abrams HB, et al. (2014). Tinnitus Functional Index—development and clinical application. *Audiology Today, 26* (6):40–48
Tinnitus Reaction Questionnaire	Wilson PH, Henry J, Bowen M, Haralambous G. (1991). Tinnitus Reaction Questionnaire: Psychometric properties of a measure of distress associated with tinnitus. *J Speech Hear Res, 34* (1) 197–201
Tinnitus Handicap Inventory	Newman CW, Jacobson GP, Spitzer JB. (1996). Development of the Tinnitus Handicap Inventory. *Arch Otolaryngol, 122* (2):143–148
Tinnitus Questionnaire	Hallam RS, Jakes SC, Hinchcliffe R. (1988). Cognitive variables in tinnitus annoyance. *Brit J Clin Psychol, 27* (3):213–222
Tinnitus and Hearing Survey	Henry J, Griest S, Zaugg T, et al. Tinnitus and hearing survey: a screening tool to differentiate bothersome tinnitus from hearing difficulties. *Am J Audiol*
Speech tests	
AB words	Boothroyd A. (1968). Developments in speech audiometry. *Br J Audiol, 2* (1):3–102
AzBio sentence lists (available in multiple languages)	Spahr A, Dorman M, Litvak L, et al. (2012). Development and validation of the AzBio Sentence Lists. *Ear Hear* , *33* (1):112–117
BKB-A sentence lists	Bench J, Kowal A, Bamford J. (1979). The BKB (Bamford-Kowal-Bench) sentence lists for partially-hearing children. *Br J Audiol* , *13* (3):108–12
BKB-SIN test	Niquette P, Arcaroli J, Revit L, et al. Development of the BKB-SIN Test. Paper presented at: American Auditory Society Annual Meeting; 2003; Scottsdale, AZ
CUNY sentence lists	Boothroyd A, Hanin L, Hnath T. A sentence test of speech perception: reliability, set equivalence, and short term learning. CUNY Academic Works. https://academicworks.cuny.edu/cgi/viewcontent.cgi?article=1443&context=gc_pubs . 1985. Accessed February 9, 2019
CNC word lists (available in a range of dialects)	Peterson G, Lehiste I. (1962). Revised CNC Lists for auditory tests. *J Speech Hear Dis* , *27* (1):62–70
HINT sentences (available in multiple languages)	Nilsson M, Soli S, Sullivan J. (1994). Development of the Hearing in Noise Test for the measurement of speech reception thresholds in quiet and in noise. *J Acoust Soc Am* , *95* (2):1085–1099
QuickSIN	Etymotic Research. Quick Speech-in-Noise Test (Version 1.3)—User manual. https://www.etymotic.com/downloads/dl/file/id/259/product/159/quicksin_user_-manual.pdf . Updated 2006
Additional tests	
Threshold equalizing noise test	Moore BC, Huss M, Vickers DA. Glasberg BR, Alcántara JI (2000). A test for the diagnosis of dead regions in the cochlea. *Br J Audiolo, 34(4):205–224*
Audible Contrast Test	Zaar J, Simonsen LB, Sanchez-Lopez R, Laugesen S. (2024). The Audible Contrast Threshold (ACT) test: A clinical spectro–temporal modulation detection test. *Hear Res, 453–1091*

**Table 5:** Useful tools for obtaining objective diagnostic information

Tool	Reference
Objective testing	
Although protocols for objective threshold estimation are generally focused on pediatric assessment, the British Society of Audiology has produced a series of comprehensive guides with specific recommendations for adult testing. These guides include cortical auditory evoked potentials (CAEP), auditory steady-state responses (ASSR), and auditory brainstem responses (ABR). All of the guides are freely available on their website: https://www.thebsa.org.uk/guidance-and-resources/current-guidance/	British Society of Audiology Recommended Procedure Cortical Auditory Evoked Potential (CAEP) Testing (2022) https://www.thebsa.org.uk/wp-content/uploads/2023/10/OD104-40-BSA-Recommended-Procedure-CAEP.pdf British Society of Audiology Practice Guidance Auditory Steady State Response (ASSR) Testing (2023) https://www.thebsa.org.uk/wp-content/uploads/2023/10/OD104-115-Practice-Guidance-for-ASSR-Testing-1.pdf British Society of Audiology Recommended Procedure Auditory Brainstem Response (ABR) testing for Postnewborn and Adult (2019) https://www.thebsa.org.uk/wp-content/uploads/2022/09/ABR-post-newborn-and-Adult.pdf
A guide for assessment and management of auditory neuropathy spectrum disorder in children is available from the British Society of Audiology. Although focused on pediatric assessment, many of the recommendations are also appropriate for adult assessment. This can be downloaded from their website: https://www.thebsa.org.uk/guidance-and-resources/current-guidance/	British Society of Audiology Recommended Procedure Assessment and Management of Auditory Neuropathy Spectrum Disorder (ANSD) in Young Infants (2019) https://www.thebsa.org.uk/wp-content/uploads/2023/10/OD104-85-Recommended-Procedure-Assessment-and-Management-of-ANSD-in-Young-Infants.pdf

**Table 6:** Useful tools for developing a comprehensive treatment plan

Tool	Reference
Individual management plans	
A Usable Interpretation of Individual Management Plans within Adult Rehabilitation Questions and Answers	Appendix 3 in the NHS Scotland: Quality Standards for Adult Hearing Rehabilitation Services—Audiology Services Advisory Group October (2008) http://www.knowledge.scot.nhs.uk/media/CLT/ResourceUploads/4076053/26fbc595-da89-4938-8c3d-a0511b747c2e.pdf
Example of an Individual Management Plan (IMP)	Appendix 5 NHS Scotland: Quality Standards for Adult Hearing Rehabilitation Services—Audiology Services Advisory Group October (2008) http://www.knowledge.scot.nhs.uk/media/CLT/ResourceUploads/4076053/26fbc595-da89-4938-8c3d-a0511b747c2e.pdf

## Technology 4.0

### 4.1 Hearing Aid Fitting

**Table 7:** Useful tools for overall information on aiding people with severe and profound hearing loss

Tool	Reference
Overall information on aiding people with severe and profound hearing loss	
British Academy of Audiology (2025) Guidelines for the definition and management of optimally aided for experienced adult hearing aid users with severe and profound deafness.	Guidelines for the definition and management of “optimally aided” for experienced adult hearing aid users with severe and profound deafness
American Academy of Audiology Clinical Practice Guidelines: Adult Patients with Severe-to-Profound Unilateral Sensorineural Hearing Loss (2015)	https://www.audiology.org/sites/default/files/PractGuidelineAdultsPatientsWithSNHL.pdf

**Table 8:** Useful tools for frequency compression and transposition

Tool	Reference
Frequency compression and transposition	
Frequency lowering fitting assistants	https://web.ics.purdue.edu/~alexan14/fittingassistants.html
UWO Plurals Test	https://www.dslio.com/?page_id=314
Stimuli for verification of frequency lowering using Audioscan probe-microphone systems.	https://www.dslio.com/?page_id=166
British Society of Audiology's Practice Guidance on the verification of hearing devices using probe microphone measurements	https://www.thebsa.org.uk/wp-content/uploads/2018/05/REMS-2018.pdf p.21–24
Phoneme Perception Test (Schmitt et al., 2016)	https://www.phonak.com/en-int/professionals/innovations/phoneme-perception-test

**Table 9:** Useful tools for prescriptions and verification

Tool	Reference
Prescriptions and verification	
Software for NAL-NL2 prescriptive procedure	https://shop.nal.gov.au/epages/nal.sf/en_AU/?ObjectPath=/Shops/nal/Categories/Products/Hearing_Aid_Fitting_Prescriptions
BSA Verification (2018)	British Society of Audiology (2018) Practice Guidance on the verification of hearing devices using probe microphone measurements. Available at https://www.thebsa.org.uk/wp-content/uploads/2018/05/REMS-2018.pdf

**Table 10:** Useful tools for maximum power output and threshold shift

Tool	Reference
Maximum power output and threshold shift	
SoundLog noise dosimeter app for iPhone	This free download, developed at NAL, measures noise levels and calculates noise exposure estimates. https://www.nal.gov.au/products/downloadable-software/soundlog/

**Table 11:** Useful tools for music

Tool	Reference
Music options	
Hearing Aids for Music Project Leeds University, United Kingdom	Resources for hearing care professionals and interested individuals about hearing aids and music Resources—Hearing Aids for Music https://musicandhearingaids.org/
Musicians' Clinics of Canada	Tools, Demos and Publications for Musicians and HCPs about prevention of hearing loss in addition to hearing aids and music Musicians Clinics https://musiciansclinics.com/

### 4.2 Prescribing and Fitting Remote Microphones

**Table 12:** Useful tools for prescribing and fitting remote microphones

Tool	Reference
Prescribing and fitting remote microphones	
Client Oriented Scale of Improvement (COSI)	Dillon, H., James, A. & Ginis, J. (1997) Client Oriented Scale of Improvement (COSI) and its relationship to several other measures of benefit and satisfaction provided by hearing aids. Journal of the American Academy of Audiology. 8:27–43
Goal Sharing for Partners (GPS)	https://idainstitute.com/tools/communication_partners/goal_sharing_for_partners/
Family Oriented Communication Assessment and Solutions (FOCAS)	Crowhen, D. & Turnbull, B. (2018). FOCAS: Family Oriented Communication Assessment and Solutions A new holistic tool for performance hearing needs assessments. Hearing Review, November: 20–26: http://www.hearingreview.com/2018/10/focas-family-oriented-communication-assessment-solutions/
TELEGRAM (Telephone, Employment, Legislation, Entertainment, Groups, Recreation, Alarms and Members of the family	Thibodeau, L. (2004). Maximizing communication via hearing assistance technology: Plotting beyond the audiogram! *Hearing Journal, 57* (11), 46–51

**Table 13:** Useful tools for remote microphone verification

Tool	Reference
Verification of remote microphones	
TELEGRAM (Telephone, Employment, Legislation, Entertainment, Groups, Recreation, Alarms and Members of the family	Thibodeau, L. (2004). Maximizing communication via hearing assistance technology: Plotting beyond the audiogram! *Hearing Journal, 57* (11), 46–51
Client Oriented Scale of Improvement (COSI)	Dillon, H., James, A. & Ginis, J. (1997) Client Oriented Scale of Improvement (COSI) and its relationship to several other measures of benefit and satisfaction provided by hearing aids. *Journal of the American Academy of Audiology, 8* :27–43
Goal Sharing for Partners (GPS)	https://idainstitute.com/tools/communication_partners/goal_sharing_for_partners/

### 4.1 Relevant Mobile Apps

**Table 14:** Useful tools for mobile apps

Tool	Reference
Mobile apps	
Speech-to-text apps	The BSA has released an informative guide to what is known about STT apps: https://www.thebsa.org.uk/wp-content/uploads/2023/10/April-2024.pdf
List of useful hearing-related apps	https://www.hearingtracker.com/resources/10-best-hearing-loss-apps-for-smartphones

### 4.2 Alerting Devices and Other Assistive Listening Solutions

**Table 15:** Useful tools for alerting devices and other assistive devices

Tool	Reference
Selecting and using appropriate assistive listening device solutions	
Client Oriented Scale of Improvement (COSI)	Dillon, H., James, A. & Ginis, J. (1997). Client Oriented Scale of Improvement (COSI) and its relationship to several other measures of benefit and satisfaction provided by hearing aids. *Journal of the American Academy of Audiology, 8* :27–43
Goal Sharing for Partners (GPS)	https://idainstitute.com/tools/communication_partners/goal_sharing_for_partners/
Family Oriented Communication Assessment and Solutions (FOCAS)	Crowhen, D. & Turnbull, B. (2018). FOCAS: Family Oriented Communication Assessment and Solutions A new holistic tool for performance hearing needs assessments. Hearing Review, November: 20–26 http://www.hearingreview.com/2018/10/focas-family-oriented-communication-assessment-solutions/
TELEGRAM (Telephone, Employment, Legislation, Entertainment, Groups, Recreation, Alarms and Members of the family	Thibodeau, L. (2004). Maximizing communication via hearing assistance technology: Plotting beyond the audiogram! *Hearing Journal, 57* (11), 46–51
Tools	
Apps such as LoopFinder, which are emerging in the United States, have great potential, currently with very limited geographic coverage.	HLAA website https://www.loopfinder.com/ Accessed on 4.12.2019
U.S. ABLEDATA: database	https://abledata.acl.gov Tools and Technologies to Enhance Life
EU EASTIN: database	European assistive technology information network at www.eastin.eu
AU NED: database	https://ilcaustralia.org.au/ Independent Living Centres Australia National Equipment Database
CA ORTC: organization	Ontario Rehabilitation Technology Consortium (Canada)
Live performance	http://www.stagetext.org/about-stagetext/info-and-services/captions-subtitles-and-surtitles
Assistance animals	
Find your local hearing dog provider	https://www.hearingdogs.org.uk/
	https://www.pawswithacause.org/what-we-do/assistance-dogs/hearing-dogs/
	https://www.akc.org/expert-advice/training/hearing-dogs/
	https://www.healthyhearing.com/report/52110-Assistance-dogs-for-the-deaf

### 4.3 Auracast

**Table 16:** Useful tools for Public Broadcast Protocols (Auracast)

Tool	Reference
**Auracast**	
RNID: Report on Auracast	Report published November, 2025. Link: https://rnid.org.uk/wp-content/uploads/2025/02/Final-Auracast-Report-110225.pdf
ABI Research and Bluetooth SIG: Auracast Broadcast Audio Retrofit Solutions and Opportunities	Whitepaper published March, 2024. link: https://go.abiresearch.com/hubfs/Marketing/Whitepapers/Auracast%20Broadcast%20Audio%20Retrofit%20Solutions%20and%20Opportunities/ABI_Research%20Auracast%20Broadcast%20Audio%20Retrofit%20Solutions%20and%20Opportunities.pdf?hsCtaTracking=4c37e4ce-37c6-46a8-a7 b4-71a35cecbd04%7Cae79dfc9-7c90-443c-b3cc-0780de5b5bfe

### 4.4 Cochlear Implant Candidacy and Bimodal Hearing

**Table 17:** Useful tools for starting the cochlear implant conversation with clients

Tool	Reference
**Starting the cochlear implant conversation with clients**	
Website for candidates, professionals and families to inform about Cochlear Implant referral and access	Cochlear Implant Champions—British Academy of Audiology MAGICapp—Making GRADE the Irresistible Choice—Guidelines and Recommendations
An information leaflet from your local cochlear implant center	
A demo implant and speech processor, available on request from manufacturers	
A global network, aiming to close the gap in Cochlear Implant provision; ensuring lifelong support for all who could benefit.	CIICA—Cochlear Implant International Community of Action
Local recipient groups	

**Table 18:** Understanding the benefits of bimodal fittings

Tool	Reference
**Bimodal**	
Guidelines for Best Practice in the Audiological Management of Adults Using Bimodal Hearing Configurations	https://adulthearing.com/wp-content/uploads/2022/07/Guidelines-for-Best-Practice-in-the-Audiological-Management-of-Adults.pdf

**Table 19:** Understanding the limitations of other implantable devices for this population

Tool	Reference
ABI	
NICE Auditory brain stem implants HealthTech guidance	https://www.nice.org.uk/guidance/htg65

### 4.5 Remote Fittings

**Table 20:** Understanding remote fittings

Tool	Reference
**Remote fittings**	
ASHA Telepractice Practice Portal—Comprehensive guidance on delivering telehealth services in audiology, covering patient selection, platform requirements, ethical considerations, and clinical protocols	https://www.asha.org/practice-portal/professional-issues/telepractice/
Audiology Australia Teleaudiology Guidelines (2022)—National guidelines for teleaudiology service delivery in Australia, including hearing aid fitting, cochlear implant follow-up, outcome monitoring, and technology requirements	https://audiology.asn.au/standards-guidelines/teleaudiology-guidelines/
BAA/BSA Guidance on Remote Consultation/Tele-Audiology (2021)—UK guidance covering clinical standards, governance, consent, technology requirements, and safeguards for remote audiological service delivery	British Academy of Audiology and British Society of Audiology. Guidance on the Use of Remote Consultation/Tele-Audiology. 2021
Swanepoel DW. Teleaudiology—Book chapter providing evidence-based guidance on remote hearing aid fitting practice, candidacy selection, clinical workflow, and safety considerations for severe-to-profound hearing loss	Swanepoel DW. Teleaudiology. In: Katz J, Burkard R, Medwetsky L, Hood L, eds. Handbook of Clinical Audiology. 8th ed. Wolters Kluwer; in press

### 5.1 Rehabilitation: Communication and Psychosocial Concerns

**Table 21:** Useful Tools for exploring and addressing the psychosocial impact of the severe and profound hearing loss

Tool	Reference
**Addressing the psychosocial impact of the severe and profound hearing loss**	
Patient Health Questionniare-4 (PHQ4)	Kroenke, K., R. L. Spitzer, J. B. Williams, and B. Löwe. 2009. An Ultra-Brief Screening Scale for Anxiety and Depression: The PHQ-4. *Psychosomatics* , 50 (6): 613–621. doi:10.1176/appi.psy.50.6.613
Acceptance and Action Questionnaire—Adult Hearing Loss (AAQ-AHL)	Ong, C. W., J. J. Whicker, K. Munoz, and M. P. Twohig. 2019. Measuring Psychological Inflexibility in Adult and Child Hearing Loss. *International Journal of Audiology,* 58 (10): 643–650. doi:10.1080/14992027.2019.1630759
Social Isolation Measure (SIM)	Heffernan, E., A. Habib, and M. Ferguson. 2019. Evaluation of the Psychometric Properties of The Social Isolation Measure (SIM) in Adults with Hearing Loss. *International Journal of Audiology,* 58 (1): 45–52. doi:10.1080/14992027.2018.1533257
Hearing Handicap Inventory for the Elderly (HHIE-S)	Ventry, I. M., and B. E. Weinstein. (1983). Identification of Elderly People with Hearing Problems. *ASHA, 25* (7): 37–42
Revised Hearing Handicap Inventory (RHHI)	Cassarly, C., Matthews, L. J., Simpson, A. N., & Dubno, J. R. (2020). The Revised Hearing Handicap Inventory and Screening Tool Based on Psychometric Reevaluation of the Hearing Handicap Inventories for the Elderly and Adults. *Ear and Hearing* , *41* (1), 95–105. https://doi.org/10.1097/AUD.0000000000000746
My Hearing PREM-16, and My Hearing PREM-9	Smith et al. (2025). Refinement and Validation of a New Patient-Reported Experience Measure for Hearing Loss (My Hearing PREM). *Health Expectations* , 28:e70225. https://doi.org/10.1111/hex.70225
Client Oriented Scale of Improvement (COSI)	Dillon, H., A. James, and J. Ginis. (1997). The Client Oriented Scale of Improvement (COSI) and its Relationship to Several Other Measures of Benefit and Satisfaction Provided by Hearing Aids. *Journal of the American Academy of Audiology* , *8* (2): 27–43
Motivation Tools (the line, the box, and the circle)	https://idainstitute.com/tools/motivation_tools/?tx_idatoolbox_-toolboxpagelist%5Bcontroller% 5D = Toolbox&cHash = 0d5d18956ebeaf1aef89cf06d78f3350
Expected Consequences of Hearing Aid Ownership (ECHO)	Cox RM, Alexander GC. (2000). Expectations about hearing aids and their relationship to fitting outcome. *J Am Acad Audiol, 11* :368–382
Characteristic of Amplification Tool (COAT)	Sandridge S, Newman C. Improving the efficiency and accountability of the hearing aid selection process—use of the COAT. AudiologyOnline.com. https://www.audiologyonline.com/articles/improving-efficiency-and-accountability-hearing-995 . 2006. Accessed February 9, 2019

#### 5.1.2 Incorporating Family in Rehabilitation for Shared Psychosocial Well-Being

**Table 22:** Useful tools to support family-centered care

Tool	Reference
Useful tools to support family-centers care	
The Hearing Impairment Impact—Significant Other Profile (HII-SOP)	Preminger J, Meeks S. (2012). The hearing impairment impact significant other profile (HII-SOP): a tool to measure hearing loss-related quality of life in spouses of people with hearing loss. *J Am Acad Audiol, 23* (10):807–823
Significant Other Scale for Hearing Disability (SOS-HEAR)	Scarinci N, Worrall L, Hickson L. (2009). The effect of hearing impairment in older people on the spouse: development and psychometric testing of the Significant Other Scale for Hearing Disability (SOS-HEAR). *Int J Audiol, 48* (10):671–683
Goal Sharing for Partners Strategy (GPS)	Preminger, J. E., and C. Lind. (2012). Assisting Communication Partners in the Setting of Treatment Goals: The Development of the Goal Sharing for Partners Strategy. *Seminars in Hearing, 33* (01): 053–064
Family Oriented Communication Assessment and Solutions (FOCAS)	Crowhen D, Turnbull B. FOCAS: Family oriented communication assessment and solutions: a new holistic tool for performance hearing needs assessments. Hearing Review. https://www . hearingreview.com/practice-building/focas-family-oriented-communication-assessment-solutions
Affiliate Stigma Scale	Chang, C.C., J.A. Su, C.S. Tsai, C.F. Yen, J.H. Liu, and C.Y. Lin. (2015). “Rasch Analysis Suggested Three Unidimensional Domains for Affiliate Stigma Scale: Additional Psychometric Evaluation.” *Journal of Clinical Epidemiology 68* (6): 674–683. https://doi.org/10.1016/j.jclinepi.2015.01.018

### 5.2 Tinnitus

See also Table 4.

**Table 23:** Useful tools to support tinnitus management

Tool	Reference
**Tinnitus**	
American Tinnitus Association (ATA) Provides clinical guidance, patient resources, research funding information, and professional updates	https://www.ata.org
Tinnitus UK Provides support, education, and evidence–based guidance for both patients and clinicians	https://tinnitus.org.uk
Tinnitus Research Initiative Supports clinicians and researchers with diagnostic tools, guidelines, educational materials, and collaboration platforms	https://www.tinnitusresearch.net

Note: Many countries also have specific tinnitus support organizations. Those listed here have many online resources.

### 5.3 Genetic Hearing Loss and Gene Therapy for Genetic Hearing Loss

**Table 24:** Useful tools for genetic testing and genetic diagnosis (genetic hearing loss)

Tool	Reference/link
Test eligibility and commissioning pathways (United Kingdom)	https://www.england.nhs.uk/publication/national-genomic-test-directories/NHS England: National Genomic Test Directories: https://www.england.nhs.uk/publication/national-genomic-test-directories/ NHS Genomics Education Programme: R67 guidance (nonsyndromic hearing loss): https://www.genomicseducation.hee.nhs.uk/genotes/in-the-clinic/presentation-child-with-non-syndromic-hearing-loss/ Orphanet (Europe): rare disease info, expert centers and diagnostics: https://www.orpha.net/ NIH Genetic Testing Registry (GTR): find laboratories offering hearing loss tests: https://www.ncbi.nlm.nih.gov/gtr European Commission: European Reference Networks (ERNs) for rare/complex disease care: https://health.ec.europa.eu/rare-diseases-and-european-reference-networks/european-reference-networks_en
Curated hearing loss gene lists and panels	https://panelapp.genomicsengland.co.uk/Genomics England PanelApp: https://panelapp.genomicsengland.co.uk/ Panel 126: Monogenic hearing loss: https://panelapp.genomicsengland.co.uk/panels/126/ Hereditary Hearing Loss Homepage: curated gene/locus list: https://hereditaryhearingloss.org/ Deafness Variation Database (DVD): expert-curated hearing loss variants/genes: https://deafnessvariationdatabase.org/ ClinGen Hearing Loss Clinical Domain Working Group: gene–disease validity: https://clinicalgenome.org/working-groups/clinical-domain/hearing-loss/
Genetic testing approaches in hearing loss (clinical resource)	ACMG Practice Resource (2022): Clinical evaluation and etiologic diagnosis of hearing loss: https://doi.org/10.1016/j.gim.2022.03.018
Variant interpretation standards	ACMG/AMP standards (Richards et al, 2015): https://doi.org/10.1038/gim.2015.30 ClinGen HL-EP hearing loss specifications (Oza et al, 2018): https://doi.org/10.1002/humu.23630 ACGS Variant Classification (2024 v1.2): https://www.genomicseducation.hee.nhs.uk/wp-content/uploads/2024/08/ACGS-2024_UK-practice-guidelines-for-variant-classification.pdf
Public variant/population databases for interpretation	ClinVar: https://www.ncbi.nlm.nih.gov/clinvar/ gnomAD: https://gnomad.broadinstitute.org/ DECIPHER: https://www.deciphergenomics.org/ OMIM: https://www.omim.org/
Phenotype coding and gene–phenotype resources	Human Phenotype Ontology (HPO): https://hpo.jax.org/ ClinGen: https://clinicalgenome.org/ GeneReviews: https://www.ncbi.nlm.nih.gov/books/NBK1116/
Secondary/incidental findings (ES/GS)	ACMG SF v3.0 list (2021): https://doi.org/10.1038/s41436-021-01172-3
Consent, confidentiality, and family communication	GMC: Decision making and consent: https://www.gmc-uk.org/professional-standards/the-professional-standards/decision-making-and-consent JCGM: Consent and confidentiality in genomic medicine (2019): https://bsgm.org.uk/media/11525/consent_confidentiality_working_report_final_online_2019.pdf ACMG: Points to consider for informed consent for genome/exome sequencing (2013): https://doi.org/10.1038/gim.2013.94

**Table 25:** Useful tools for gene therapy for genetic hearing loss

Tool	Reference/link
Consensus guidance for trials and patient management	International expert consensus (Fan et al, Med 2026): https://doi.org/10.1016/j.medj.2025.100886
Key OTOF (DFNB9) clinical trial publications	DB-OTO (NEJM 2025): https://doi.org/10.1056/NEJMoa2400521 AAV-OTOF single-arm trial (Nature Medicine 2025): https://doi.org/10.1038/s41591-025-03773-w AAV-OTOF first-in-patient report (Adv Sci 2024): https://doi.org/10.1002/advs.202306788
Clinician-friendly reviews and background	Principles of gene therapy of the inner ear (Curr Opin 2025): https://doi.org/10.1097/MOO.0000000000001067 Updates and remaining challenges (Audiol Res 2023): https://doi.org/10.3390/audiolres13060083 Gene therapy for hereditary hearing loss (Hear Res 2025): https://doi.org/10.1016/j.heares.2024.109151 Targeted delivery review (J Drug Target 2023): https://doi.org/10.1080/1061186X.2023.2216900
Trial registries (identify active and recruiting studies)	ClinicalTrials.gov search: https://clinicaltrials.gov/search?term=hearing%20loss%20gene%20therapy EU Clinical Trials Register: https://www.clinicaltrialsregister.eu/ WHO ICTRP trial search: https://trialsearch.who.int/
Long-term follow-up and pharmacovigilance frameworks	FDA LTFU guidance (Jan 2020): https://www.fda.gov/regulatory-information/search-fda-guidance-documents/long-term-follow-after-administration-human-gene-therapy-products EMA guideline on follow-up after gene therapy: https://www.ema.europa.eu/en/documents/scientific-guideline/guideline-follow-patients-administered-gene-therapy-medicinal-products_en.pdf
Adverse event reporting (United Kingdom)	MHRA Yellow Card scheme: https://yellowcard.mhra.gov.uk/

## 6. Ongoing Care and Support

See Tables 2 and 4 .

## Appendix 4

## Clinical Checklists

**Table:** 1. Assessment Management

2. Communication access and cultural competence ( ) Preferred language and communication mode confirmed (e.g., sign language, spoken language, written language, bimodal communication). ( ) Asked whether communication support or adjustments are required (e.g., sign language or spoken language interpreter, speech–to–text). ( ) Communication support arranged in advance when needed. ( ) All interactions delivered in a person-centered, culturally and linguistically sensitive manner. ( ) Clinician acknowledges client as the expert in their lived experience and their communication preferences ( ) Awareness that sign language use reflects linguistic and cultural identity, not simply a communication aid. 3. Communication needs analysis ( ) Open-ended questions used to explore communication challenges. ( ) At least one validated self-report measure completed (e.g., HHIE, SSQ, COSI, GHABP). ( ) Communication needs assessed in the client's preferred language, using culturally adapted tools where possible. ( ) Baseline measures recorded for future outcome tracking. 4. Social and psychosocial history ( ) Social history taken (living situation, employment, social interactions, hobbies). ( ) Deaf identity, language background, and connection to Deaf or signing communities explored where relevant ( ) Consideration of social isolation or reduced participation. ( ) Impact on significant others assessed (e.g., SOHI, SOS–HEAR). ( ) Impact of communication barriers in healthcare, education, or employment discussed. 5. Comorbidities and nonauditory factors ( ) Cognitive ability reviewed. ( ) Mental health status explored. ( ) Physical status assessed (mobility, craniofacial factors). ( ) General health factors noted. ( ) Dexterity assessed. ( ) Visual status assessed. ( ) Additional tests completed if within scope, or onward referral arranged. 6. Otological and medical history ( ) Chronological otological history taken from onset to present. ( ) For reassessments, changes in hearing or tinnitus documented. ( ) Sudden severe/profound hearing loss or acute tinnitus identified and urgently referred to ENT. 7. Diagnostic audiology ( ) Thresholds measured for degree and type of hearing loss. ( ) Uncomfortable loudness levels measured to determine dynamic range. ( ) Speech recognition testing completed (auditory, auditory-visual, conversational as appropriate). ( ) Speech-in-noise or STM testing completed to assess need for additional features or remote microphones. ( ) Cochlear dead region testing considered when relevant. ( ) Diagnostic findings explained using accessible, visual, and/or signed communication methods. 8. Treatment discussions and planning ( ) Transparent discussion of amplification benefits and limitations. ( ) Treatment options considered beyond amplification (communication strategies, environmental changes, support services). ( ) Cochlear implants discussed as an opportunity to explore alternative interventions, respecting individual communication choices and cultural perspectives. ( ) Client's language preference and cultural identity considered when discussing amplification or implant options. ( ) Goals developed collaboratively with the client and, where appropriate, their communication partner. ( ) Personalized care plan created, documented, shared with the client, and updated regularly. 9. Referrals and ongoing support ( ) Referrals made for nonauditory needs (medical, psychological, social). ( ) Referrals made for vision, cognition, dexterity, or mental health assessment when indicated. ( ) Referrals made for cochlear implant assessment when criteria met. ( ) Referral to Deaf-aware or sign language accessible services made where available ( ) Information about Deaf community organizations or peer support shared where appropriate

**Table:** 

Checklist items not met	Actions required	Responsible Person	Resources needed	Deadline	Status
					
					
					
					
					
					

## 2. Hearing Aid Management

**Table:** 

1. Amplification strategy ( ) Multichannel WDRC used (not linear amplification). ( ) Lowest effective compression ratio selected. ( ) Number of channels sufficient for frequency gain shaping, noise reduction, and feedback control. ( ) Slow–moderate or adaptive WDRC used unless fast-acting WDRC is specifically justified. ( ) Transition from linear → WDRC supported with counseling and stepped adjustments. ( ) Speech enhancement algorithms enabled when beneficial. 2. Directionality, noise management and accessories ( ) Bilateral fitting provided when feasible. ( ) Automatic/adaptive directionality activated. ( ) Binaural directionality used when appropriate. ( ) Digital noise reduction enabled. ( ) Remote microphone access available and easy to activate. ( ) Active digital feedback management engaged and verified. ( ) Custom earmolds with appropriate venting used. ( ) Binaural wireless streaming (telecoil/Bluetooth) enabled when relevant. 3. Frequency lowering ( ) Frequency lowering initially OFF; activated only if needed. ( ) If used, frequency compression preferred over transposition (limited evidence). ( ) Fitted using validated procedures at minimum effective strength. 4. Prescriptive fitting and verification ( ) Validated prescriptive method used. ( ) Probe microphone or coupler measures completed. ( ) Adjustments maintain adequate speech audibility; counseling provided if gain increased. ( ) Speech-based stimuli used for verification (not tones/static noise). ( ) Gain increased appropriately for mixed losses (using BC thresholds). ( ) Gain in dead region frequencies reduced only if speech quality or comfort is poor. 5. BiCROS (for asymmetric loss) ( ) BiCROS considered when one ear has minimal usable hearing. ( ) Client trials aids with and without transmitter in real-world settings. ( ) Real ear response measured with/without transmitter; transmitter adjusted accordingly. ( ) Adaptive directionality enabled on both transmitter and receiver. ( ) Client trained on disabling transmitter mic when needed. 6. Output and safety ( ) Maximum output measured as real ear sensation levels (narrowband signals). ( ) Compression limiting used (not peak clipping). ( ) Frequency-specific MPO adjustments applied. ( ) Volume control limits set when client is at risk of unsafe sound exposure. 7. Music ( ) Add a dedicated listening program (or programs) for music depending on the listening goals of the client. ( ) Compression parameters set similarly to what is used for speech. REM should be performed if the gain and MPO are modified to ensure comfort. ( ) Frequency response should be adjusted to the client's preferences; a good first step is potentially a low-frequency gain increase ( ) Noise reduction including wind noise reduction or transient noise management should be turned off or reduced if possible ( ) Use a fixed (nonadapting) microphone setting which is either directional or omnidirectional ( ) Adaptive feedback management should be off if possible, and if needed, the gain should be reduced to minimize risk of feedback. ( ) Frequency lowering should be off or reduced in strength if needed for audibility

**Table:** 

Checklist items not met	Actions required	Responsible Person	Resources needed	Deadline	Status
					
					
					
					
					
					

## 3. Other Technology Options—Remote Microphones

**Table:** 

**Remote Microphones** 1. Client identification and candidacy ( ) Confirm severe–profound hearing loss and discuss RM benefit beyond local microphones. ( ) Review RM candidacy proactively at follow–ups. 2. Communication partner involvement ( ) Identify communication partners and discuss Third Party Disability. ( ) Include partners in information–giving, decision–making, fitting, and reviews. 3. Trial process ( ) Provide a structured RM trial for client and partner. ( ) Collect outcome measures during/after trial. 4. Goal setting ( ) Set functional goals for RM use with client and partner. ( ) Review and update goals regularly. 5. Education and support ( ) Provide clear instructions in multiple formats. ( ) Offer ongoing training and troubleshooting support. ( ) Simplify system complexity wherever possible. 6. Community awareness ( ) Provide information on wider community resources and awareness initiatives. 7. System features ( ) Confirm wireless connectivity to relevant devices (phone, TV, computer, vehicle audio, loops). ( ) Recommend minimal components for reliability and cost–efficiency. ( ) Ensure battery life and charging meet lifestyle needs. ( ) Check compatibility with workplace/education assistive tech. ( ) Fit bilaterally unless contraindicated. 8. Prefitting checks ( ) Verify hearing aid function using best–practice protocols. 9. Electroacoustic verification ( ) RM output does not exceed HA output. ( ) No significant added circuit noise. ( ) No increase in distortion (ANSI S3.47). ( ) HA vs. HA + RM frequency response within ± 5 dB. ( ) Document all verification results. 10. Functional performance ( ) Demonstrate improved performance with RM using AAA (2011) method. ( ) Record client and partner–reported benefit 11. Follow–up ( ) Review RM use, benefit, and device function proactively. ( ) Refresh training and update goals as needed

**Table:** 

Checklist items not met	Actions required	Responsible Person	Resources needed	Deadline	Status
					
					
					
					
					
					

## 4. Other Technology Options—Mobile Apps

**Table:** 

**Relevant mobile apps** 1. Client identification and candidacy ( ) Confirm client's interest in and ability to manage mobile apps. ( ) Provide a list of suitable apps to support hearing, device, and other needs. 2. Maintain knowledge ( ) Stay up to date on suitable apps related to hearing solutions, support, and assistive tools for clients with severe and profound hearing loss.

**Table:** 

Checklist items not met	Actions required	Responsible Person	Resources needed	Deadline	Status
					
					
					
					
					
					

## 5. Other Technology Options—Alerting Devices and ALDs

**Table:** 

**Alerting devices** and **other assistive solutions** Do you/does your clinic: 1. Explore client needs ( ) Identify key listening situations and activities avoided due to hearing loss and consider the implications of other assistive devices alongside hearing aids. 2. Maintain knowledge ( ) Stay up to date on assistive solutions: loops, alerting devices, Bluetooth/Wi–Fi audio, captions/text systems, mobile–based tools. ( ) Keep up to date with local authority or other financial assistance schemes. 3. T–coil and loop access ( ) Activate t–coil when appropriate. ( ) Ensure the client experiences a functioning loop system. 4. Trial opportunities ( ) Provide trials of relevant devices, ideally in real–world settings. 5. Local partnerships ( ) Build links with local charities/sensory services for equipment and advice. 6. Assistance animals ( ) Offer information and signpost eligibility where relevant

**Table:** 

Checklist items not met	Actions required	Responsible Person	Resources needed	Deadline	Status
					
					
					
					
					
					

## 6. Other Technology Options—Auracast

**Table:** 

**Auracast** 1. Client needs and device selection ( ) Consider the client's public accessibility needs in the near-term and whether the hearing technology chosen will meet their needs. ( ) Check the hearing aid technology chosen maximizes accessibility in public spaces, including access to Auracast, telecoil, and/or other wireless protocols (either direct or via an intermediary device)

**Table:** 

Checklist items not met	Actions required	Responsible Person	Resources needed	Deadline	Status
					
					
					
					
					
					

## 7. Other Technology Options—Cochlear Implants (and Other Implantable Devices)

**Table:** 

**Cochlear implants and other implantable devices** 1. Optimize hearing aid fitting ( ) Ensure hearing aids are optimally fitted. ( ) Prescribe remote microphones or other assistive devices when appropriate. 2. Early CI consideration ( ) Discuss CI referral well before hearing aids reach the point of limited benefit. ( ) Reinforce that hearing aids are not the final stage of hearing loss management. 3. Referral purpose ( ) Explain that referral is for information–gathering, not a candidacy decision. ( ) Clarify that candidacy is determined by a multidisciplinary CI team. 4. Clear referral pathway ( ) Maintain a simple, well–understood CI referral pathway. ( ) Build relationships with local CI centers and stay informed about their processes. 5. Ongoing conversations ( ) Revisit CI discussions regularly throughout the client journey. ( ) Treat CI consideration as a process, not a one–off conversation. 6. Continued hearing aid use ( ) Remember that CI candidates and recipients may still require hearing aid care if bimodally fitted ( ) Encourage continued hearing aid use where appropriate. ( ) Monitor and adjust hearing aids based on the client's hearing with the CI

**Table:** 

Checklist items not met	Actions required	Responsible Person	Resources needed	Deadline	Status
					
					
					
					
					
					

## 8. Other Technology Options—Remote Fittings

**Table:** 

**Remote fittings** Initial fitting 1. Gold standard ( ) Prioritize in–person fitting with probe microphone verification. ( ) Use remote initial fitting only when in–person access is not possible, and safeguards are in place (recent audiogram, measured RECD, technical support). 2. Remote initial fitting requirements ( ) Apply conservative MPO settings. ( ) Conduct formal loudness comfort checks. ( ) Include CI candidacy screening. 3. Verification alternatives ( ) Use coupler–based verification with measured RECD if probe mic unavailable. ( ) Avoid average RECD values due to risk of mis–amplification. Remote fine–tuning and follow–up 4. Routine remote follow–up ( ) Offer synchronous or asynchronous remote fine–tuning after an in–person initial fitting. ( ) Note that outcomes are comparable to in–clinic visits. 5. Remote verification and PROMs ( ) Use in–situ speech mapping where available. ( ) Supplement with validated PROMs (e.g., COSI, SSQ￼12, SADL, IOI￼HA, HAUQ, APHAB). 6. Accessory optimization ( ) Optimize remote microphones and wireless accessories during follow–up. 7. Structured follow–up intervals ( ) Maintain scheduled reviews (e.g., 2 wk, 4–6 wk, quarterly in year 1). ( ) Monitor for CI candidacy. 8. Community–based support ( ) Use trained facilitators in underserved settings, with audiologists retaining responsibility for programming and verification. 9. Training and preparation ( ) Ensure clinicians and patients receive training for remote fine–tuning and troubleshooting. ( ) Support shared decision–making. 10. Remote renewal ( ) Consider remote renewal for stable, experienced users with existing audiograms. ( ) Reserve in–person review for new symptoms or insufficient benefit

**Table:** 

Checklist items not met	Actions required	Responsible Person	Resources needed	Deadline	Status
					
					
					
					
					
					

## 9. Rehabilitation Options

**Table:** 

**Rehabilitation** 1. Accessible, inclusive communication ( ) Confirm clients can follow discussions using their preferred mode (spoken, signed, written, captioned). ( ) Use clear, individualized language and verify understanding. ( ) Be familiar with local communication support (sign language interpreters, apps, typed notes). 2. Personal adjustment and psychosocial assessment ( ) Explore communication challenges, strengths, stigma, identity changes, support networks, and health factors. ( ) Assess psychosocial impact using questioning and validated tools; use sign language-adapted versions where available (e.g., PHQ–4, AAQ–AHL, SIM, HHIE–S/RHHI, PREM–16/9). ( ) Ask about stigma (e.g., “Who do you tell about your hearing loss?”). 3. Individualized management planning ( ) Create a plan including needs, actions, completed steps, and outcomes (e.g., COSI–based). ( ) Integrate hearing and socio–emotional goals into device and rehabilitation recommendations. ( ) Provide options using a holistic, empowering, person-centered approach. 4. Motivation and self–management ( ) Use motivational engagement strategies to build readiness and self–efficacy. ( ) Teach self–management skills (e.g., communication repair, lipreading, environmental adjustments, sign language strategies where appropriate). ( ) Review and refine goals regularly; manage expectations collaboratively. 5. Mental health and well–being ( ) Screen for mental health needs early; refer when appropriate. ( ) Use structured approaches such as AIMER to support well–being. 6. Third–party disability and family involvement ( ) Assess impact on family/partners as part of needs assessment. ( ) Invite family to appointments and involve them actively. ( ) Screen for third–party disability (HII–SOP, SOS–HEAR) and affiliate stigma. ( ) Include family support and device–management guidance in the care plan. ( ) Engage family in shared goal–setting and decision–making. ( ) Measure outcomes for family members (SOAC, IOI–HA–SO). 7. Social support and skills development ( ) Assess social support needs (e.g., Communication Rings, SPaRQ). ( ) Offer communication and social skills training (lipreading, sign language skills, auditory training, assertiveness, advocacy). ( ) Provide social coaching and consider group programs (e.g., ACE). 8. Monitoring and workplace support ( ) Monitor for distress, withdrawal, or loneliness; refer when needed. ( ) Discuss workplace accommodations; encourage “buddy systems” or communication champions. ( ) Provide advocacy coaching for requesting reasonable adjustments. 9. Peer support and co–production ( ) Encourage peer connection with others who share similar experiences; facilitate in the client's preferred sign language when appropriate. ( ) Maintain a network of trained peer mentors; offer small–group rehabilitation opportunities. ( ) Use co–production principles (Montreal Model) with clients, families, clinicians, and communities. 10. Identity, roles, and community engagement ( ) Support clients in developing new social roles and identity. ( ) Link clients to meaningful community activities that foster connection and well–being

**Table:** 

Checklist items not met	Actions required	Responsible Person	Resources needed	Deadline	Status
					
					
					
					
					
					

## 10. Rehabilitation—Tinnitus

**Table:** 

**Tinnitus** 1. Diagnostic assessment ( ) Take a comprehensive tinnitus history. ( ) Assess the tinnitus impact. ( ) Conduct a full audiological assessment (degree of hearing loss, ear pathology). ( ) Communicate clearly and accessibly, considering any hearing or communication needs. ( ) Complete a needs assessment, including therapy preferences. 2. Immediate referral criteria ( ) Urgent mental health referral for tinnitus with distress and suicide risk. ( ) Immediate medical referral for sudden hearing loss or neurological symptoms (e.g., facial weakness). ( ) Otology referral to exclude medical abnormalities and obtain medical opinion on tinnitus management. 3. Medical Management ( ) Management of any underlying pathology 4. Psychological therapies ( ) Ensure talk–based therapies accommodate hearing ability. ( ) Provide written materials matched to literacy level. ( ) Deliver educational counseling to reduce functional impact. ( ) Tailor content to the individual and significant others. ( ) Tinnitus–focused CBT for disturbing tinnitus. 5. Hearing assistance ( ) Counsel that treating hearing loss may reduce tinnitus. ( ) Prioritize hearing loss management. ( ) If aidable: fit hearing aids ensuring maximum audibility for speech and environmental sounds. ( ) If unaidable: consider cochlear implant. 6. Sound-based therapies ( ) Use sound enrichment (amplified/streamed/HA–programmed) alongside psychoeducation or CBT. ( ) Use tinnitus noise generators with extreme caution in severe–profound hearing loss. ( ) Inform users that added sound may mask speech or warning sounds. 7. Other therapies Current research shows potential benefits, but evidence is not yet strong enough for firm recommendations: ( ) Web/app–based self–management ( ) Perceptual/attention training ( ) Neurostimulation/neuromodulation ( ) Acupuncture ( ) Lifestyle changes

**Table:** 

Checklist items not met	Actions required	Responsible Person	Resources needed	Deadline	Status
					
					
					
					
					
					

## 11. Rehabilitation Options—Genetic Hearing Loss and Gene Therapy

**Table:** 

**Genetic hearing loss and gene therapy** 1. Genetic testing (to guide rehabilitation and family management) ( ) Start/continue rehabilitation (communication support, hearing technology, cochlear implantation pathway where appropriate) without waiting for genetic results. ( ) Confirm the hearing phenotype with age-appropriate audiology (behavioral thresholds when possible; ABR/OAE where indicated) and document stability/progression. ( ) Take a targeted history relevant to inherited hearing loss: • Age of onset; progression/fluctuation; symmetry; vestibular symptoms. • Family history (3-generation where feasible), consanguinity, ancestry. • Syndromic “red flags” (vision, renal, cardiac, thyroid, neurodevelopmental, pigmentation, craniofacial). ( ) Record the phenotype in a structured way (to support variant interpretation and genotype–phenotype correlation). Pretest counseling and consent ( ) Offer (or arrange) pretest genetic counseling and document informed consent (patient/parents/carers). ( ) Discuss, in accessible language: • Purpose of testing and what it can/cannot answer. • Possible results (pathogenic/likely pathogenic; negative; VUS) and what each means for management. • Technical limitations (including CNVs/structural variants, low coverage regions, and the potential value of reanalysis). • Secondary/incidental findings policy (especially for exome/genome testing). • Implications for relatives (cascade testing, reproductive risk) and confidentiality considerations. • Data storage/sharing for interpretation and quality assurance; approach to recontacting if interpretation changes. ( ) For children and young people: follow a best-interests framework and seek assent when appropriate. Test selection and requesting ( ) Use a comprehensive hearing-loss NGS panel with CNV calling as first-line where available (WES/WGS is acceptable as first-line in some centers, provided it is delivered within an accredited framework with a virtual panel applied, integrated CNV detection, and appropriate consent and secondary findings policies) ( ) If the phenotype suggests a specific diagnosis, consider targeted testing (e.g., known familial variant). ( ) Escalate to exome/genome sequencing when panel testing is negative, but suspicion remains high, or where syndromic features/complex presentations are present. ( ) Ensure the request includes key clinical details (phenotype, onset, audiology, vestibular findings, imaging, relevant comorbidities) to support interpretation. Variant interpretation and reporting ( ) Ensure variants are classified using recognized standards (e.g., ACMG/AMP), applying hearing-loss-specific specifications where available. ( ) Do not use a variant of uncertain significance (VUS) as the sole basis for irreversible clinical decisions without additional supportive evidence. ( ) Where results are uncertain or discordant with phenotype, consider segregation testing/phase (parents/relatives), phenotype refinement, repeat sampling, and/or reanalysis; discuss in MDT with clinical genetics/molecular pathology input. Posttest actions (rehabilitation impact) ( ) Communicate results in the patient's preferred language/mode (interpreter, captioning, written summary). ( ) Update rehabilitation and counseling based on genotype where relevant (prognosis; syndromic surveillance; cochlear implantation counseling/timing; trial readiness). ( ) Offer cascade testing and reproductive counseling where appropriate. ( ) Document a plan for recontact/reanalysis (per local policy), particularly after negative results in a strong clinical phenotype. 2. Gene therapy (clinical trials and emerging care pathways) ( ) Explain that inner-ear gene therapy is currently gene- and trial-specific; standard rehabilitation (hearing aids, cochlear implantation, communication strategies) remains the default pathway. ( ) Ensure discussion is culturally sensitive and respects Deaf identity and communication preferences. ( ) Confirm a molecular diagnosis consistent with the therapy target (typically pathogenic/likely pathogenic variant(s) with expected inheritance and phenotype consistent with genotype). ( ) Confirm cochlear/anatomical suitability (imaging as per pathway/trial protocol). ( ) Establish likely therapeutic-window considerations (age/onset and duration of deafness; likelihood of viable target cells; trial dependent). ( ) Complete a baseline assessment package (trial dependent), typically including audiology, functional listening/speech measures, vestibular baseline, and relevant medical/anaesthetic assessment. ( ) Provide gene therapy-specific informed consent information (experimental status, uncertainties, procedure-related risks, immune/inflammatory risks, follow-up intensity and long-term surveillance). ( ) Use a defined referral pathway to the relevant gene therapy trial/centers and coordinate MDT input (otology/CI team, audiology, clinical genetics, pediatrics if relevant, speech-language/education support, psychology as needed). ( ) Postintervention: schedule early and repeated audiology follow-up with a consistent test battery; monitor vestibular symptoms/function; record/report adverse events promptly; maintain long-term surveillance (years) for safety/durability. 3. Resources (clinician-facing) ( ) Hereditary Hearing Loss Homepage: hereditaryhearingloss.org ( ) Deafness Variation Database: deafnessvariationdatabase.org ( ) ClinGen Hearing Loss Clinical Domain: clinicalgenome.org/working-groups/clinical-domain/hearing-loss/ ( ) PanelApp (curated diagnostic panels): panelapp.genomicsengland.co.uk

**Table:** 

Checklist items not met	Actions required	Responsible Person	Resources needed	Deadline	Status
					
					
					
					
					
					

## 12. Ongoing Care and Support

**Table:** 

1. Outcome measurement ( ) Use PROMs as the primary method for capturing intervention benefit. ( ) Use speech perception tests to monitor device benefit, recognizing they do not reflect real–world functioning. ( ) Administer an outcome questionnaire to: • Assess functional performance • Identify need for amplification review • Evaluate goal attainment • Identify further rehabilitation needs ( ) Ensure questionnaires include: • Hearing–related domains (activities, participation, device benefit) • Broader domains as appropriate (quality of life, loneliness, work, third–party disability) ( ) Select tools with proven reliability, validity, and sensitivity. 2. Cochlear implant (CI) awareness and referral ( ) Maintain up–to–date knowledge of CI candidacy criteria and consult local CI services when needed. ( ) Test aided speech performance regularly to monitor device benefit and inform CI referral decisions, while recognizing it should not be the sole criterion. ( ) Make onward referrals to other agencies at any stage to ensure holistic support. 3. Follow–up after intervention ( ) Provide follow–up to support optimal device use and management of listening environments. • Prefer face–to–face appointments due to challenges with phone communication. • Visual online follow–up may be appropriate. ( ) Ensure clients have easy access to ongoing care and maintenance, including: • Hearing aid checks • Earmould fit • Frequent tubing changes ( ) Direct clients to additional support services and rehabilitative interventions. ( ) Schedule regular review at least every 3 y, or sooner if hearing changes. ( ) Review more frequently for clients approaching CI candidacy to avoid referral delays. ( ) Proactively discuss cochlear implants with clients who have progressive hearing loss. 4. Professional development and service quality ( ) Maintain and update skills in the audiological management of severe and profound hearing loss. ( ) Engage in shared learning communities (case discussions, PBL, online forums) to support practice, especially where caseload numbers are low

**Table:** 

Checklist items not met	Actions required	Responsible Person	Resources needed	Deadline	Status
					
					
					
					
					
					

**Table:** Appendix 5—QuADS criteria

QuADS criteria	0	1	2	3
1. Theoretical or conceptual underpinning to the research	No mention at all	General reference to broad theories or concepts that frame the study, e.g., key concepts were identified in Introduction section	Identification of specific theories or concepts that frame the study and how these informed the work undertaken, e.g., key concepts were identified in Introduction section and applied to the study	Explicit discussion of the theories or concepts that inform the study, with application of the theory or concept evident through the design, materials and outcomes explored, e.g., key concepts were identified in Introduction section and the application apparent in each element of the study design
2. Statement of research aim/s	No mention at all	Reference to what they sought to achieve embedded within the report but no explicit aims statement	Aims statement made but may only appear in the abstract or be lacking detail	Explicit and detailed statement of aim/s in the main body of report
3. Clear description of research setting and target population	No mention at all	General description of research area but not of the specific research environment, e.g., “in primary care”	Description of research setting is made but is lacking detail, e.g., “in primary care practices in region (x)”	Specific description of the research setting and target population of study, e.g., “nurses and doctors from GP practices in (x) part of (x) city in (x) country”
4. The study design is appropriate to address the stated research aim/s	No research aim/s stated, or the design is entirely unsuitable, e.g., a Y/N item survey for a study seeking to undertake exploratory work of lived experiences	The study design can only address some aspects of the stated research aim/s, e.g., use of focus groups to capture data regarding the frequency and experience of a disease	The study design can address the stated research aim/s, but there is a more suitable alternative that could have been used or used in addition, e.g., addition of a qualitative or quantitative component could strengthen the design	The study design selected appears to be the most suitable approach to attempt to answer the stated research aim/s
5. Appropriate sampling to address the research aim/s	No mention of the sampling approach	Evidence of consideration of the sample required, e.g., the sample characteristics are described and appear appropriate to address the research aim/s	Evidence of consideration of sample required to address the aim, e.g., the sample characteristics are described with reference to the aim/s	Detailed evidence of consideration of the sample required to address the research aim/s, e.g., sample size calculation or discussion of an iterative sampling process with reference to the research aims or the case selected for study
6. Rationale for choice of data collection tool/s	No mention of rationale for data collection tool used	Very limited explanation for choice of data collection tool/s, e.g., based on availability of tool	Basic explanation of rationale for choice of data collection tool/s, e.g., based on use in a prior similar study	Detailed explanation of rationale for choice of data collection tool/s, e.g., relevance to the study aim/s, co-designed with the target population or assessments of tool quality
7. The format and content of the data collection tool is appropriate to address the stated research aim/s	No research aim/s stated and/or data collection tool not detailed	Structure and/or content of tool/s suitable to address some aspects of the research aim/s or to address the aim/s superficially, e.g., single item response that is very general or an open-response item to capture content which requires probing	Structure and/or content of tool/s allow for data to be gathered broadly addressing the stated aim/s but could benefit from refinement, e.g., the framing of survey or interview questions is too broad or focused on one element of the research aim/s	Structure and content of tool/s allow for detailed data to be gathered around all relevant issues required to address the stated research aim/s
8. Description of data collection procedure	No mention of the data collection procedure	Basic and brief outline of data collection procedure, e.g., “using a questionnaire distributed to staff”	States each stage of data collection procedure but with limited detail or states some stages in detail but omits others, e.g., the recruitment process is mentioned but lacks important details	Detailed description of each stage of the data collection procedure, including when, where and how omits others, e.g., the recruitment process is mentioned but lacks important details. Data were gathered such that the procedure could be replicated
9. Recruitment data provided	No mention of recruitment data	Minimal and basic recruitment data, e.g., number of people invited who agreed to take part	Some recruitment data but not a complete account, e.g., number of people who were invited and agreed	Complete data allowing for full picture of recruitment outcomes, e.g., number of people approached, recruited, and who completed with attrition data explained where relevant
10. Justification for analytic method selected	No mention of the rationale for the analytic method chosen	Very limited justification for choice of analytic method selected, e.g., previous use by the research team	Basic justification for choice of analytic method selected, e.g., method used in prior similar research	Detailed justification for choice of analytic method selected, e.g., relevance to the study aim/s or comment around the strengths of the method selected
11. The method of analysis was appropriate to answer the research aim/s	No mention at all	Method of analysis can only address the research aim/s basically or broadly	Method of analysis can address the research aim/s, but there is a more suitable alternative that could have been used or used in addition to offer a stronger analysis	Method of analysis selected is the most suitable approach to attempt to answer the research aim/s in detail, e.g., for qualitative interpretative phenomenological analysis might be considered preferable for experiences vs. content analysis to elicit frequency of occurrence of events
12. Evidence that the research stakeholders have been considered in research design or conduct	No mention at all	Consideration of some of the research stakeholders, e.g., use of pilot study with target sample but no stakeholder involvement in planning stages of study design	Evidence of stakeholder input informing the research, e.g., use of pilot study with feedback influencing the study design/conduct or reference to a project reference group established to guide the research	Substantial consultation with stakeholders identifiable in planning of study design and in preliminary work, e.g., consultation in the stakeholder involvement in planning stages of study design. design/conduct or reference to a project reference group established to guide the research. conceptualization of the research, a project advisory group or evidence of stakeholder input informing the work
13. Strengths and limitations critically discussed	No mention at all	Very limited mention of strengths and limitations with omissions of many key issues, e.g., one or two strengths/limitations mentioned with limited detail	Discussion of some of the key strengths and weaknesses of the study but not complete, e.g., several strengths/limitations explored but with notable omissions or lack of depth of explanation	Thorough discussion of strengths and limitations of all aspects of study including design, methods, data collection tools, sample and analytic approach
